# Highly Pathogenic Avian Influenza A(H5N1) Outbreak in Endangered Cranes, Izumi Plain, Japan, 2022–23 

**DOI:** 10.3201/eid3105.241410

**Published:** 2025-05

**Authors:** Mana Esaki, Kosuke Okuya, Kaori Tokorozaki, Yuko Haraguchi, Taichi Hasegawa, Makoto Ozawa

**Affiliations:** Kagoshima University, Kagoshima, Japan (M. Esaki, K. Okuya, M. Ozawa); Crane Park Izumi, Kagoshima, Japan (K. Tokorozaki, Y. Haraguchi); Matsuoka Research Institute for Science, Tokyo, Japan (T. Hasegawa)

**Keywords:** influenza, highly pathogenic avian influenza, viruses, endangered crane, hooded crane, white-naped crane, environmental water, Izumi Plain, Japan

## Abstract

During the 2022–23 winter season, >1,500 endangered cranes, including hooded cranes (*Grus monacha*) and white-naped cranes (*Grus vipio*), were found debilitated or dead in the Izumi Plain, Japan. Most of the cranes, particularly those collected in November, were infected with highly pathogenic avian influenza (HPAI) H5N1 viruses; virus shedding was higher from the trachea than from the cloaca. The isolation rate from the cranes’ roost water was not markedly higher than that of previous seasons, suggesting that the viruses might be more effectively transmitted among cranes via the respiratory route than through feces. Most wild bird–derived H5N1 isolates were phylogenetically distinct from viruses isolated on nearby chicken farms, indicating limited relationship between the wild bird and chicken isolates. Serologic analyses suggested that herd immunity had little effect on outbreak subsidence. This study deepens our understanding of the circumstances surrounding the unexpected HPAI outbreaks among these endangered cranes.

The Izumi Plain in Kagoshima Prefecture, Japan, is an internationally important wetland registered under the Ramsar Convention. It serves as a crucial wintering site for ≈70% of the global population of hooded cranes (*Grus monacha*) (*1*–*3*) and 20% of white-naped cranes (*Grus vipio*) (*1*,*2*,*4*). To support the conservation of those endangered species, artificial wet paddies are systematically established to provide roosting areas every winter. The roosting sites are also frequented by wild ducks, such as mallards (*Anas platyrhynchos*) and Eurasian wigeons (*Mareca penelope*). Of note, wild waterfowl belonging to the orders Anseriformes and Charadriiformes are known natural reservoirs of avian influenza viruses (AIVs) (*5*,*6*), which raises concerns about possible transmission of AIVs to cranes through shared roost water. We established efficient methods to isolate AIVs from the roost water of cranes and have been monitoring AIVs every winter season since 2012 (*7*–*15*).

We previously isolated highly pathogenic avian influenza (HPAI) subtype H5 viruses from the roost water of cranes and dead or debilitated cranes in the Izumi Plain during the 2014–15, 2016–17, 2020–21, and 2021–22 winter seasons (*12*–*15*). Despite the frequent isolation of HPAI viruses (HPAIVs) from the roost water of cranes, only a few cranes were confirmed to be infected with HPAI H5 virus. For example, during the 2020–21 winter season, we isolated 29 HPAI H5N8 viruses from the roost water of cranes and confirmed 6 cases of HPAI H5N8 infection in dead cranes (*13*). Overall, our previous reports suggested that endangered cranes have low susceptibility to HPAI infection and that the viruses have been circulating primarily among wild ducks in the Izumi Plain.

During the 2022–23 winter season, we encountered a large HPAI outbreak among the endangered cranes in the Izumi Plain. More than 1,500 cranes, most of which were hooded and white-naped cranes, died likely because of HPAI H5N1 infection (*16*). Of note, 11 HPAI outbreaks on chicken farms were reported from the Izumi Plain during the same winter season (*17*), leading us to speculate that the HPAIVs circulating among the endangered cranes may have accidentally and repeatedly invaded the nearby chicken farms. Here, we aimed to describe the details of the unexpected HPAI outbreak among the wild birds in the Izumi Plain, including the results of genetic and serologic analyses of the isolated viruses.

## Materials and Methods

### Ethics Statement

We conducted this study in compliance with the International Guiding Principles for Biomedical Research Involving Animals, Japan’s Act on Conservation of Endangered Species of Wild Fauna and Flora, and the regulations of the Kagoshima University Research Ethics Committee. No animal experiments were conducted as part of this study. Veterinary staff at the crane observatory center collected swab samples for analysis from wild cranes during the wintering period. We carried out all handling procedures to minimize stress and ensure the welfare of the animals.

### Sample Collection

We collected tracheal and cloacal swab samples from 317 debilitated or dead wild birds (295 cranes and 22 other bird species) that were found in the Izumi Plain during November 2022–April 2023. We suspended the collected swab samples in BD Universal Viral Transport Medium (BD, https://www.bd.com) and stored at *<*4°C until further use.

We collected environmental water samples weekly (50 mL/sample) from 2 crane roost sites, a total of 14 spots/week, at the Izumi Plain, following the same protocol as in the 2020–21 (*13*) and 2021–22 (*18*) winter seasons. We collected a total of 196 samples during November 7, 2022–February 20, 2023 and stored them at <4°C until further use (Appendix Figure 1). 

### AIV Isolation

For AIV isolation, we filtered the collected swab samples through a 0.22-μm pore membrane (Sartorius, https://www.sartorius.com) and inoculated into the allantoic cavity of 10-day-old embryonated chicken eggs (2 eggs/sample), as described previously (*8*). We isolated AIVs from the collected water samples as described previously (*14*). In brief, we added chicken red blood cells to roost water samples of cranes that potentially contained AIVs and subsequently inoculated those cells into the allantoic cavity of 10-day-old embryonated chicken eggs (4 eggs/sample). We incubated the inoculated eggs at 37°C for 2 days. We used hemagglutination assay (*8*) to confirm AIV isolation. 

### AIV Gene Detection from Swab Samples and Allantoic Fluids

We extracted RNA from swab samples and allantoic fluids using the innuPREP Virus DNA/RNA Kit (Analytik Jena AG, https://www.analytik-jena.com) and used for influenza A viral gene detection via 1-step real-time reverse transcription PCR RT-PCR with the iTaq Universal SYBR Green One-Step Kit (Bio-Rad Laboratories, https://www.bio-rad.com) and primer sets specific for the H5 hemagglutinin (HA), H7 HA, and matrix (M) genes (*10*). To calculate gene copy number from swab samples, we used plasmids (pCR-Blunt II-TOPO; Thermo Fisher Scientific, https://www.thermofisher.com) inserted with the M gene of A/California/04/2009 (H1N1) for constructing standard curves.

### Targeted Sequencing of H5 HA Cleavage Site

We subjected the sample-derived RNAs in which the H5 HA gene was detected to sequence the H5 HA cleavage site to genetically evaluate viral pathogenicity. Using H5 HA gene–positive samples as a template, we amplified the H5 HA cleavage site through PCR with the PrimeScript One-Step RT-PCR Kit Version Two (TaKaRa Bio Inc., https://www.takara-bio.com) and a primary primer set specific for the full-length H5 HA gene; 5′-CAGGGGTTCAMTCTGTCAAAATGGA-3′ (H5-uni-f_mod) and 5′-ACAAGGGTGTTTTTAACTACAATCTGA-3′ (H5-uni-r_mod), followed by nested PCR with the Tks Gflex DNA Polymerase (TaKaRa Bio Inc.) and a secondary primer set; 5′-AACGTGGAAGAATGGAYTTC-3′ (H5_713F) and 5′-TGTCTGCAGCGTACCCACT-3′ (H5_cle-1149R). We determined nucleotide sequences of the cleavage sites in the H5 HA gene through Sanger sequencing (Azenta Inc., https://www.azenta.com).

### HA and NA Subtyping

To identify potential singular isolates from the allantoic fluids inoculated with the roost water samples of cranes, we subjected the M gene–positive RNA samples to additional scrutiny through real-time RT-PCR with primer sets specific to the H1, H3, H4, H6, and H10 HA genes; those genes have been frequently detected in the Izumi Plain since 2012 (*13*). RNA samples that responded exclusively to a single HA subtype–specific primer set were subjected to reverse transcription using SuperScript IV reverse transcription (Thermo Fisher Scientific) for complementary DNA (cDNA) synthesis. cDNA served as the template for conventional PCR-based HA and NA subtyping using Tks Gflex DNA Polymerase (TaKaRa Bio Inc.), along with a set of subtype-specific primer sets (*19*).

### Comprehensive Sequencing of AIV Genes

Using AIV cDNA as the template, we amplified each gene segment using Tks Gflex DNA Polymerase and KOD One PCR Master Mix-Blue (TOYOBO Co., Ltd., https://www.toyobo-global.com), in conjunction with gene segment-specific primer sets (*20*), through conventional PCR. Subsequently, we determined the nucleotide sequences of the open reading frames for all viral gene segments through nanopore sequencing using the MinION Mk1b system (Oxford Nanopore Technologies, https://nanoporetech.com), as described previously (*21*). In brief, we purified PCR amplicons and performed adaptor ligation using a direct cDNA sequencing kit (Oxford Nanopore Technologies), along with a native barcoding expansion kit (Oxford Nanopore Technologies). We conducted sequencing using the Flongle flow cells (Oxford Nanopore Technologies).

We generated consensus sequences for each gene segment using Geneious Prime version 2021.1.1 (Biomatters Ltd, https://www.geneious.com). The nucleotide sequences have been deposited in the GISAID database (http://www.gisaid.org) (Table; Appendix Tables 2, 3).

**Table:** HI titers for HA antigen in serum samples of cranes tested for influenza A, Izumi Plain, Japan*

Crane sample	Genetic testing for HPAI H5N1	HA antigen titer				
		A/hooded crane/Kagoshima/KU-106/2021 (H5N8) G1 group	A/hooded crane/Kagoshima/KU-5T/2021 (H5N8) G2a subgroup	A/crane/Kagoshima/KU-93/2021 (H5N8) G2a subgroup	A/Environment/Kagoshima/KU-B20/2021 (H5N1) G2b subgroup	A/hooded crane/Kagoshima/KU-105/2022 (H5N1) G2c subgroup
2021–22						
21–8	Neg	ND	ND	ND	4	ND
21–11	Neg	ND	ND	ND	ND	ND
21–14	Neg	8	ND	ND	4	4
21–15	Neg	ND	ND	ND	ND	ND
21–21	Neg	ND	ND	ND	ND	ND
21–19	Neg	ND	ND	ND	ND	ND
21–20	Neg	ND	ND	ND	ND	ND
21–10	Neg	ND	ND	ND	ND	4
21–36	Neg	ND	ND	ND	ND	ND
21–38	Neg	ND	ND	ND	ND	ND
21–51	Neg	ND	ND	ND	ND	ND
21–52	Neg	ND	ND	ND	ND	ND
21–53	Neg	ND	ND	ND	ND	ND
2022–23						
22–105	Pos	4	ND	ND	8	8
22–151	Pos	8	4	4	8	16
22–152	Pos	ND	ND	ND	8	8
22–265	Neg	ND	ND	ND	ND	ND
22–272	Pos	4	ND	ND	4	4
22–278	Neg	ND	ND	ND	ND	ND
22–280	Neg	ND	ND	ND	ND	ND
22–281	Neg	ND	ND	ND	ND	ND
22–282	Neg	ND	ND	ND	ND	ND
22–283	Neg	ND	ND	ND	ND	ND
22–285	Neg	ND	ND	ND	ND	ND
22–286	Neg	ND	ND	ND	ND	ND
22–287	Neg	8	8	4	16	16
22–288	Neg	8	4	ND	16	16
22–289	Neg	ND	ND	ND	ND	4
22–313	Neg	ND	ND	ND	ND	ND
22–318	Neg	ND	ND	ND	ND	ND

*HI, hemagglutination inhibition; HPAI, highly pathogenic avian influenza; ND, specific antibody was not detected (detection limit: 4 HI titer); Neg, negative; Pos, positive.

### Phylogenetic Analyses

We conducted phylogenetic analysis on the nucleotide sequences of viral gene segments obtained from the isolates, along with their representative counterparts retrieved from the GISAID database; the analysis captured the temporal and spatial distribution of AIVs. We aligned the sequences using the MUSCLE program (*22*) and constructed phylogenetic trees for each viral gene using the maximum-likelihood method in MEGA 7 software (*23*) with a bootstrapping set of 1,000 replicates.

### Hemagglutination Inhibition Assay

We evaluated neutralizing antibody titers against HPAI H5 viruses using hemagglutination inhibition (HI) assays as described in the World Health Organization standards (*24*). We treated 30 crane serum samples with receptor destroying enzyme (Denka-Seiken, https://www.denka.co.jp), according to the manufacturer’s instructions. Those samples were collected from the debilitated or dead wild cranes found in the Izumi Plain during November 2021–March 2023 (Appendix Table 4). For the viral antigens, we selected 5 genetically diverse HPAI H5 viruses isolated from the Izumi Plain: A/hooded crane/Kagoshima/KU-106/2021 (H5N8) (G1 group), A/hooded crane/Kagoshima/KU-5T/2021 (H5N8) (G2a subgroup), A/crane/Kagoshima/KU-93/2021 (H5N8) (G2a subgroup), A/environment/Kagoshima/KU-B20/2021 (H5N1) (G2b subgroup), and A/hooded crane/Kagoshima/KU-105/2022 (H5N1) (G2c subgroup) (*7*,*13*,*14*).

## Results

### Large-Scale Mortality of Endangered Cranes in the Izumi Plain

**Figure 1:**
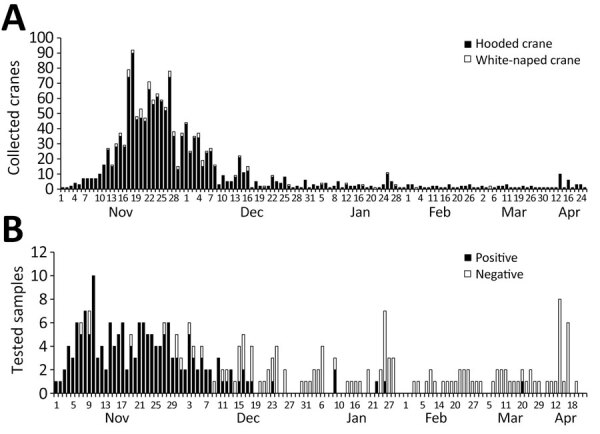
Numbers of collected cranes in the Izumi Plain, Japan, and the samples subjected to genetic testing for influenza A viral matrix (M) and H5 hemagglutinin (HA) genes during the 2022–23 winter season. A) Number of debilitated and dead cranes collected in the Izumi Plain per day. B) Number of cranes subjected to genetic testing for influenza A viral M and H5 HA genes per day.

**Figure 2:**
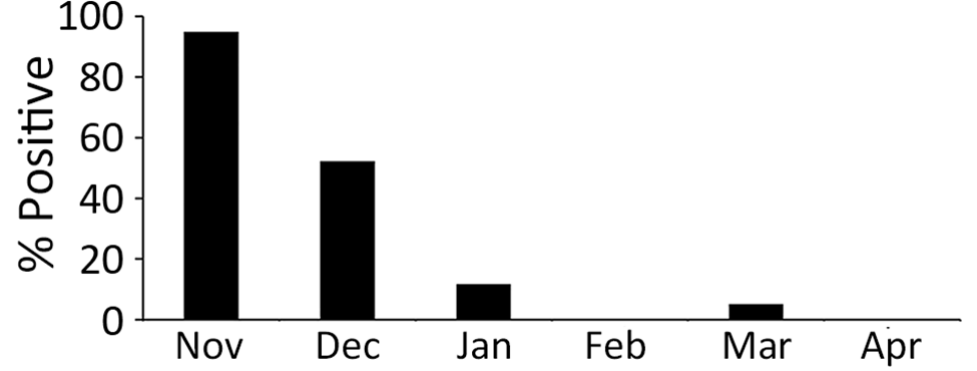
Monthly rate of highly pathogenic avian influenza A(H5N1) infection among tested cranes in the Izumi Plain, Japan, 2022–23 winter season.

During the 2022–23 winter season, 1,425 hooded and 79 white-naped cranes were found debilitated or dead in the Izumi Plain (Appendix Table 1). The peak daily collection number reached 92 cranes on November 18, 2022 (Figure 1, panel A). The number of collected cranes gradually decreased; daily collections averaged <10 cranes from mid-December onward. We collected swab samples from 295/1,504 cranes and subjected them to genetic testing for influenza A viral M and H5 HA genes. We further analyzed the H5 HA-positive samples by sequencing the H5 HA cleavage site, revealing that 170 (57.6%) cranes tested positive for HPAI H5N1 viruses (Figure 1, panel B). Although most (132/138 [95.7%]) of the samples collected in November were positive for HPAI H5N1 viruses, the positivity ratio began to decrease in samples collected in December (Figure 2). Of note, a swab sample collected on March 20, 2023, tested positive, indicating the circulation of HPAIVs by the end of the winter season in the Izumi Plain.

To isolate HPAI viruses from the samples that tested positive for HPAI H5N1, we inoculated all swab samples from the 170 cranes into embryonated chicken eggs. We isolated 136 HPAI H5N1 viruses from the swab samples (Appendix Table 1).

### HPAI H5N1 Virus Shedding of in Endangered Cranes

**Figure 3:**
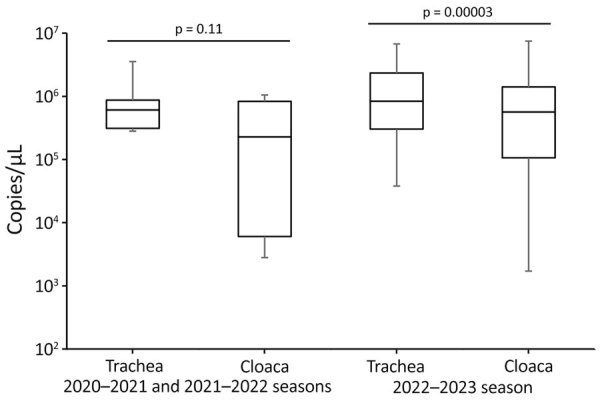
Box-and-whisker plot showing the distribution of copy numbers of the avian influenza virus (AIV) matrix (M) gene in swabs from AIV gene–positive cranes from the 2022–23 winter season compared with the combined 2020–21 and 2021–22 seasons in the Izumi Plain, Japan. We used RNA extracted from each AIV gene–positive swab sample for quantifying the copy number of the AIV M gene using real-time reverse transcription PCR. Boxes represent interquartile ranges; horizontal lines inside boxes indicates median; whiskers indicate maximum and minimum values. We evaluated significant differences between groups by paired *t*-test; p<0.05 was considered statistically significant.

To compare oral and cloacal virus shedding from the HPAI H5N1–infected cranes, we measured numbers of copies of influenza A viral M gene in their tracheal and cloacal swab specimens individually. However, we combined the tracheal and cloacal swab specimens collected from the same cranes before analysis beginning November 22, 2022, because of the drastic increase in the number of cases and shortage of equipment. In total, we tested tracheal and cloacal swab samples from 86 cranes collected during November 1–21, 2022, in this study (Figure 3; Appendix Table 2, Figure 2). In addition, we included RNA extracted from the swab samples of 7 HPAI-infected cranes from previous seasons as comparison controls (*13*,*14*). We found that the gene copy numbers in tracheal swab samples were significantly higher than those in cloacal swab samples during the 2022–23 season, as confirmed by a paired *t*-test (p<0.05); however, we observed no significant difference during the 2020–21 and 2021–22 seasons (Figure 3). Our results suggest that virus shedding in the cranes was more pronounced in the trachea than in the cloaca. 

### Limited Number of HPAI Virus Isolates from Roost Water 

**Figure 4:**
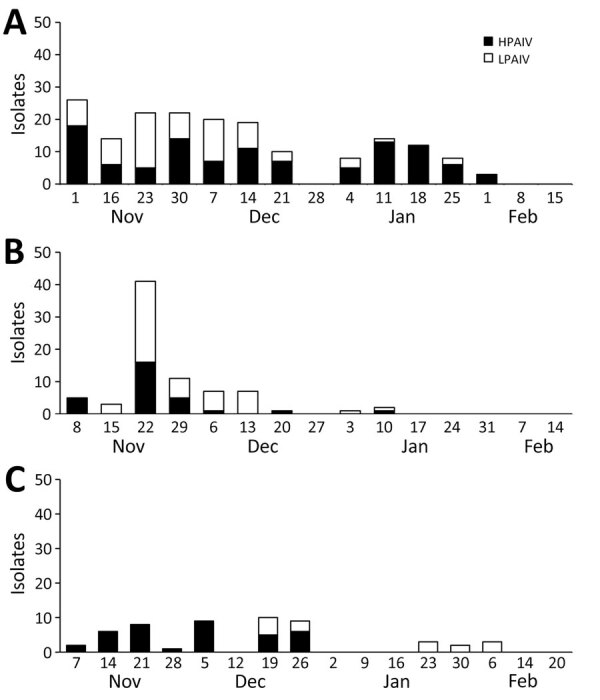
Avian influenza virus (AIV) isolation from roost water of cranes in the Izumi Plain, Japan. Weekly representation illustrates the numbers of AIV-positive allantoic fluid samples during the 2020–21 (A) (*13*), 2021–22 (B) (*18*), and 2022–23 (C) winter seasons. Bars show AIV isolates containing HPAIV H5 and those containing LPAIV. The weekly chart displays the number of AIV-positive allantoic fluid samples. HPAIV, highly pathogenic avian influenza virus; LPAIV, low pathogenicity avian influenza virus.

A total of 53 of 196 allantoic fluid samples acquired from the eggs inoculated with roost water samples of cranes tested positive for influenza A viral M gene. We identified HA genes of multiple subtypes, including the H5 genes, in 15/53 samples; we excluded those 15 samples from further genetic analyses. Genetic analyses of the HA and NA genes revealed that the remaining 38 AIVs were singular isolates; we classified 24 as subtype H5N1, 8 as H3N8, and 6 as H10N6 (Appendix Table 3). Despite the large HPAI H5N1 outbreak among the endangered cranes, the number of HPAIV isolates from the roost water of cranes during the 2022–23 season was not markedly higher than that in previous seasons; we identified 107 (42.46%) HPAIV-containing isolates out of 252 samples during the 2020–21 season (*13*) and 29 (14.80%) HPAIV-containing isolates out of 196 samples during the 2021–22 winter season (*18*) (Figure 4). The ratio of HPAIV-containing isolates during the 2022–23 season (37/196 [18.88%] samples) was significantly lower than that in the 2020–21 season (p<0.05 by χ^2^ test); no significant difference was observed between the 2022–23 and 2021–22 seasons. Those results suggest that virus shedding from the HPAIV-infected cranes into environmental water was minimal. Furthermore, considering that virus shedding in the cranes was more evident in the tracheal swab than in the cloacal swab samples, HPAI H5N1 viruses might be effectively transmitted via the respiratory route among the cranes, rather than through waterborne transmission.

### AIV Isolation from Other Bird Species 

We isolated 2 HPAI H5N1 virus strains, A/northern pintail/Kagoshima/KU-64/2022 (H5N1) and A/black kite/Kagoshima/KU-140/2022 (H5N1), and 1 low pathogenicity avian influenza (LPAI) H11N9 virus, A/mallard/Kagoshima/KU-131/2022 (H11N9), from 22 wild birds other than cranes collected in the Izumi Plain (Appendix Table 2). The results indicated that HPAI H5N1 viruses were spread not only among cranes but also among other species of wild birds in the Izumi Plain during the 2022–23 season.

### Detection of Multiple Genotypes of HPAI H5N1 Viruses

**Figure 5:**
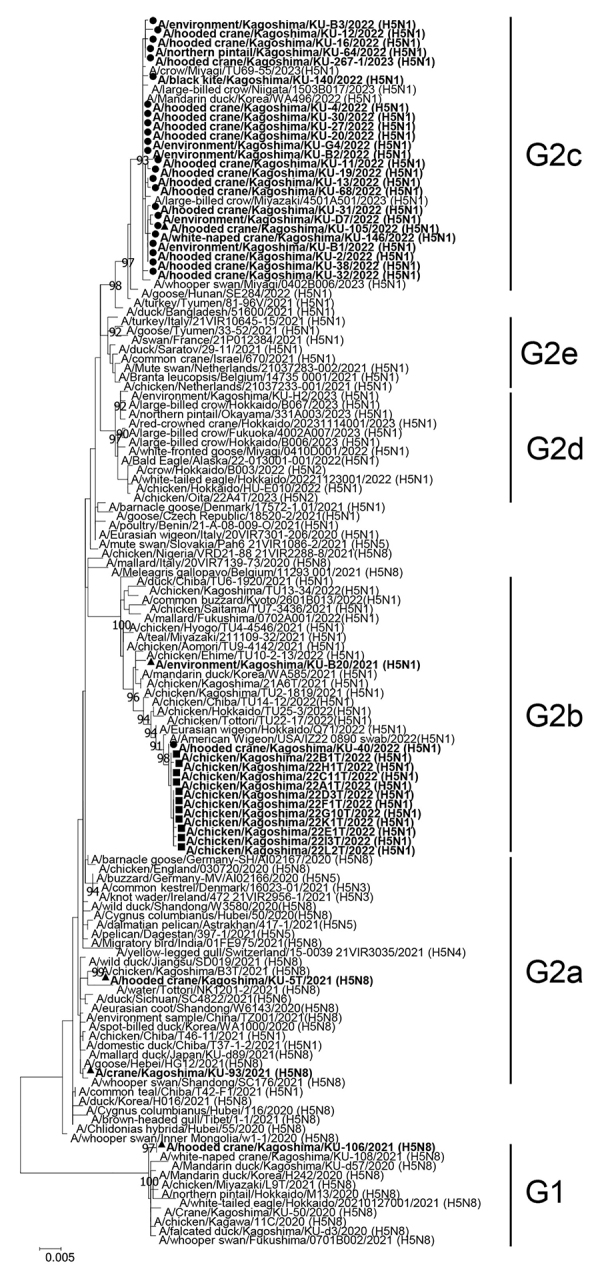
Phylogenetic tree of H5 hemagglutinin (HA) genes from avian influenza virus gene–positive cranes from the 2022–23 winter season in the Izumi Plain, Japan, compared with reference sequences. Tree was constructed using the maximum-likelihood method with a bootstrapping set of 1,000 replicates. Nodes with bootstrap values >90% are shown. Circles indicate isolates from wild birds and cranes’ roost water during the 2022–23 winter season. Squares indicate isolates from chickens during the same season. Triangles indicate H5 HA genes of highly pathogenic avian influenza viruses used for the hemagglutination inhibition assay. Scale bar indicates nucleotide substitutions per site.

**Figure 6:**
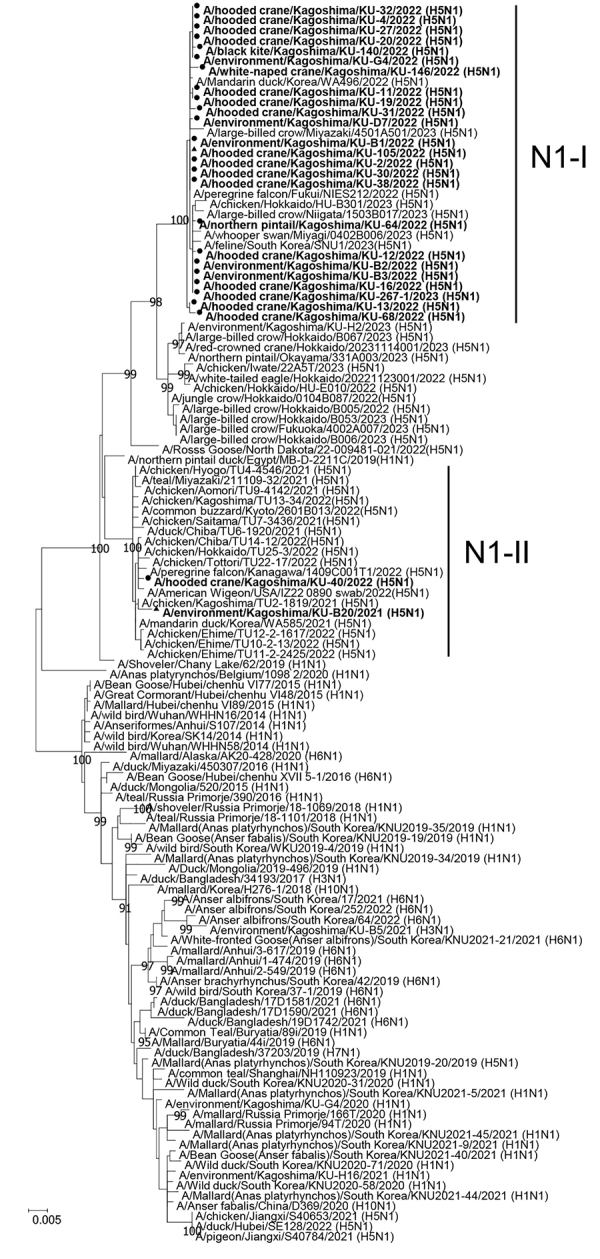
Phylogenetic trees of N1 neuraminidase (NA) genes from avian influenza virus gene–positive cranes from the 2022–23 winter season in the Izumi Plain, Japan, compared with reference sequences. Tree was constructed using the maximum-likelihood method with a bootstrapping set of 1,000 replicates. Nodes with bootstrap values >90% are shown. Circles indicate isolates from the 2022–23 winter season. Triangles indicate H5 NA genes of highly pathogenic avian influenza viruses used for the hemagglutination inhibition assay. Scale bar indicates nucleotide substitutions per site.

We phylogenetically genotyped the HPAI H5N1 isolates for genetic characterization of each gene segment (Appendix Table 5); we constructed phylogenetic trees for representative H5N1 isolates from each genotype (Figures 5,6; Appendix Figure 3). We categorized the HA genes of the H5N1 isolates, except those of A/hooded crane/Kagoshima/KU-40/2022 (H5N1), into the G2c subgroup (Figure 5). The HA gene of A/hooded crane/Kagoshima/KU-40/2022 (H5N1) formed a cluster with HPAI H5 viruses mainly isolated from chicken farms in Japan, including 9 farms in the Izumi Plain (https://www.maff.go.jp/j/syouan/douei/tori/220929.html#2) (*25*), during the 2022–23 winter season and was categorized into the G2b subgroup (Figure 5). Those results indicate that HPAI H5N1 virus strains circulating among the crane population were genetically distinct from those isolated from chicken farms, suggesting that the invasion of HPAIV in the farms was not attributable to the crane species.

Genetic analyses revealed that the N1 NA genes could be classified into 2 genetic clusters, N1-I and N1-II (Figure 6). Similarly, we classified the PB2, NP, and NS genes into 6 genetic clusters, the PB1 and PA genes into 7 genetic clusters, and the M gene into 5 genetic clusters (Appendix Table 5, Figure 3). According to the genetic clusters of the remaining 6 gene segments, we classified HPAI H5N1 virus strains isolated in this study into 13 genotypes (H5N1-I to -XIII) (Appendix Table 5). Furthermore, genetic analyses of LPAI viruses revealed that 4 genotypes of H3N8 (H3N8-I to -IV) and 2 genotypes of H10N6 (H10N6-I and -II) were introduced into the Izumi Plain during the 2022–23 winter season (Appendix Table 5, Figure 4). We found that multiple genotypes of H5N1 HPAIVs were introduced into the Izumi Plain in the early winter season. Of note, the PA gene of A/hooded crane/Kagoshima/KU-38/2022 (H5N1), whose HA gene was categorized to the G2c subgroup, was almost identical to that of A/hooded crane/Kagoshima/KU-40/2022 (H5N1), whose HA gene was categorized into the G2b subgroup (Appendix Figure 3, panel C). Those results suggest that a genetic reassortment event occurred among the H5N1 HPAIVs circulating in the Izumi Plain.

### HI Titer against H5 HPAIVs in Crane Serum

To assess herd immunity against HPAIV infection among endangered cranes, we measured antibody titers using HI assay in 30 crane serum samples: 13 serum samples collected during the 2021–22 winter season and 17 serum samples collected during the 2022–23 winter season (Appendix Table 4). Of note, 14/17 serum samples were collected during January–February 2023, after the large outbreak of HPAI H5N1 infection in the Izumi Plain. Among 17 cranes from which serum samples collected during the 2022–23 winter season, 4 cranes (i.e., cranes 22-105, 22-151, 22-152, and 22-272) had been confirmed to be infected with H5N1 HPAIV. We selected 5 phylogenetically distant HPAI H5 viruses as viral antigens: A/hooded crane/Kagoshima/KU-106/2021 (H5N8) from G1 group, A/hooded crane/Kagoshima/KU-5T/2021 (H5N8) from G2a subgroup, A/crane/Kagoshima/KU-93/2021 (H5N8) from G2a subgroup, A/environment/Kagoshima/KU-B20/2021 (H5N1) from G2b subgroup, and A/hooded crane/Kagoshima/KU-105/2022 (H5N1) from G2c subgroup (Figures 5,6).

We detected specific antibodies against >1 HPAI H5 viruses in 3 (23.1%) of 13 serum samples collected during the 2021–22 winter season and in 7 (41.2%) of 17 samples from the 2022–23 winter season (Table). Although the antibody-positive rate in crane serum samples collected during the 2022–23 winter season was higher than that for samples collected during the 2021–22 winter season, it was unexpectedly lower even after the large HPAI outbreak (*26*–*28*). Those results suggest the limited contribution of herd immunity to the subsidence of the outbreak among endangered cranes.

## Discussion

We report a large HPAI outbreak among endangered cranes. In total, 1,504 debilitated or dead endangered cranes were collected during the 2022–23 winter season in the Izumi Plain, Japan (Figure 1, panel A). We confirmed that most of the debilitated or dead cranes, particularly those collected in November (95.7%), were infected with H5N1 viruses (Figure 1, panel B).

In wild ducks, LPAI viruses mainly replicate in the intestine and are thus shed in the feces (*5*,*6*). Environmental water contaminated with the feces of wild ducks has been effectively used for AIV surveillance in the Izumi Plain since 2012 (*10*,*11*,*13*,*14*,*18*,*29*). During the 2022–23 winter season, we isolated 24 HPAI H5N1 virus strains from the roost water of cranes (Appendix Table 3). Despite the large outbreak of HPAI H5N1 among endangered cranes, the number of water HPAI isolates during the 2022–23 winter season was not markedly higher than that in previous seasons (Figure 4). In addition, the gene copy numbers of HPAIVs in tracheal swabs from the infected cranes were higher than those in cloacal swabs (Figure 3), indicating high virus shedding in the respiratory tracts. Overall, these findings suggest that HPAI H5N1 circulating among the endangered cranes during the 2022–23 season might have been transmitted more efficiently via the respiratory route rather than through environmental water.

We isolated 162 HPAI H5N1 viruses of the G2c subgroup from wild birds and roost water of cranes during the 2022–23 winter season; we categorized 1 isolate, A/hooded crane/Kagoshima/KU-40/2022 (H5N1), in the G2b subgroup. Of note, all HPAIVs isolated from 9 chicken farms in the Izumi Plain during the 2022–23 winter season belonged to the G2b subgroup (Figure 5) (*22*). Those results suggest that the HPAI outbreaks in the chicken farms in the Izumi Plain are not closely associated with the HPAIVs circulating among nearby wild birds. Although our findings do not diminish the importance of wild birds as a major source of HPAIVs causing outbreaks on chicken farms (*29*), further studies are urgently needed to identify other factors contributing to HPAIV invasion in chicken farms.

Phylogenetic analyses revealed that the H5 HA gene of the G2c subgroup was located on a lower branch of the G2e subgroup (Figure 5), indicating that the H5 HPAIVs of G2c subgroup are progeny viruses of the G2e subgroup, which had caused large-scale mortality of common cranes (*Grus grus*) in Israel in 2021 (https://www.woah.org/app/uploads/2022/01/hpai-situation-report-20220117.pdf). Although specific factors affecting the pathogenicity and transmissibility of HPAI H5 in crane species remain unclear, the H5 subtype of G2e and G2c subgroups may share genetic background related to high mortality rates in crane species.

Serologic analyses revealed that only 41.2% of crane serum samples collected during the 2022–23 winter season were seropositive for HPAI H5 circulating in the same crane populations after the large HPAI outbreak in that season (Table). A limitation of our study is that the sample size was insufficient and the samples might contain potential biases. Nevertheless, the results suggest that the HPAI H5 outbreak among cranes has not subsided because of herd immunity resulting from widespread infection in the Izumi Plain. One possible factor contributing to the low seropositive rate is the dispersal of dense crane gatherings at their roosts around mid-November, which likely reduced opportunities for uninfected birtds to contract the infection. Therefore, the potential for another HPAI H5 outbreak among endangered cranes remains. Preventive measures, including intentional dispersal of crane wintering sites to avoid excessive concentration of birds in a single location (*30*–*33*), are urgently needed to protect the endangered cranes in the Izumi Plain.

In conclusion, HPAI H5N1 viruses caused large-scale mortality of endangered cranes, including hooded cranes and white-naped cranes, in the Izumi Plain of Japan during the 2022–23 winter season. Our findings suggest that H5N1 circulated mainly via the respiratory route, but not the environmental waterborne route, among the endangered cranes. Most of the HPAIVs circulating in wild birds were genetically distant from those isolated from chicken farms. In addition, the endangered crane populations have not developed herd immunity against H5N1. Our study provides new insights into understanding the circumstances surrounding HPAI H5N1 outbreaks among endangered cranes and could help in their conservation.

Appendix Additional information about highly pathogenic avian influenza A(H5N1) outbreak in endangered cranes, Izumi Plain, Japan, 2022–23.
