# Supplementary material for: Highly Pathogenic Avian Influenza A(H5N1) Outbreak in Endangered Cranes, Izumi Plain, Japan, 2022–23
Source: Emerg Infect Dis. 2025 May;31(5):937–47. doi: 10.3201/eid3105.241410 (PMC12044252; doi:10.3201/eid3105.241410)
Supplement: Appendix — Additional information about highly pathogenic avian influenza A(H5N1) outbreak in endangered cranes, Izumi Plain, Japan, 2022–23. [file 24-1410-Techapp-s1.pdf]

Article DOI: https://doi.org/10.3201/eid3105.241410

EID cannot ensure accessibility for supplementary materials supplied by authors. Readers who have difficulty accessing supplementary content should contact the authors for assistance.

Highly Pathogenic Avian Influenza A (H5N1) Outbreak in Endangered Cranes, Izumi Plain, Japan, 2022–23

Appendix

Appendix Table 1. Numbers of cranes tested in study of highly pathogenic avian influenza A (H5N1) outbreak, Izumi Plain, Japan, 2022–23*

Characteristic	No. collected, N =1,504
Cranes tested for influenza A viral M gene, no. positive/total	170/295
Cranes tested for influenza A viral H5 HA gene, no. positive/total	170/295
Cranes tested for targeted sequencing of H5 HA cleavage site, no. HPAIV-typed/total	170/170
Cranes tested for H5N1 HPAIV isolation, no. isolated/total	136/170

*HA, hemagglutinin; HPAIV, highly pathogenic avian influenza virus; M, matrix protein.

Appendix Table 2. AIV gene–positive cranes and other wild birds collected in study of highly pathogenic avian influenza A (H5N1) outbreak, Izumi Plain, Japan, 2022–23*

Host species	Sample ID	Collection date	Age	Vital status	RT-PCR	Virus isolation	Isolate	GISAID accession no.
Hooded crane	22–2	November 1, 2022	Adult	Debilitated, and died within a day	+	+	A/hooded crane/Kagoshima/KU-2/2022 (H5N1)	EPI_ISL_18472589
Hooded crane	22–3	November 2, 2022	Adult	Debilitated, and died within a day	+	+	A/hooded crane/Kagoshima/KU-3/2022 (H5N1)	EPI_ISL_18472590
Hooded crane	22–4	November 3, 2022	Adult	Dead	+	+	A/hooded crane/Kagoshima/KU-4/2022 (H5N1)	EPI_ISL_18472591
Hooded crane	22–5	November 3, 2022	Adult	Dead	+	+	A/hooded crane/Kagoshima/KU-5/2022 (H5N1)	EPI_ISL_18472592
Hooded crane	22–6	November 4, 2022	Unknown	Dead	+	+	A/hooded crane/Kagoshima/KU-6/2022 (H5N1)	EPI_ISL_18472593
Hooded crane	22–7	November 4, 2022	Adult	Debilitated	+	+	A/hooded crane/Kagoshima/KU-7/2022 (H5N1)	EPI_ISL_18472594
Hooded crane	22–8	November 4, 2022	Adult	Dead	+	+	A/hooded crane/Kagoshima/KU-8/2022 (H5N1)	EPI_ISL_18472595
Hooded crane	22–9	November 4, 2022	Adult	Debilitated	+	+	A/hooded crane/Kagoshima/KU-9/2022 (H5N1)	EPI_ISL_18472596
Hooded crane	22–10	November 5, 2022	Adult	Debilitated	+	+	A/hooded crane/Kagoshima/KU-10/2022 (H5N1)	EPI_ISL_18472597
Hooded crane	22–11	November 5, 2022	Adult	Dead	+	+	A/hooded crane/Kagoshima/KU-11/2022 (H5N1)	EPI_ISL_18472598
Hooded crane	22–12	November 5, 2022	Adult	Dead	+	+	A/hooded crane/Kagoshima/KU-12/2022 (H5N1)	EPI_ISL_18472599

Host species	Sample ID	Collection date	Age	Vital status	RT-PCR	Virus isolation	Isolate	GISAID accession no.
Hooded crane	22–13	November 6, 2022	Adult	Debilitated, and died within a day	+	+	A/hooded crane/Kagoshima/KU-13/2022 (H5N1)	EPI_ISL_18472600
Hooded crane	22–14	November 6, 2022	Adult	Dead	+	+	A/hooded crane/Kagoshima/KU-14/2022 (H5N1)	EPI_ISL_18472601
Hooded crane	22–15	November 6, 2022	Juvenile	Debilitated	+	+	A/hooded crane/Kagoshima/KU-15/2022 (H5N1)	EPI_ISL_18472602
Hooded crane	22–16	November 6, 2022	Adult	Debilitated	+	+	A/hooded crane/Kagoshima/KU-16/2022 (H5N1)	EPI_ISL_18472603
Hooded crane	22–18	November 6, 2022	Adult	Dead	+	-		
Hooded crane	22–19	November 6, 2022	Juvenile	Debilitated	+	+	A/hooded crane/Kagoshima/KU-19/2022 (H5N1)	EPI_ISL_18472604
Hooded crane	22–20	November 7, 2022	Adult	Dead	+	+	A/hooded crane/Kagoshima/KU-20/2022 (H5N1)	EPI_ISL_18472605
Hooded crane	22–21	November 7, 2022	Adult	Debilitated, and died within a day	+	-		
Hooded crane	22–22	November 7, 2022	Adult	Dead	+	+	A/hooded crane/Kagoshima/KU-22/2022 (H5N1)	EPI_ISL_18472606
Hooded crane	22–23	November 7, 2022	Adult	Dead	+	+	A/hooded crane/Kagoshima/KU-23/2022 (H5N1)	EPI_ISL_18472607
Hooded crane	22–24	November 7, 2022	Adult	Dead	+	+	A/hooded crane/Kagoshima/KU-24/2022 (H5N1)	EPI_ISL_18472608
Hooded crane	22–26	November 8, 2022	Adult	Dead	+	+	A/hooded crane/Kagoshima/KU-26/2022 (H5N1)	EPI_ISL_18472609
Hooded crane	22–27	November 8, 2022	Adult	Dead	+	+	A/hooded crane/Kagoshima/KU-27/2022 (H5N1)	EPI_ISL_18472610
Hooded crane	22–28	November 8, 2022	Adult	Dead	+	+	A/hooded crane/Kagoshima/KU-28/2022 (H5N1)	EPI_ISL_18472611
Hooded crane	22–29	November 8, 2022	Adult	Dead	+	+	A/hooded crane/Kagoshima/KU-29/2022 (H5N1)	EPI_ISL_18472612
Hooded crane	22–30	November 8, 2022	Adult	Debilitated	+	+	A/hooded crane/Kagoshima/KU-30/2022 (H5N1)	EPI_ISL_18472613
Hooded crane	22–31	November 8, 2022	Adult	Dead	+	+	A/hooded crane/Kagoshima/KU-31/2022 (H5N1)	EPI_ISL_18472614
Hooded crane	22–32	November 8, 2022	Adult	Dead	+	+	A/hooded crane/Kagoshima/KU-32/2022 (H5N1)	EPI_ISL_18472615
Hooded crane	22–33	November 9, 2022	Adult	Dead	+	+	A/hooded crane/Kagoshima/KU-33/2022 (H5N1)	EPI_ISL_18472616
Hooded crane	22–35	November 9, 2022	Adult	Dead	+	+	A/hooded crane/Kagoshima/KU-35/2022 (H5N1)	EPI_ISL_18472617
Hooded crane	22–36	November 9, 2022	Adult	Dead	+	+	A/hooded crane/Kagoshima/KU-36/2022 (H5N1)	EPI_ISL_18472618
Hooded crane	22–38	November 9, 2022	Adult	Debilitated	+	+	A/hooded crane/Kagoshima/KU-38/2022 (H5N1)	EPI_ISL_18472619
Hooded crane	22–39	November 9, 2022	Adult	Dead	+	+	A/hooded crane/Kagoshima/KU-39/2022 (H5N1)	EPI_ISL_18472620

Host species	Sample ID	Collection date	Age	Vital status	RT-PCR	Virus isolation	Isolate	GISAID accession no.
Hooded crane	22–40	November 10, 2022	Adult	Dead	+	+	A/hooded crane/Kagoshima/KU-40/2022 (H5N1)	EPI_ISL_18472621
Hooded crane	22–41	November 10, 2022	Adult	Dead	+	+	A/hooded crane/Kagoshima/KU-41/2022 (H5N1)	EPI_ISL_18472622
Hooded crane	22–42	November 10, 2022	Adult	Dead	+	+	A/hooded crane/Kagoshima/KU-42/2022 (H5N1)	EPI_ISL_18472623
Hooded crane	22–43	November 10, 2022	Adult	Dead	+	+	A/hooded crane/Kagoshima/KU-43/2022 (H5N1)	EPI_ISL_18472624
Hooded crane	22–44	November 10, 2022	Adult	Dead	+	+	A/hooded crane/Kagoshima/KU-44/2022 (H5N1)	EPI_ISL_18472625
Hooded crane	22–45	November 10, 2022	Adult	Dead	+	+	A/hooded crane/Kagoshima/KU-45/2022 (H5N1)	EPI_ISL_18472626
Hooded crane	22–46	November 10, 2022	Adult	Dead	+	+	A/hooded crane/Kagoshima/KU-46/2022 (H5N1)	EPI_ISL_18472627
Hooded crane	22–47	November 10, 2022	Adult	Dead	+	+	A/hooded crane/Kagoshima/KU-47/2022 (H5N1)	EPI_ISL_18472628
Hooded crane	22–48	November 10, 2022	Adult	Dead	+	+	A/hooded crane/Kagoshima/KU-48/2022 (H5N1)	EPI_ISL_18472629
Hooded crane	22–50	November 10, 2022	Adult	Debilitated, and died within a day	+	+	A/hooded crane/Kagoshima/KU-50/2022 (H5N1)	EPI_ISL_18472630
Hooded crane	22–51	November 11, 2022	Adult	Debilitated	+	+	A/hooded crane/Kagoshima/KU-51/2022 (H5N1)	EPI_ISL_18472631
Hooded crane	22–52	November 11, 2022	Adult	Dead	+	+	A/hooded crane/Kagoshima/KU-52/2022 (H5N1)	EPI_ISL_18472632
Hooded crane	22–53	November 11, 2022	Adult	Debilitated	+	+	A/hooded crane/Kagoshima/KU-53/2022 (H5N1)	EPI_ISL_18472633
Hooded crane	22–54	November 12, 2022	Adult	Dead	+	+	A/hooded crane/Kagoshima/KU-54/2022 (H5N1)	EPI_ISL_18472634
Hooded crane	22–55	November 12, 2022	Adult	Debilitated, and died within a day	+	+	A/hooded crane/Kagoshima/KU-55/2022 (H5N1)	EPI_ISL_18472635
Hooded crane	22–56	November 12, 2022	Adult	Dead	+	+	A/hooded crane/Kagoshima/KU-56/2022 (H5N1)	EPI_ISL_18472636
Hooded crane	22–57	November 12, 2022	Adult	Dead	+	+	A/hooded crane/Kagoshima/KU-57/2022 (H5N1)	EPI_ISL_18472637
Hooded crane	22–58	November 13, 2022	Adult	Dead	+	+	A/hooded crane/Kagoshima/KU-58/2022 (H5N1)	EPI_ISL_18472638
Hooded crane	22–59	November 13, 2022	Adult	Debilitated, and died within a day	+	+	A/hooded crane/Kagoshima/KU-59/2022 (H5N1)	EPI_ISL_18472639
Hooded crane	22–60	November 14, 2022	Adult	Debilitated	+	+	A/hooded crane/Kagoshima/KU-60/2022 (H5N1)	EPI_ISL_18472640
Hooded crane	22–61	November 14, 2022	Adult	Debilitated	+	+	A/hooded crane/Kagoshima/KU-61/2022 (H5N1)	EPI_ISL_18472641
White-naped crane	22–62	November 14, 2022	Adult	Dead	+	+	A/white-naped crane/Kagoshima/KU-62/2022 (H5N1)	EPI_ISL_18472642

Host species	Sample ID	Collection date	Age	Vital status	RT-PCR	Virus isolation	Isolate	GISAID accession no.
Hooded crane	22–63	November 14, 2022	Adult	Dead	+	+	A/hooded crane/Kagoshima/KU-63/2022 (H5N1)	EPI_ISL_18472643
Hooded crane	22–65	November 14, 2022	Adult	Dead	+	+	A/hooded crane/Kagoshima/KU-65/2022 (H5N1)	EPI_ISL_18472583
White-naped crane	22–66	November 14, 2022	Adult	Dead	+	+	A/white-naped crane/Kagoshima/KU-66/2022 (H5N1)	EPI_ISL_18472644
Hooded crane	22–67	November 15, 2022	Adult	Debilitated	+	+	A/hooded crane/Kagoshima/KU-67/2022 (H5N1)	EPI_ISL_18472645
Hooded crane	22–68	November 15, 2022	Adult	Debilitated	+	+	A/hooded crane/Kagoshima/KU-68/2022 (H5N1)	EPI_ISL_18472646
Hooded crane	22–69	November 15, 2022	Adult	Dead	+	+	A/hooded crane/Kagoshima/KU-69/2022 (H5N1)	EPI_ISL_18472647
Hooded crane	22–71	November 15, 2022	Adult	Debilitated	+	+	A/hooded crane/Kagoshima/KU-71/2022 (H5N1)	EPI_ISL_18472648
Hooded crane	22–72	November 16, 2022	Adult	Dead	+	-		
Hooded crane	22–73	November 16, 2022	Adult	Dead	+	+	A/hooded crane/Kagoshima/KU-73/2022 (H5N1)	EPI_ISL_18472649
Hooded crane	22–74	November 16, 2022	Adult	Dead	+	+	A/hooded crane/Kagoshima/KU-74/2022 (H5N1)	EPI_ISL_18472650
Hooded crane	22–75	November 16, 2022	Juvenile	Dead	+	+	A/hooded crane/Kagoshima/KU-75/2022 (H5N1)	EPI_ISL_18472651
Hooded crane	22–76	November 16, 2022	Adult	Debilitated	+	+	A/hooded crane/Kagoshima/KU-76/2022 (H5N1)	EPI_ISL_18472652
Hooded crane	22–77	November 17, 2022	Adult	Dead	+	+	A/hooded crane/Kagoshima/KU-77/2022 (H5N1)	EPI_ISL_18472653
White-naped crane	22–78	November 17, 2022	Adult	Dead	+	+	A/white-naped crane/Kagoshima/KU-78/2022 (H5N1)	EPI_ISL_18472654
Hooded crane	22–79	November 17, 2022	Adult	Dead	+	+	A/hooded crane/Kagoshima/KU-79/2022 (H5N1)	EPI_ISL_18472655
Hooded crane	22–80	November 17, 2022	Adult	Debilitated	+	+	A/hooded crane/Kagoshima/KU-80/2022 (H5N1)	EPI_ISL_18472656
White-naped crane	22–81	November 17, 2022	Adult	Debilitated, and died within a day	+	+	A/white-naped crane/Kagoshima/KU-81/2022 (H5N1)	EPI_ISL_18472657
Hooded crane	22–83	November 17, 2022	Adult	Debilitated	+	-		
White-naped crane	22–84	November 18, 2022	Adult	Dead	+	+	A/white-naped crane/Kagoshima/KU-84/2022 (H5N1)	EPI_ISL_18472658
White-naped crane	22–85	November 18, 2022	Adult	Debilitated	+	+	A/white-naped crane/Kagoshima/KU-85/2022 (H5N1)	EPI_ISL_18472659
Hooded crane	22–86	November 19, 2022	Adult	Debilitated	+	+	A/hooded crane/Kagoshima/KU-86/2022 (H5N1)	EPI_ISL_18472584
White-naped crane	22–87	November 19, 2022	Adult	Debilitated	+	+	A/white-naped crane/Kagoshima/KU-87/2022 (H5N1)	EPI_ISL_18472585
Hooded crane	22–88	November 19, 2022	Adult	Debilitated	+	+	A/hooded crane/Kagoshima/KU-88/2022 (H5N1)	EPI_ISL_18472586

Host species	Sample ID	Collection date	Age	Vital status	RT-PCR	Virus isolation	Isolate	GISAID accession no.
Hooded crane	22–89	November 19, 2022	Adult	Dead	+	+	A/hooded crane/Kagoshima/KU-89/2022 (H5N1)	EPI_ISL_18472560
White-naped crane	22–91	November 20, 2022	Adult	Dead	+	+	A/white-naped crane/Kagoshima/KU-91/2022 (H5N1)	EPI_ISL_18472561
Hooded crane	22–92	November 20, 2022	Adult	Debilitated	+	-		
Hooded crane	22–93	November 20, 2022	Adult	Debilitated	+	-		
Hooded crane	22–94	November 21, 2022	Adult	Debilitated, and died within a day	+	+	A/hooded crane/Kagoshima/KU-94/2022 (H5N1)	EPI_ISL_18472562
White-naped crane	22–95	November 21, 2022	Adult	Dead	+	+	A/white-naped crane/Kagoshima/KU-95/2022 (H5N1)	EPI_ISL_18472563
Hooded crane	22–96	November 21, 2022	Adult	Dead	+	+	A/hooded crane/Kagoshima/KU-96/2022 (H5N1)	EPI_ISL_18472564
White-naped crane	22–97	November 21, 2022	Adult	Dead	+	+	A/white-naped crane/Kagoshima/KU-97/2022 (H5N1)	EPI_ISL_18472565
Hooded crane	22–104	November 21, 2022	Adult	Debilitated	+	-		
Hooded crane	22–105	November 21, 2022	Adult	Debilitated	+	+	A/hooded crane/Kagoshima/KU-105/2022 (H5N1)	EPI_ISL_18472666
Hooded crane	22–106	November 22, 2022	Adult	Dead	+	+	A/hooded crane/Kagoshima/KU-106/2022 (H5N1)	EPI_ISL_18472667
Hooded crane	22–107	November 22, 2022	Adult	Debilitated	+	-		
Hooded crane	22–108	November 22, 2022	Adult	Dead	+	+	A/hooded crane/Kagoshima/KU-108/2022 (H5N1)	EPI_ISL_18472668
White-naped crane	22–109	November 22, 2022	Adult	Dead	+	+	A/white-naped crane/Kagoshima/KU-109/2022 (H5N1)	EPI_ISL_18472669
Hooded crane	22–110	November 22, 2022	Adult	Debilitated	+	+	A/hooded crane/Kagoshima/KU-110/2022 (H5N1)	EPI_ISL_18472670
White-naped crane	22–111	November 22, 2022	Adult	Dead	+	+	A/white-naped crane/Kagoshima/KU-111/2022 (H5N1)	EPI_ISL_18472671
Hooded crane	22–112	November 22, 2022	Adult	Dead	+	+	A/hooded crane/Kagoshima/KU-112/2022 (H5N1)	EPI_ISL_18472672
White-naped crane	22–113	November 23, 2022	Adult	Dead	+	+	A/white-naped crane/Kagoshima/KU-113/2022 (H5N1)	EPI_ISL_18472673
Hooded crane	22–114	November 23, 2022	Adult	Debilitated	+	-		
Hooded crane	22–115	November 23, 2022	Adult	Debilitated	+	+	A/hooded crane/Kagoshima/KU-115/2022 (H5N1)	EPI_ISL_18472674
Hooded crane	22–116	November 23, 2022	Adult	Dead	+	+	A/hooded crane/Kagoshima/KU-116/2022 (H5N1)	EPI_ISL_18472675
Hooded crane	22–117	November 23, 2022	Adult	Debilitated	+	+	A/hooded crane/Kagoshima/KU-117/2022 (H5N1)	EPI_ISL_18472676
Hooded crane	22–118	November 24, 2022	Adult	Dead	+	+	A/hooded crane/Kagoshima/KU-118/2022 (H5N1)	EPI_ISL_18472677
White-naped crane	22–119	November 24, 2022	Juvenile	Dead	+	-		

Host species	Sample ID	Collection date	Age	Vital status	RT-PCR	Virus isolation	Isolate	GISAID accession no.
Hooded crane	22–120	November 24, 2022	Juvenile	Debilitated	+	+	A/hooded crane/Kagoshima/KU-120/2022 (H5N1)	EPI_ISL_18472678
Hooded crane	22–121	November 24, 2022	Juvenile	Dead	+	+	A/hooded crane/Kagoshima/KU-121/2022 (H5N1)	EPI_ISL_18472679
Hooded crane	22–122	November 24, 2022	Adult	Debilitated	+	+	A/hooded crane/Kagoshima/KU-122/2022 (H5N1)	EPI_ISL_18472680
Hooded crane	22–123	November 25, 2022	Adult	Debilitated, and died within a day	+	+	A/hooded crane/Kagoshima/KU-123/2022 (H5N1)	EPI_ISL_18472681
White-naped crane	22–124	November 25, 2022	Adult	Dead	+	+	A/white-naped crane/Kagoshima/KU-124/2022 (H5N1)	EPI_ISL_18472682
Hooded crane	22–125	November 25, 2022	Adult	Debilitated	+	-		
Hooded crane	22–126	November 25, 2022	Adult	Debilitated	+	-		
Hooded crane	22–127	November 26, 2022	Adult	Dead	+	+	A/hooded crane/Kagoshima/KU-127/2022 (H5N1)	EPI_ISL_18472683
Hooded crane	22–128	November 26, 2022	Adult	Dead	+	+	A/hooded crane/Kagoshima/KU-128/2022 (H5N1)	EPI_ISL_18472684
Hooded crane	22–133	November 26, 2022	Adult	Debilitated	+	+	A/hooded crane/Kagoshima/KU-133/2022 (H5N1)	EPI_ISL_18472685
Hooded crane	22–134	November 26, 2022	Adult	Dead	+	+	A/hooded crane/Kagoshima/KU-134/2022 (H5N1)	EPI_ISL_18472686
Hooded crane	22–135	November 27, 2022	Adult	Debilitated, and died within a day	+	+	A/hooded crane/Kagoshima/KU-135/2022 (H5N1)	EPI_ISL_18472687
White-naped crane	22–136	November 27, 2022	Adult	Debilitated, and died within a day	+	+	A/white-naped crane/Kagoshima/KU-136/2022 (H5N1)	EPI_ISL_18472688
Hooded crane	22–137	November 27, 2022	Adult	Dead	+	+	A/hooded crane/Kagoshima/KU-137/2022 (H5N1)	EPI_ISL_18472689
Hooded crane	22–138	November 27, 2022	Adult	Dead	+	+	A/hooded crane/Kagoshima/KU-138/2022 (H5N1)	EPI_ISL_18472690
Hooded crane	22–141	November 28, 2022	Adult	Dead	+	+	A/hooded crane/Kagoshima/KU-141/2022 (H5N1)	EPI_ISL_18472691
White-naped crane	22–142	November 28, 2022	Adult	Dead	+	+	A/white-naped crane/Kagoshima/KU-142/2022 (H5N1)	EPI_ISL_18472692
Hooded crane	22–143	November 28, 2022	Adult	Debilitated	+	+	A/hooded crane/Kagoshima/KU-143/2022 (H5N1)	EPI_ISL_18472693
Hooded crane	22–144	November 28, 2022	Adult	Dead	+	+	A/hooded crane/Kagoshima/KU-144/2022 (H5N1)	EPI_ISL_18472694
Hooded crane	22–145	November 28, 2022	Adult	Debilitated	+	+	A/hooded crane/Kagoshima/KU-145/2022 (H5N1)	EPI_ISL_18472695
White-naped crane	22–146	November 29, 2022	Adult	Dead	+	+	A/white-naped crane/Kagoshima/KU-146/2022 (H5N1)	EPI_ISL_18472696
Hooded crane	22–147	November 29, 2022	Adult	Debilitated, and died within a day	+	+	A/hooded crane/Kagoshima/KU-147/2022 (H5N1)	EPI_ISL_18472697
Hooded crane	22–148	November 29, 2022	Adult	Debilitated, and died within a day	+	+	A/hooded crane/Kagoshima/KU-148/2022 (H5N1)	EPI_ISL_18472698

Host species	Sample ID	Collection date	Age	Vital status	RT-PCR	Virus isolation	Isolate	GISAID accession no.
White-naped crane	22–149	November 27, 2022	Juvenile	Debilitated	+	-		
Hooded crane	22–150	November 28, 2022	Adult	Debilitated	+	-		
Hooded crane	22–153	November 30, 2022	Adult	Dead	+	+	A/hooded crane/Kagoshima/KU-153/2022 (H5N1)	EPI_ISL_18472699
Hooded crane	22–154	November 30, 2022	Adult	Dead	+	+	A/hooded crane/Kagoshima/KU-154/2022 (H5N1)	EPI_ISL_18472700
White-naped crane	22–155	November 30, 2022	Adult	Dead	+	+	A/white-naped crane/Kagoshima/KU-155/2022 (H5N1)	EPI_ISL_18472701
Hooded crane	22–163	December 1, 2022	Adult	Debilitated, and died within a day	+	+	A/hooded crane/Kagoshima/KU-163/2022 (H5N1)	EPI_ISL_18472702
Hooded crane	22–165	December 2, 2022	Adult	Dead	+	+	A/hooded crane/Kagoshima/KU-165/2022 (H5N1)	EPI_ISL_18472703
Hooded crane	22–166	December 2, 2022	Juvenile	Debilitated	+	-		
White-naped crane	22–167	December 1, 2022	Adult	Debilitated, and died within a day	+	+	A/white-naped crane/Kagoshima/KU-167/2022 (H5N1)	EPI_ISL_18472704
Hooded crane	22–169	December 3, 2022	Adult	Debilitated	+	-		
Hooded crane	22–170	December 3, 2022	Adult	Dead	+	+	A/hooded crane/Kagoshima/KU-170/2022 (H5N1)	EPI_ISL_18472705
White-naped crane	22–171	December 3, 2022	Adult	Debilitated	+	+	A/white-naped crane/Kagoshima/KU-171/2022 (H5N1)	EPI_ISL_18472706
Hooded crane	22–172	December 3, 2022	Adult	Debilitated	+	-		
Hooded crane	22–173	December 3, 2022	Adult	Dead	+	+	A/hooded crane/Kagoshima/KU-173/2022 (H5N1)	EPI_ISL_18472707
White-naped crane	22–176	December 4, 2022	Adult	Dead	+	+	A/white-naped crane/Kagoshima/KU-176/2022 (H5N1)	EPI_ISL_18472708
Hooded crane	22–177	December 4, 2022	Juvenile	Debilitated	+	-		
White-naped crane	22–178	December 4, 2022	Adult	Dead	+	+	A/white-naped crane/Kagoshima/KU-178/2022 (H5N1)	EPI_ISL_18472709
Hooded crane	22–179	December 5, 2022	Juvenile	Dead	+	-		
White-naped crane	22–180	December 5, 2022	Adult	Dead	+	+	A/white-naped crane/Kagoshima/KU-180/2022 (H5N1)	EPI_ISL_18472710
Hooded crane	22–181	December 6, 2022	Adult	Dead	+	+	A/hooded crane/Kagoshima/KU-181/2022 (H5N1)	EPI_ISL_18472711
Hooded crane	22–182	December 6, 2022	Adult	Debilitated	+	-		
White-naped crane	22–185	December 7, 2022	Adult	Dead	+	+	A/white-naped crane/Kagoshima/KU-185/2022 (H5N1)	EPI_ISL_18472587
Hooded crane	22–186	December 7, 2022	Adult	Dead	+	+	A/hooded crane/Kagoshima/KU-186/2022 (H5N1)	EPI_ISL_18472712
Hooded crane	22–189	December 8, 2022	Adult	Dead	+	+	A/hooded crane/Kagoshima/KU-189/2022 (H5N1)	EPI_ISL_18472713
Hooded crane	22–190	December 8, 2022	Adult	Dead	+	+	A/hooded crane/Kagoshima/KU-190/2022 (H5N1)	EPI_ISL_18472714

Host species	Sample ID	Collection date	Age	Vital status	RT-PCR	Virus isolation	Isolate	GISAID accession no.
White-naped crane	22–191	December 8, 2022	Adult	Dead	+	-		
Hooded crane	22–194	December 10, 2022	Adult	Debilitated, and died within a day	+	+	A/hooded crane/Kagoshima/KU-194/2022 (H5N1)	EPI_ISL_18472715
Hooded crane	22–195	December 10, 2022	Adult	Debilitated, and died within a day	+	+	A/hooded crane/Kagoshima/KU-195/2022 (H5N1)	EPI_ISL_18472716
Hooded crane	22–196	December 10, 2022	Adult	Debilitated	+	-		
Hooded crane	22–199	December 11, 2022	Adult	Dead	+	-		
Hooded crane	22–201	December 12, 2022	Adult	Dead	+	+	A/hooded crane/Kagoshima/KU-201/2022 (H5N1)	EPI_ISL_18472717
Hooded crane	22–205	December 13, 2022	Juvenile	Debilitated, and died within a day	+	-		
Hooded crane	22–206	December 13, 2022	Adult	Dead	+	-		
Hooded crane	22–212	December 15, 2022	Adult	Dead	+	+	A/hooded crane/Kagoshima/KU-212/2022 (H5N1)	EPI_ISL_18472718
White-naped crane	22–219	December 16, 2022	Unknown	Dead	+	-		
White-naped crane	22–222	December 16, 2022	Adult	Dead	+	-		
Hooded crane	22–223	December 18, 2022	Juvenile	Debilitated, and died within a day	+	-		
Hooded crane	22–226	December 17, 2022	Adult	Dead	+	-		
Hooded crane	22–233	December 23, 2022	Adult	Debilitated	+	-		
Hooded crane	22–255	January 9, 2023	Juvenile	Debilitated	+	-		
Hooded crane	22–257	January 9, 2023	Juvenile	Dead	+	-		
Hooded crane	22–267	January 22, 2023	Adult	Debilitated, and died within a day	+	+	A/hooded crane/Kagoshima/KU-267/2023 (H5N1)	EPI_ISL_18472588
Hooded crane	22–272	January 25, 2023	Adult	Debilitated	+	-		
Hooded crane	22–315	March 20, 2023	Juvenile	Dead	+	-		
Northern pintail	22–64	November 15, 2022	Adult	Dead	+	+	A/northern pintail/Kagoshima/KU-64/2022 (H5N1)	EPI_ISL_18508589
Mallard	22–131	November 27, 2022	Adult	Dead	+	+	A/mallard/Kagoshima/KU-131/2022 (H11N9)	EPI_ISL_18508591
Black kite	22–140	November 28, 2022	Adult	Dead	+	+	A/black kite/Kagoshima/KU-140/2022 (H5N1)	EPI_ISL_18508592

*AIV, avian influenza virus.

Appendix Table 3. AIVs isolated from crane roost water in study of highly pathogenic avian influenza A (H5N1) outbreak, Izumi Plain, Japan, 2022–23

Subtype	Isolate	Collection date	GISAID accession no.
H3N8	A/environment/Kagoshima/KU-J3/2022 (H3N8)	November 20, 2022	EPI_ISL_ 18472745
	A/environment/Kagoshima/KU-J4/2022 (H3N8)	November 20, 2022	EPI_ISL_ 18472746
	A/environment/Kagoshima/KU-J5/2022 (H3N8)	November 20, 2022	EPI_ISL_ 18472765
	A/environment/Kagoshima/KU-C3/2023 (H3N8)	January 24, 2023	EPI_ISL_ 18472751
	A/environment/Kagoshima/KU-G7/2023 (H3N8)	January 24, 2023	EPI_ISL_ 18472752
	A/environment/Kagoshima/KU-G8/2023 (H3N8)	January 24, 2023	EPI_ISL_ 18472753
	A/environment/Kagoshima/KU-C4/2023 (H3N8)	January 31, 2023	EPI_ISL_ 18472754
	A/environment/Kagoshima/KU-I1/2023 (H3N8)	January 31, 2023	EPI_ISL_ 18472755
H5N1	A/environment/Kagoshima/KU-6A/2022 (H5N1)	November 7, 2022	EPI_ISL_ 18472731
	A/environment/Kagoshima/KU-B1/2022 (H5N1)	November 7, 2022	EPI_ISL_ 18472732
	A/environment/Kagoshima/KU-B2/2022 (H5N1)	November 14, 2022	EPI_ISL_ 18472733
	A/environment/Kagoshima/KU-B3/2022 (H5N1)	November 14, 2022	EPI_ISL_ 18472734
	A/environment/Kagoshima/KU-B4/2022 (H5N1)	November 14, 2022	EPI_ISL_ 18472735
	A/environment/Kagoshima/KU-G1/2022 (H5N1)	November 14, 2022	EPI_ISL_ 18472736
	A/environment/Kagoshima/KU-G2/2022 (H5N1)	November 14, 2022	EPI_ISL_ 18472737
	A/environment/Kagoshima/KU-G3/2022 (H5N1)	November 14, 2022	EPI_ISL_ 18472738
	A/environment/Kagoshima/KU-B5/2022 (H5N1)	November 21, 2022	EPI_ISL_ 18472739
	A/environment/Kagoshima/KU-B6/2022 (H5N1)	November 21, 2022	EPI_ISL_ 18472740
	A/environment/Kagoshima/KU-B7/2022 (H5N1)	November 21, 2022	EPI_ISL_ 18472741
	A/environment/Kagoshima/KU-B8/2022 (H5N1)	November 21, 2022	EPI_ISL_ 18472756
	A/environment/Kagoshima/KU-D1/2022 (H5N1)	November 21, 2022	EPI_ISL_ 18472757
	A/environment/Kagoshima/KU-D2/2022 (H5N1)	November 21, 2022	EPI_ISL_ 18472758
	A/environment/Kagoshima/KU-D3/2022 (H5N1)	November 21, 2022	EPI_ISL_ 18472759
	A/environment/Kagoshima/KU-D4/2022 (H5N1)	November 21, 2022	EPI_ISL_ 18472760
	A/environment/Kagoshima/KU-G4/2022 (H5N1)	November 28, 2022	EPI_ISL_ 18472761
	A/environment/Kagoshima/KU-D6/2022 (H5N1)	December 6, 2022	EPI_ISL_ 18472762
	A/environment/Kagoshima/KU-G5/2022 (H5N1)	December 6, 2022	EPI_ISL_ 18472742
	A/environment/Kagoshima/KU-H1/2022 (H5N1)	December 6, 2022	EPI_ISL_ 18472743
	A/environment/Kagoshima/KU-B11/2022 (H5N1)	December 20, 2022	EPI_ISL_ 18472744
	A/environment/Kagoshima/KU-D7/2022 (H5N1)	December 20, 2022	EPI_ISL_ 18472764
	A/environment/Kagoshima/KU-J7/2022 (H5N1)	December 20, 2022	EPI_ISL_ 18472749
	A/environment/Kagoshima/KU-J8/2022 (H5N1)	December 27, 2022	EPI_ISL_ 18472750
H10N6	A/environment/Kagoshima/KU-B15/2022 (H10N6)	December 27, 2022	EPI_ISL_ 18472747
	A/environment/Kagoshima/KU-C2/2022 (H10N6)	December 27, 2022	EPI_ISL_ 18472748
	A/environment/Kagoshima/KU-H3/2022 (H10N6)	December 27, 2022	EPI_ISL_ 18472763
	A/environment/Kagoshima/KU-G9/2023 (H10N6)	February 7, 2023	EPI_ISL_ 18472766
	A/environment/Kagoshima/KU-H4/2023 (H10N6)	February 7, 2023	EPI_ISL_ 18472767
	A/environment/Kagoshima/KU-H5/2023 (H10N6)	February 7, 2023	EPI_ISL_ 18472768

Appendix Table 4. Crane sera tested in HI assays in study of highly pathogenic avian influenza A (H5N1) outbreak, Izumi Plain, Japan, 2022–23*.

Season	ID	Species	Collection date	Age	Vital status	RT-PCR
2021–22	21–8	Hooded crane	November 25, 2021	Adult	Dead	-
	21–11	Hooded crane	November 26, 2021	Juvenile	Dead	-
	21–14	Hooded crane	December 3, 2021	Adult	Dead	-
	21–15	Hooded crane	December 4, 2021	Juvenile	Debilitated	-
	21–21	Hooded crane	December 5, 2021	Juvenile	Debilitated, and died within a day	-
	21–19	Hooded crane	December 5, 2021	Adult	Dead	-
	21–20	Hooded crane	December 5, 2021	Juvenile	Dead	-
	21–10	Hooded crane	November 26, 2021	Adult	Debilitated, and died within a day	-
	21–36	White-naped crane	December 18, 2021	Juvenile	Debilitated	-
	21–38	Hooded crane	December 19, 2021	Juvenile	Debilitated, and died within a day	-
	21–51	Hooded crane	December 29, 2021	Juvenile	Dead	-
	21–52	Hooded crane	December 30, 2021	Adult	Dead	-
	21–53	Hooded crane	December 30, 2021	Juvenile	Dead	-
2022–23	22–105	Hooded crane	November 21, 2022	Adult	Debilitated	+ (H5N1)
	22–151	Hooded crane	November 24, 2022	Adult	Debilitated	+ (H5N1)
	22–152	Hooded crane	November 24, 2022	Adult	Debilitated	+ (H5N1)
	22–265	Hooded crane	January 20, 2023	Juvenile	Dead	-
	22–272	Hooded crane	January 25, 2023	Adult	Debilitated	+ (H5N1)
	22–278	Hooded crane	January 27, 2023	Juvenile	Debilitated, and died within a day	-
	22–280	Hooded crane	January 28, 2023	Juvenile	Dead	-
	22–281	Hooded crane	January 28, 2023	Adult	Debilitated	-
	22–282	White-naped crane	January 28, 2023	Adult	Dead	-
	22–283	White-naped crane	February 3, 2023	Adult	Dead	-

Season	ID	Species	Collection date	Age	Vital status	RT-PCR
	22–285	Hooded crane	February 8, 2023	Juvenile	Debilitated, and died within a day	-
	22–286	Hooded crane	February 11, 2023	Adult	Dead	-
	22–287	Hooded crane	February 11, 2023	Adult	Dead	-
	22–288	Hooded crane	February 12, 2023	Adult	Dead	-
	22–289	Hooded crane	January 25, 2023	Adult	Dead	-
	22–313	Hooded crane	March 19, 2023	Juvenile	Debilitated	-
	22–318	Hooded crane	March 26, 2023	Juvenile	Debilitated, and died within a day	-

*HI, hemagglutination inhibition.

Appendix Table 5. Genetic clades of viral gene segments from AIVs isolated in the Izumi Plain in study of highly pathogenic avian influenza A (H5N1) outbreak, 2022–23

Collection date	Subtype	Isolate	Genetic clade* of the following gene segment:								Genotype†
			HA	NA	PB2	PB1	PA	NP	M	NS	
November 1, 2022	H5N1	A/hooded crane/Kagoshima/KU-2/2022 (H5N1)	G2c	N1-I	PB2-I	PB1-I	PA-I	NP-I	M-I	NS-I	H5N1-I
November 2, 2022	H5N1	A/hooded crane/Kagoshima/KU-3/2022 (H5N1)	G2c	N1-I	PB2-I	PB1-I	PA-I	NP-I	M-I	NS-I	H5N1-I
November 3, 2022	H5N1	A/hooded crane/Kagoshima/KU-4/2022 (H5N1)	G2c	N1-I	PB2-I	PB1-I	PA-I	NP-I	M-I	NS-I	H5N1-I
November 3, 2022	H5N1	A/hooded crane/Kagoshima/KU-5/2022 (H5N1)	G2c	N1-I	PB2-I	PB1-I	PA-I	NP-I	M-I	NS-I	H5N1-I
November 4, 2022	H5N1	A/hooded crane/Kagoshima/KU-6/2022 (H5N1)	G2c	N1-I	PB2-I	PB1-I	PA-I	NP-I	M-I	NS-I	H5N1-I
November 4, 2022	H5N1	A/hooded crane/Kagoshima/KU-7/2022 (H5N1)	G2c	N1-I	PB2-I	PB1-I	PA-I	NP-I	M-I	NS-I	H5N1-I
November 4, 2022	H5N1	A/hooded crane/Kagoshima/KU-8/2022 (H5N1)	G2c	N1-I	PB2-I	PB1-I	PA-I	NP-I	M-I	NS-I	H5N1-I
November 4, 2022	H5N1	A/hooded crane/Kagoshima/KU-9/2022 (H5N1)	G2c	N1-I	PB2-I	PB1-I	PA-I	NP-I	M-I	NS-I	H5N1-I
November 5, 2022	H5N1	A/hooded crane/Kagoshima/KU-10/2022 (H5N1)	G2c	N1-I	PB2-I	PB1-I	PA-I	NP-I	M-I	NS-I	H5N1-I
November 5, 2022	H5N1	A/hooded crane/Kagoshima/KU-11/2022 (H5N1)	G2c	N1-I	PB2-II	PB1-II	PA-I	NP-I	M-I	NS-I	H5N1-II
November 5, 2022	H5N1	A/hooded crane/Kagoshima/KU-12/2022 (H5N1)	G2c	N1-I	PB2-I	PB1-I	PA-I	NP-II	M-I	NS-I	H5N1-III
November 6, 2022	H5N1	A/hooded crane/Kagoshima/KU-13/2022 (H5N1)	G2c	N1-I	PB2-I	PB1-I	PA-I	NP-I	M-I	NS-I	H5N1-I
November 6, 2022	H5N1	A/hooded crane/Kagoshima/KU-14/2022 (H5N1)	G2c	N1-I	PB2-I	PB1-I	PA-I	NP-I	M-I	NS-I	H5N1-I
November 6, 2022	H5N1	A/hooded crane/Kagoshima/KU-15/2022 (H5N1)	G2c	N1-I	PB2-I	PB1-I	PA-I	NP-I	M-I	NS-I	H5N1-I
November 6, 2022	H5N1	A/hooded crane/Kagoshima/KU-19/2022 (H5N1)	G2c	N1-I	PB2-II	PB1-II	PA-I	NP-I	M-I	NS-I	H5N1-II
November 6, 2022	H5N1	A/hooded crane/Kagoshima/KU-16/2022 (H5N1)	G2c	N1-I	PB2-I	PB1-III	PA-I	NP-II	M-I	NS-II	H5N1-IV
November 7, 2022	H5N1	A/hooded crane/Kagoshima/KU-20/2022 (H5N1)	G2c	N1-I	PB2-I	PB1-I	PA-I	NP-I	M-I	NS-I	H5N1-I
November 7, 2022	H5N1	A/hooded crane/Kagoshima/KU-22/2022 (H5N1)	G2c	N1-I	PB2-I	PB1-I	PA-I	NP-I	M-I	NS-I	H5N1-I
November 7, 2022	H5N1	A/hooded crane/Kagoshima/KU-23/2022 (H5N1)	G2c	N1-I	PB2-I	PB1-I	PA-I	NP-I	M-I	NS-I	H5N1-I
November 7, 2022	H5N1	A/hooded crane/Kagoshima/KU-24/2022 (H5N1)	G2c	N1-I	PB2-I	PB1-I	PA-I	NP-I	M-I	NS-I	H5N1-I
November 7, 2022	H5N1	A/environment/Kagoshima/KU-6A/2022 (H5N1)	G2c	N1-I	PB2-I	PB1-I	PA-I	NP-I	M-I	NS-I	H5N1-I
November 7, 2022	H5N1	A/environment/Kagoshima/KU-B1/2022 (H5N1)	G2c	N1-I	PB2-II	PB1-I	PA-IV	NP-I	M-I	NS-I	H5N1-V
November 8, 2022	H5N1	A/hooded crane/Kagoshima/KU-26/2022 (H5N1)	G2c	N1-I	PB2-I	PB1-I	PA-I	NP-I	M-I	NS-I	H5N1-I
November 8, 2022	H5N1	A/hooded crane/Kagoshima/KU-27/2022 (H5N1)	G2c	N1-I	PB2-I	PB1-I	PA-I	NP-I	M-I	NS-I	H5N1-I
November 8, 2022	H5N1	A/hooded crane/Kagoshima/KU-28/2022 (H5N1)	G2c	N1-I	PB2-I	PB1-I	PA-I	NP-I	M-I	NS-I	H5N1-I
November 8, 2022	H5N1	A/hooded crane/Kagoshima/KU-29/2022 (H5N1)	G2c	N1-I	PB2-I	PB1-I	PA-I	NP-I	M-I	NS-I	H5N1-I
November 8, 2022	H5N1	A/hooded crane/Kagoshima/KU-30/2022 (H5N1)	G2c	N1-I	PB2-I	PB1-I	PA-I	NP-I	M-I	NS-I	H5N1-I
November 8, 2022	H5N1	A/hooded crane/Kagoshima/KU-31/2022 (H5N1)	G2c	N1-I	PB2-I	PB1-I	PA-I	NP-I	M-I	NS-I	H5N1-I
November 8, 2022	H5N1	A/hooded crane/Kagoshima/KU-32/2022 (H5N1)	G2c	N1-I	PB2-I	PB1-I	PA-I	NP-I	M-I	NS-I	H5N1-I
November 9, 2022	H5N1	A/hooded crane/Kagoshima/KU-33/2022 (H5N1)	G2c	N1-I	PB2-I	PB1-I	PA-I	NP-I	M-I	NS-I	H5N1-I
November 9, 2022	H5N1	A/hooded crane/Kagoshima/KU-35/2022 (H5N1)	G2c	N1-I	PB2-I	PB1-I	PA-I	NP-I	M-I	NS-I	H5N1-I
November 9, 2022	H5N1	A/hooded crane/Kagoshima/KU-36/2022 (H5N1)	G2c	N1-I	PB2-I	PB1-I	PA-I	NP-I	M-I	NS-I	H5N1-I
November 9, 2022	H5N1	A/hooded crane/Kagoshima/KU-38/2022 (H5N1)	G2c	N1-I	PB2-III	PB1-I	PA-II	NP-I	M-I	NS-I	H5N1-VI
November 9, 2022	H5N1	A/hooded crane/Kagoshima/KU-39/2022 (H5N1)	G2c	N1-I	PB2-I	PB1-I	PA-I	NP-I	M-I	NS-I	H5N1-VII
November 10, 2022	H5N1	A/hooded crane/Kagoshima/KU-41/2022 (H5N1)	G2c	N1-I	PB2-I	PB1-I	PA-I	NP-I	M-I	NS-I	H5N1-I
November 10, 2022	H5N1	A/hooded crane/Kagoshima/KU-42/2022 (H5N1)	G2c	N1-I	PB2-I	PB1-I	PA-I	NP-I	M-I	NS-I	H5N1-I
November 10, 2022	H5N1	A/hooded crane/Kagoshima/KU-43/2022 (H5N1)	G2c	N1-I	PB2-I	PB1-I	PA-I	NP-I	M-I	NS-I	H5N1-I
November 10, 2022	H5N1	A/hooded crane/Kagoshima/KU-44/2022 (H5N1)	G2c	N1-I	PB2-I	PB1-I	PA-I	NP-I	M-I	NS-I	H5N1-I
November 10, 2022	H5N1	A/hooded crane/Kagoshima/KU-45/2022 (H5N1)	G2c	N1-I	PB2-I	PB1-I	PA-I	NP-I	M-I	NS-I	H5N1-I
November 10, 2022	H5N1	A/hooded crane/Kagoshima/KU-46/2022 (H5N1)	G2c	N1-I	PB2-I	PB1-I	PA-I	NP-I	M-I	NS-I	H5N1-I
November 10, 2022	H5N1	A/hooded crane/Kagoshima/KU-47/2022 (H5N1)	G2c	N1-I	PB2-I	PB1-I	PA-I	NP-I	M-I	NS-I	H5N1-I
November 10, 2022	H5N1	A/hooded crane/Kagoshima/KU-48/2022 (H5N1)	G2c	N1-I	PB2-I	PB1-I	PA-I	NP-I	M-I	NS-I	H5N1-I
November 10, 2022	H5N1	A/hooded crane/Kagoshima/KU-50/2022 (H5N1)	G2c	N1-I	PB2-I	PB1-I	PA-I	NP-I	M-I	NS-I	H5N1-I
November 10, 2022	H5N1	A/hooded crane/Kagoshima/KU-40/2022 (H5N1)	G2b	N1-II	PB2-IV	PB1-IV	PA-II	NP-III	M-II	NS-II	H5N1-VIII
November 11, 2022	H5N1	A/hooded crane/Kagoshima/KU-51/2022 (H5N1)	G2c	N1-I	PB2-I	PB1-I	PA-I	NP-I	M-I	NS-I	H5N1-I
November 11, 2022	H5N1	A/hooded crane/Kagoshima/KU-52/2022 (H5N1)	G2c	N1-I	PB2-I	PB1-I	PA-I	NP-I	M-I	NS-I	H5N1-I
November 11, 2022	H5N1	A/hooded crane/Kagoshima/KU-53/2022 (H5N1)	G2c	N1-I	PB2-I	PB1-I	PA-I	NP-I	M-I	NS-I	H5N1-I
November 12, 2022	H5N1	A/hooded crane/Kagoshima/KU-54/2022 (H5N1)	G2c	N1-I	PB2-I	PB1-I	PA-I	NP-I	M-I	NS-I	H5N1-I
November 12, 2022	H5N1	A/hooded crane/Kagoshima/KU-55/2022 (H5N1)	G2c	N1-I	PB2-I	PB1-I	PA-I	NP-I	M-I	NS-I	H5N1-I
November 12, 2022	H5N1	A/hooded crane/Kagoshima/KU-56/2022 (H5N1)	G2c	N1-I	PB2-I	PB1-I	PA-I	NP-I	M-I	NS-I	H5N1-I

Collection date	Subtype	Isolate	Genetic clade* of the following gene segment: HA	NA	PB2	PB1	PA	NP	M	NS	Genotype†
November 12, 2022	H5N1	A/hooded crane/Kagoshima/KU-57/2022 (H5N1)	G2c	N1-I	PB2-I	PB1-I	PA-I	NP-I	M-I	NS-I	H5N1-I
November 13, 2022	H5N1	A/hooded crane/Kagoshima/KU-58/2022 (H5N1)	G2c	N1-I	PB2-I	PB1-I	PA-I	NP-I	M-I	NS-I	H5N1-I
November 13, 2022	H5N1	A/hooded crane/Kagoshima/KU-59/2022 (H5N1)	G2c	N1-I	PB2-I	PB1-I	PA-I	NP-I	M-I	NS-I	H5N1-I
November 14, 2022	H5N1	A/hooded crane/Kagoshima/KU-60/2022 (H5N1)	G2c	N1-I	PB2-I	PB1-I	PA-I	NP-I	M-I	NS-I	H5N1-I
November 14, 2022	H5N1	A/hooded crane/Kagoshima/KU-61/2022 (H5N1)	G2c	N1-I	PB2-I	PB1-I	PA-I	NP-I	M-I	NS-I	H5N1-I
November 14, 2022	H5N1	A/white-naped crane/Kagoshima/KU-62/2022 (H5N1)	G2c	N1-I	PB2-I	PB1-I	PA-I	NP-I	M-I	NS-I	H5N1-I
November 14, 2022	H5N1	A/hooded crane/Kagoshima/KU-63/2022 (H5N1)	G2c	N1-I	PB2-I	PB1-I	PA-I	NP-I	M-I	NS-I	H5N1-I
November 14, 2022	H5N1	A/hooded crane/Kagoshima/KU-65/2022 (H5N1)	G2c	N1-I	PB2-I	PB1-I	PA-I	NP-I	M-I	NS-I	H5N1-I
November 14, 2022	H5N1	A/white-naped crane/Kagoshima/KU-66/2022 (H5N1)	G2c	N1-I	PB2-I	PB1-I	PA-I	NP-I	M-I	NS-I	H5N1-I
November 14, 2022	H5N1	A/environment/Kagoshima/KU-G1/2022 (H5N1)	G2c	N1-I	PB2-I	PB1-I	PA-I	NP-I	M-I	NS-I	H5N1-I
November 14, 2022	H5N1	A/environment/Kagoshima/KU-G2/2022 (H5N1)	G2c	N1-I	PB2-I	PB1-I	PA-I	NP-I	M-I	NS-I	H5N1-I
November 14, 2022	H5N1	A/environment/Kagoshima/KU-G3/2022 (H5N1)	G2c	N1-I	PB2-I	PB1-I	PA-I	NP-I	M-I	NS-I	H5N1-I
November 14, 2022	H5N1	A/environment/Kagoshima/KU-B3/2022 (H5N1)	G2c	N1-I	PB2-I	PB1-III	PA-III	NP-II	M-I	NS-III	H5N1-IX
November 14, 2022	H5N1	A/environment/Kagoshima/KU-B2/2022 (H5N1)	G2c	N1-I	PB2-III	PB1-III	PA-III	NP-II	M-I	NS-I	H5N1-X
November 14, 2022	H5N1	A/environment/Kagoshima/KU-B4/2022 (H5N1)	G2c	N1-I	PB2-I	PB1-III	PA-III	NP-II	M-I	NS-I	H5N1-XI
November 15, 2022	H5N1	A/hooded crane/Kagoshima/KU-67/2022 (H5N1)	G2c	N1-I	PB2-I	PB1-I	PA-I	NP-I	M-I	NS-I	H5N1-I
November 15, 2022	H5N1	A/hooded crane/Kagoshima/KU-68/2022 (H5N1)	G2c	N1-I	PB2-I	PB1-I	PA-I	NP-I	M-I	NS-I	H5N1-I
November 15, 2022	H5N1	A/hooded crane/Kagoshima/KU-69/2022 (H5N1)	G2c	N1-I	PB2-I	PB1-I	PA-I	NP-I	M-I	NS-I	H5N1-I
November 15, 2022	H5N1	A/hooded crane/Kagoshima/KU-71/2022 (H5N1)	G2c	N1-I	PB2-I	PB1-I	PA-I	NP-I	M-I	NS-I	H5N1-I
November 15, 2022	H5N1	A/northern pintail/Kagoshima/KU-64/2022 (H5N1)	G2c	N1-I	PB2-I	PB1-III	PA-III	NP-II	M-I	NS-III	H5N1-XII
November 16, 2022	H5N1	A/hooded crane/Kagoshima/KU-73/2022 (H5N1)	G2c	N1-I	PB2-I	PB1-I	PA-I	NP-I	M-I	NS-I	H5N1-I
November 16, 2022	H5N1	A/hooded crane/Kagoshima/KU-74/2022 (H5N1)	G2c	N1-I	PB2-I	PB1-I	PA-I	NP-I	M-I	NS-I	H5N1-I
November 16, 2022	H5N1	A/hooded crane/Kagoshima/KU-75/2022 (H5N1)	G2c	N1-I	PB2-I	PB1-I	PA-I	NP-I	M-I	NS-I	H5N1-I
November 16, 2022	H5N1	A/hooded crane/Kagoshima/KU-76/2022 (H5N1)	G2c	N1-I	PB2-I	PB1-I	PA-I	NP-I	M-I	NS-I	H5N1-I
November 17, 2022	H5N1	A/hooded crane/Kagoshima/KU-77/2022 (H5N1)	G2c	N1-I	PB2-I	PB1-I	PA-I	NP-I	M-I	NS-I	H5N1-I
November 17, 2022	H5N1	A/white-naped crane/Kagoshima/KU-78/2022 (H5N1)	G2c	N1-I	PB2-I	PB1-I	PA-I	NP-I	M-I	NS-I	H5N1-I
November 17, 2022	H5N1	A/hooded crane/Kagoshima/KU-79/2022 (H5N1)	G2c	N1-I	PB2-I	PB1-I	PA-I	NP-I	M-I	NS-I	H5N1-I
November 17, 2022	H5N1	A/hooded crane/Kagoshima/KU-80/2022 (H5N1)	G2c	N1-I	PB2-I	PB1-I	PA-I	NP-I	M-I	NS-I	H5N1-I
November 17, 2022	H5N1	A/white-naped crane/Kagoshima/KU-81/2022 (H5N1)	G2c	N1-I	PB2-I	PB1-I	PA-I	NP-I	M-I	NS-I	H5N1-I
November 18, 2022	H5N1	A/white-naped crane/Kagoshima/KU-84/2022 (H5N1)	G2c	N1-I	PB2-I	PB1-I	PA-I	NP-I	M-I	NS-I	H5N1-I
November 18, 2022	H5N1	A/white-naped crane/Kagoshima/KU-85/2022 (H5N1)	G2c	N1-I	PB2-I	PB1-I	PA-I	NP-I	M-I	NS-I	H5N1-I
November 19, 2022	H5N1	A/hooded crane/Kagoshima/KU-86/2022 (H5N1)	G2c	N1-I	PB2-I	PB1-I	PA-I	NP-I	M-I	NS-I	H5N1-I
November 19, 2022	H5N1	A/white-naped crane/Kagoshima/KU-87/2022 (H5N1)	G2c	N1-I	PB2-I	PB1-I	PA-I	NP-I	M-I	NS-I	H5N1-I
November 19, 2022	H5N1	A/hooded crane/Kagoshima/KU-88/2022 (H5N1)	G2c	N1-I	PB2-I	PB1-I	PA-I	NP-I	M-I	NS-I	H5N1-I
November 19, 2022	H5N1	A/hooded crane/Kagoshima/KU-89/2022 (H5N1)	G2c	N1-I	PB2-I	PB1-I	PA-I	NP-I	M-I	NS-I	H5N1-I
November 20, 2022	H5N1	A/white-naped crane/Kagoshima/KU-91/2022 (H5N1)	G2c	N1-I	PB2-I	PB1-I	PA-I	NP-I	M-I	NS-I	H5N1-I
November 21, 2022	H5N1	A/hooded crane/Kagoshima/KU-94/2022 (H5N1)	G2c	N1-I	PB2-I	PB1-I	PA-I	NP-I	M-I	NS-I	H5N1-I
November 21, 2022	H5N1	A/white-naped crane/Kagoshima/KU-95/2022 (H5N1)	G2c	N1-I	PB2-I	PB1-I	PA-I	NP-I	M-I	NS-I	H5N1-I
November 21, 2022	H5N1	A/hooded crane/Kagoshima/KU-96/2022 (H5N1)	G2c	N1-I	PB2-I	PB1-I	PA-I	NP-I	M-I	NS-I	H5N1-I
November 21, 2022	H5N1	A/white-naped crane/Kagoshima/KU-97/2022 (H5N1)	G2c	N1-I	PB2-I	PB1-I	PA-I	NP-I	M-I	NS-I	H5N1-I
November 21, 2022	H5N1	A/hooded crane/Kagoshima/KU-105/2022 (H5N1)	G2c	N1-I	PB2-I	PB1-I	PA-I	NP-I	M-I	NS-I	H5N1-I
November 21, 2022	H5N1	A/environment/Kagoshima/KU-B5/2022 (H5N1)	G2c	N1-I	PB2-I	PB1-I	PA-I	NP-I	M-I	NS-I	H5N1-I
November 21, 2022	H5N1	A/environment/Kagoshima/KU-B6/2022 (H5N1)	G2c	N1-I	PB2-I	PB1-I	PA-I	NP-I	M-I	NS-I	H5N1-I
November 21, 2022	H5N1	A/environment/Kagoshima/KU-B7/2022 (H5N1)	G2c	N1-I	PB2-I	PB1-I	PA-I	NP-I	M-I	NS-I	H5N1-I
November 21, 2022	H5N1	A/environment/Kagoshima/KU-B8/2022 (H5N1)	G2c	N1-I	PB2-I	PB1-I	PA-I	NP-I	M-I	NS-I	H5N1-I
November 21, 2022	H5N1	A/environment/Kagoshima/KU-D1/2022 (H5N1)	G2c	N1-I	PB2-I	PB1-I	PA-I	NP-I	M-I	NS-I	H5N1-I
November 21, 2022	H5N1	A/environment/Kagoshima/KU-D2/2022 (H5N1)	G2c	N1-I	PB2-I	PB1-I	PA-I	NP-I	M-I	NS-I	H5N1-I
November 21, 2022	H5N1	A/environment/Kagoshima/KU-D3/2022 (H5N1)	G2c	N1-I	PB2-I	PB1-I	PA-I	NP-I	M-I	NS-I	H5N1-I
November 21, 2022	H5N1	A/environment/Kagoshima/KU-D4/2022 (H5N1)	G2c	N1-I	PB2-I	PB1-I	PA-I	NP-I	M-I	NS-I	H5N1-I
November 22, 2022	H5N1	A/hooded crane/Kagoshima/KU-106/2022 (H5N1)	G2c	N1-I	PB2-I	PB1-I	PA-I	NP-I	M-I	NS-I	H5N1-I
November 22, 2022	H5N1	A/hooded crane/Kagoshima/KU-108/2022 (H5N1)	G2c	N1-I	PB2-I	PB1-I	PA-I	NP-I	M-I	NS-I	H5N1-I

Collection date	Subtype	Isolate	Genetic clade* of the following gene segment: HA	NA	PB2	PB1	PA	NP	M	NS	Genotype†
November 22, 2022	H5N1	A/white-naped crane/Kagoshima/KU-109/2022 (H5N1)	G2c	N1-I	PB2-I	PB1-I	PA-I	NP-I	M-I	NS-I	H5N1-I
November 22, 2022	H5N1	A/hooded crane/Kagoshima/KU-110/2022 (H5N1)	G2c	N1-I	PB2-I	PB1-I	PA-I	NP-I	M-I	NS-I	H5N1-I
November 22, 2022	H5N1	A/white-naped crane/Kagoshima/KU-111/2022 (H5N1)	G2c	N1-I	PB2-I	PB1-I	PA-I	NP-I	M-I	NS-I	H5N1-I
November 22, 2022	H5N1	A/hooded crane/Kagoshima/KU-112/2022 (H5N1)	G2c	N1-I	PB2-I	PB1-I	PA-I	NP-I	M-I	NS-I	H5N1-I
November 23, 2022	H5N1	A/white-naped crane/Kagoshima/KU-113/2022 (H5N1)	G2c	N1-I	PB2-I	PB1-I	PA-I	NP-I	M-I	NS-I	H5N1-I
November 23, 2022	H5N1	A/hooded crane/Kagoshima/KU-115/2022 (H5N1)	G2c	N1-I	PB2-I	PB1-I	PA-I	NP-I	M-I	NS-I	H5N1-I
November 23, 2022	H5N1	A/hooded crane/Kagoshima/KU-116/2022 (H5N1)	G2c	N1-I	PB2-I	PB1-I	PA-I	NP-I	M-I	NS-I	H5N1-I
November 23, 2022	H5N1	A/hooded crane/Kagoshima/KU-117/2022 (H5N1)	G2c	N1-I	PB2-I	PB1-I	PA-I	NP-I	M-I	NS-I	H5N1-I
November 24, 2022	H5N1	A/hooded crane/Kagoshima/KU-118/2022 (H5N1)	G2c	N1-I	PB2-I	PB1-I	PA-I	NP-I	M-I	NS-I	H5N1-I
November 24, 2022	H5N1	A/hooded crane/Kagoshima/KU-120/2022 (H5N1)	G2c	N1-I	PB2-I	PB1-I	PA-I	NP-I	M-I	NS-I	H5N1-I
November 24, 2022	H5N1	A/hooded crane/Kagoshima/KU-121/2022 (H5N1)	G2c	N1-I	PB2-I	PB1-I	PA-I	NP-I	M-I	NS-I	H5N1-I
November 24, 2022	H5N1	A/hooded crane/Kagoshima/KU-122/2022 (H5N1)	G2c	N1-I	PB2-I	PB1-I	PA-I	NP-I	M-I	NS-I	H5N1-I
November 25, 2022	H5N1	A/hooded crane/Kagoshima/KU-123/2022 (H5N1)	G2c	N1-I	PB2-I	PB1-I	PA-I	NP-I	M-I	NS-I	H5N1-I
November 25, 2022	H5N1	A/white-naped crane/Kagoshima/KU-124/2022 (H5N1)	G2c	N1-I	PB2-I	PB1-I	PA-I	NP-I	M-I	NS-I	H5N1-I
November 26, 2022	H5N1	A/hooded crane/Kagoshima/KU-127/2022 (H5N1)	G2c	N1-I	PB2-I	PB1-I	PA-I	NP-I	M-I	NS-I	H5N1-I
November 26, 2022	H5N1	A/hooded crane/Kagoshima/KU-128/2022 (H5N1)	G2c	N1-I	PB2-I	PB1-I	PA-I	NP-I	M-I	NS-I	H5N1-I
November 26, 2022	H5N1	A/hooded crane/Kagoshima/KU-133/2022 (H5N1)	G2c	N1-I	PB2-I	PB1-I	PA-I	NP-I	M-I	NS-I	H5N1-I
November 26, 2022	H5N1	A/hooded crane/Kagoshima/KU-134/2022 (H5N1)	G2c	N1-I	PB2-I	PB1-I	PA-I	NP-I	M-I	NS-I	H5N1-I
November 27, 2022	H5N1	A/hooded crane/Kagoshima/KU-135/2022 (H5N1)	G2c	N1-I	PB2-I	PB1-I	PA-I	NP-I	M-I	NS-I	H5N1-I
November 27, 2022	H5N1	A/white-naped crane/Kagoshima/KU-136/2022 (H5N1)	G2c	N1-I	PB2-I	PB1-I	PA-I	NP-I	M-I	NS-I	H5N1-I
November 27, 2022	H5N1	A/hooded crane/Kagoshima/KU-137/2022 (H5N1)	G2c	N1-I	PB2-I	PB1-I	PA-I	NP-I	M-I	NS-I	H5N1-I
November 27, 2022	H5N1	A/hooded crane/Kagoshima/KU-138/2022 (H5N1)	G2c	N1-I	PB2-I	PB1-I	PA-I	NP-I	M-I	NS-I	H5N1-I
November 28, 2022	H5N1	A/black kite/Kagoshima/KU-140/2022 (H5N1)	G2c	N1-I	PB2-I	PB1-I	PA-I	NP-I	M-I	NS-I	H5N1-I
November 28, 2022	H5N1	A/hooded crane/Kagoshima/KU-141/2022 (H5N1)	G2c	N1-I	PB2-I	PB1-I	PA-I	NP-I	M-I	NS-I	H5N1-I
November 28, 2022	H5N1	A/white-naped crane/Kagoshima/KU-142/2022 (H5N1)	G2c	N1-I	PB2-I	PB1-I	PA-I	NP-I	M-I	NS-I	H5N1-I
November 28, 2022	H5N1	A/hooded crane/Kagoshima/KU-143/2022 (H5N1)	G2c	N1-I	PB2-I	PB1-I	PA-I	NP-I	M-I	NS-I	H5N1-I
November 28, 2022	H5N1	A/hooded crane/Kagoshima/KU-144/2022 (H5N1)	G2c	N1-I	PB2-I	PB1-I	PA-I	NP-I	M-I	NS-I	H5N1-I
November 28, 2022	H5N1	A/hooded crane/Kagoshima/KU-145/2022 (H5N1)	G2c	N1-I	PB2-I	PB1-I	PA-I	NP-I	M-I	NS-I	H5N1-I
November 28, 2022	H5N1	A/environment/Kagoshima/KU-G4/2022 (H5N1)	G2c	N1-I	PB2-I	PB1-I	PA-I	NP-I	M-I	NS-I	H5N1-I
November 29, 2022	H5N1	A/white-naped crane/Kagoshima/KU-146/2022 (H5N1)	G2c	N1-I	PB2-I	PB1-I	PA-I	NP-I	M-I	NS-I	H5N1-I
November 29, 2022	H5N1	A/hooded crane/Kagoshima/KU-147/2022 (H5N1)	G2c	N1-I	PB2-I	PB1-I	PA-I	NP-I	M-I	NS-I	H5N1-I
November 29, 2022	H5N1	A/hooded crane/Kagoshima/KU-148/2022 (H5N1)	G2c	N1-I	PB2-I	PB1-I	PA-I	NP-I	M-I	NS-I	H5N1-I
November 30, 2022	H5N1	A/hooded crane/Kagoshima/KU-153/2022 (H5N1)	G2c	N1-I	PB2-I	PB1-I	PA-I	NP-I	M-I	NS-I	H5N1-I
November 30, 2022	H5N1	A/hooded crane/Kagoshima/KU-154/2022 (H5N1)	G2c	N1-I	PB2-I	PB1-I	PA-I	NP-I	M-I	NS-I	H5N1-I
November 30, 2022	H5N1	A/white-naped crane/Kagoshima/KU-155/2022 (H5N1)	G2c	N1-I	PB2-I	PB1-I	PA-I	NP-I	M-I	NS-I	H5N1-I
December 1, 2022	H5N1	A/hooded crane/Kagoshima/KU-163/2022 (H5N1)	G2c	N1-I	PB2-I	PB1-I	PA-I	NP-I	M-I	NS-I	H5N1-I
December 1, 2022	H5N1	A/white-naped crane/Kagoshima/KU-167/2022 (H5N1)	G2c	N1-I	PB2-I	PB1-I	PA-I	NP-I	M-I	NS-I	H5N1-I
December 2, 2022	H5N1	A/hooded crane/Kagoshima/KU-165/2022 (H5N1)	G2c	N1-I	PB2-I	PB1-I	PA-I	NP-I	M-I	NS-I	H5N1-I
December 3, 2022	H5N1	A/hooded crane/Kagoshima/KU-170/2022 (H5N1)	G2c	N1-I	PB2-I	PB1-I	PA-I	NP-I	M-I	NS-I	H5N1-I
December 3, 2022	H5N1	A/white-naped crane/Kagoshima/KU-171/2022 (H5N1)	G2c	N1-I	PB2-I	PB1-I	PA-I	NP-I	M-I	NS-I	H5N1-I
December 3, 2022	H5N1	A/hooded crane/Kagoshima/KU-173/2022 (H5N1)	G2c	N1-I	PB2-I	PB1-I	PA-I	NP-I	M-I	NS-I	H5N1-I

			Genetic clade* of the following gene segment:								
Collection date	Subtype	Isolate	HA	NA	PB2	PB1	PA	NP	M	NS	Genotype†
December 4, 2022	H5N1	A/white-naped crane/Kagoshima/KU-176/2022 (H5N1)	G2c	N1-I	PB2-I	PB1-I	PA-I	NP-I	M-I	NS-I	H5N1-I
December 4, 2022	H5N1	A/white-naped crane/Kagoshima/KU-178/2022 (H5N1)	G2c	N1-I	PB2-I	PB1-I	PA-I	NP-I	M-I	NS-I	H5N1-I
December 5, 2022	H5N1	A/white-naped crane/Kagoshima/KU-180/2022 (H5N1)	G2c	N1-I	PB2-I	PB1-I	PA-I	NP-I	M-I	NS-I	H5N1-I
December 6, 2022	H5N1	A/hooded crane/Kagoshima/KU-181/2022 (H5N1)	G2c	N1-I	PB2-I	PB1-I	PA-I	NP-I	M-I	NS-I	H5N1-I
December 6, 2022	H5N1	A/environment/Kagoshima/KU-D6/2022 (H5N1)	G2c	N1-I	PB2-I	PB1-I	PA-I	NP-I	M-I	NS-I	H5N1-I
December 6, 2022	H5N1	A/environment/Kagoshima/KU-G5/2022 (H5N1)	G2c	N1-I	PB2-I	PB1-I	PA-I	NP-I	M-I	NS-I	H5N1-I
December 6, 2022	H5N1	A/environment/Kagoshima/KU-H1/2022 (H5N1)	G2c	N1-I	PB2-I	PB1-I	PA-I	NP-I	M-I	NS-I	H5N1-I
December 7, 2022	H5N1	A/white-naped crane/Kagoshima/KU-185/2022 (H5N1)	G2c	N1-I	PB2-I	PB1-I	PA-I	NP-I	M-I	NS-I	H5N1-I
December 7, 2022	H5N1	A/hooded crane/Kagoshima/KU-186/2022 (H5N1)	G2c	N1-I	PB2-I	PB1-I	PA-I	NP-I	M-I	NS-I	H5N1-I
December 8, 2022	H5N1	A/hooded crane/Kagoshima/KU-189/2022 (H5N1)	G2c	N1-I	PB2-I	PB1-I	PA-I	NP-I	M-I	NS-I	H5N1-I
December 8, 2022	H5N1	A/hooded crane/Kagoshima/KU-190/2022 (H5N1)	G2c	N1-I	PB2-I	PB1-I	PA-I	NP-I	M-I	NS-I	H5N1-I
December 10, 2022	H5N1	A/hooded crane/Kagoshima/KU-194/2022 (H5N1)	G2c	N1-I	PB2-I	PB1-I	PA-I	NP-I	M-I	NS-I	H5N1-I
December 10, 2022	H5N1	A/hooded crane/Kagoshima/KU-195/2022 (H5N1)	G2c	N1-I	PB2-I	PB1-I	PA-I	NP-I	M-I	NS-I	H5N1-I
December 12, 2022	H5N1	A/hooded crane/Kagoshima/KU-201/2022 (H5N1)	G2c	N1-I	PB2-I	PB1-I	PA-I	NP-I	M-I	NS-I	H5N1-I
December 15, 2022	H5N1	A/hooded crane/Kagoshima/KU-212/2022 (H5N1)	G2c	N1-I	PB2-I	PB1-I	PA-I	NP-I	M-I	NS-I	H5N1-I
December 20, 2022	H5N1	A/environment/Kagoshima/KU-B11/2022 (H5N1)	G2c	N1-I	PB2-I	PB1-I	PA-I	NP-I	M-I	NS-I	H5N1-I
December 20, 2022	H5N1	A/environment/Kagoshima/KU-D7/2022 (H5N1)	G2c	N1-I	PB2-I	PB1-I	PA-I	NP-I	M-I	NS-I	H5N1-I
December 27, 2022	H5N1	A/environment/Kagoshima/KU-J7/2022 (H5N1)	G2c	N1-I	PB2-II	PB1-III	PA-V	NP-II	M-I	NS-III	H5N1-XIII
December 27, 2022	H5N1	A/environment/Kagoshima/KU-J8/2022 (H5N1)	G2c	N1-I	PB2-II	PB1-III	PA-V	NP-II	M-I	NS-III	H5N1-XIII
January 22, 2023	H5N1	A/hooded crane/Kagoshima/KU-267/2023 (H5N1)	G2c	N1-I	PB2-I	PB1-III	PA-III	NP-II	M-I	NS-III	H5N1-XII
November 20, 2022	H3N8	A/environment/Kagoshima/KU-J3/2022 (H3N8)	H3-I	N8-I	PB2-V	PB1-V	PA-V	NP-IV	M-III	NS-IV	H3N8-I
November 20, 2022	H3N8	A/environment/Kagoshima/KU-J5/2022 (H3N8)	H3-I	N8-I	PB2-V	PB1-V	PA-V	NP-IV	M-III	NS-IV	H3N8-I
November 20, 2022	H3N8	A/environment/Kagoshima/KU-J4/2022 (H3N8)	H3-I	N8-I	PB2-V	PB1-V	PA-V	NP-IV	M-IV	NS-IV	H3N8-II
January 24, 2023	H3N8	A/environment/Kagoshima/KU-C3/2023 (H3N8)	H3-II	N8-II	PB2-V	PB1-V	PA-V	NP-IV	M-IV	NS-I	H3N8-III
January 24, 2023	H3N8	A/environment/Kagoshima/KU-G7/2023 (H3N8)	H3-II	N8-II	PB2-V	PB1-V	PA-V	NP-IV	M-I	NS-I	H3N8-IV
January 24, 2023	H3N8	A/environment/Kagoshima/KU-G8/2023 (H3N8)	H3-II	N8-II	PB2-V	PB1-V	PA-V	NP-IV	M-I	NS-I	H3N8-IV
January 31, 2023	H3N8	A/environment/Kagoshima/KU-C4/2023 (H3N8)	H3-II	N8-II	PB2-V	PB1-V	PA-V	NP-IV	M-IV	NS-I	H3N8-III
January 31, 2023	H3N8	A/environment/Kagoshima/KU-I1/2023 (H3N8)	H3-II	N8-II	PB2-V	PB1-V	PA-V	NP-IV	M-I	NS-I	H3N8-IV
November 27, 2022	H11N9	A/mallard/Kagoshima/KU-131/2022 (H11N9)	—***	—	PB2-I	PB1-VI	PA-VI	NP-V	M-IV	NS-II	—
December 27, 2022	H10N6	A/environment/Kagoshima/KU-B15/2022 (H10N6)	—	N6-I	PB2-VI	PB1-VII	PA-VII	NP-VI	M-V	NS-V	H10N6-I
December 27, 2022	H10N6	A/environment/Kagoshima/KU-C2/2022 (H10N6)	—	N6-I	PB2-VI	PB1-VII	PA-VII	NP-VI	M-V	NS-V	H10N6-I
December 27, 2022	H10N6	A/environment/Kagoshima/KU-H3/2022 (H10N6)	—	N6-I	PB2-VI	PB1-VII	PA-VII	NP-VI	M-V	NS-V	H10N6-I
February 9, 2023	H10N6	A/environment/Kagoshima/KU-G9/2023 (H10N6)	—	N6-II	PB2-VI	PB1-VII	PA-VII	NP-VI	M-V	NS-VI	H10N6-II
February 9, 2023	H10N6	A/environment/Kagoshima/KU-H4/2023 (H10N6)	—	N6-II	PB2-VI	PB1-VII	PA-VII	NP-VI	M-V	NS-VI	H10N6-II
February 9, 2023	H10N6	A/environment/Kagoshima/KU-H5/2023 (H10N6)	—	N6-II	PB2-VI	PB1-VII	PA-VII	NP-VI	M-V	NS-VI	H10N6-II

*Genetic clades for each viral gene segment were defined based on the phylogenetic trees (Figures 2, 4, 5, Appendix Figures 2 and 3).
†Genotypes were defined based on genetic constellations.
‡—, not applicable.

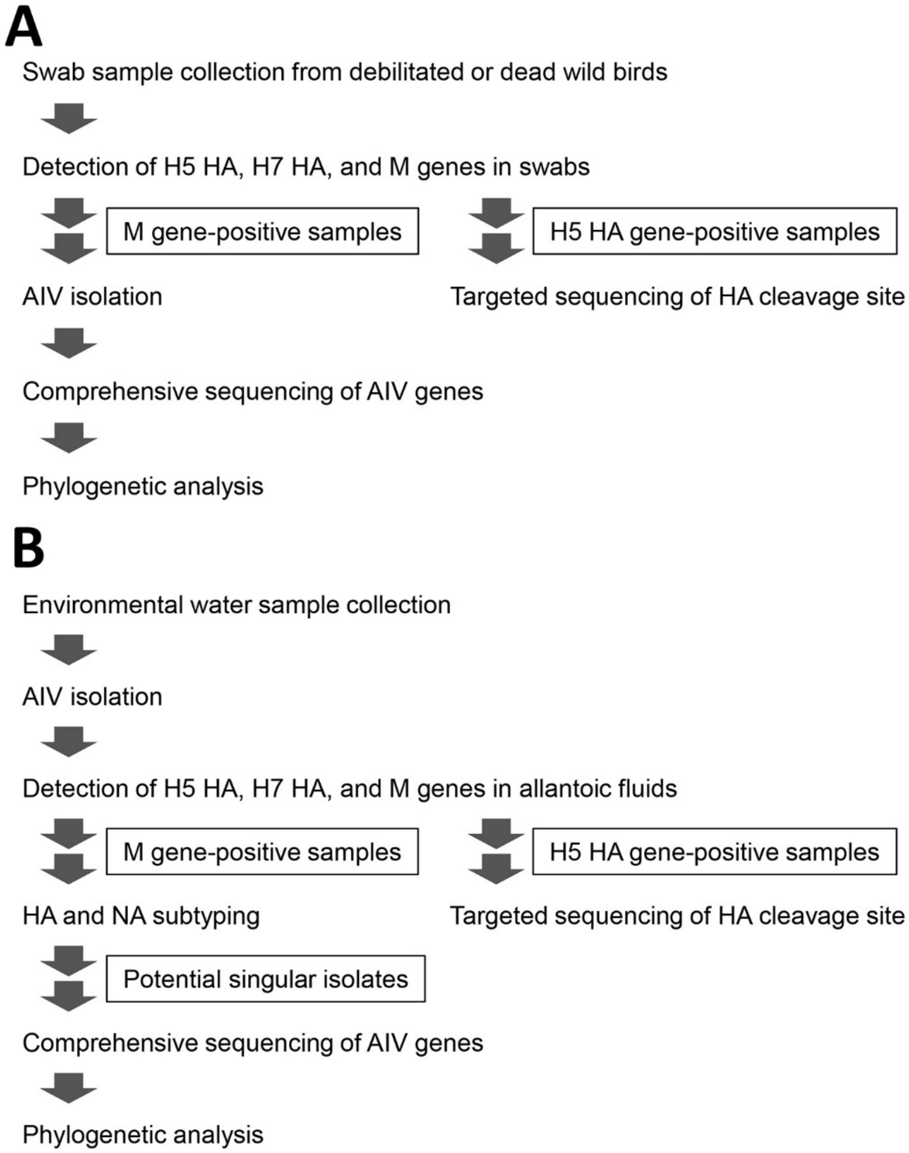

Appendix Figure 1. A workflow diagram of the AIV detection and analysis process for both water samples from crane roosts and swab samples from wild birds. A) The analysis process for swab samples from wild birds. B) The analysis process for environmental water samples from crane roosts. AIV, avian influenza virus.

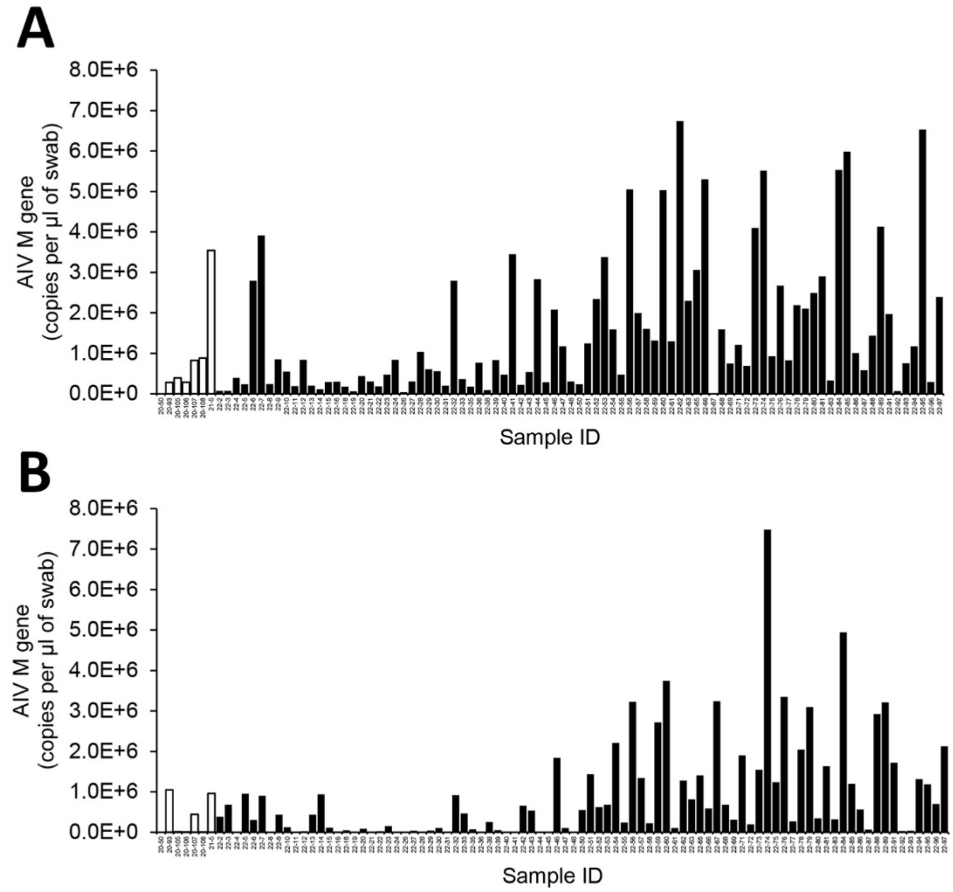

Appendix Figure 2. Copy numbers of the AIV M gene in swabs from AIV gene–positive cranes. RNA extracted from each AIV-gene–positive swab sample was used for the quantification of the copy number of the AIV M gene using real-time RT-PCR. Copy numbers of genes in the tracheal (A) and cloacal (B) swabs of 86 cranes collected during November 1–November 21, 2022, are shown. Samples collected during the 2022–23 season and previous seasons are shown in black and white, respectively. AIV, avian influenza virus; M, matrix protein.

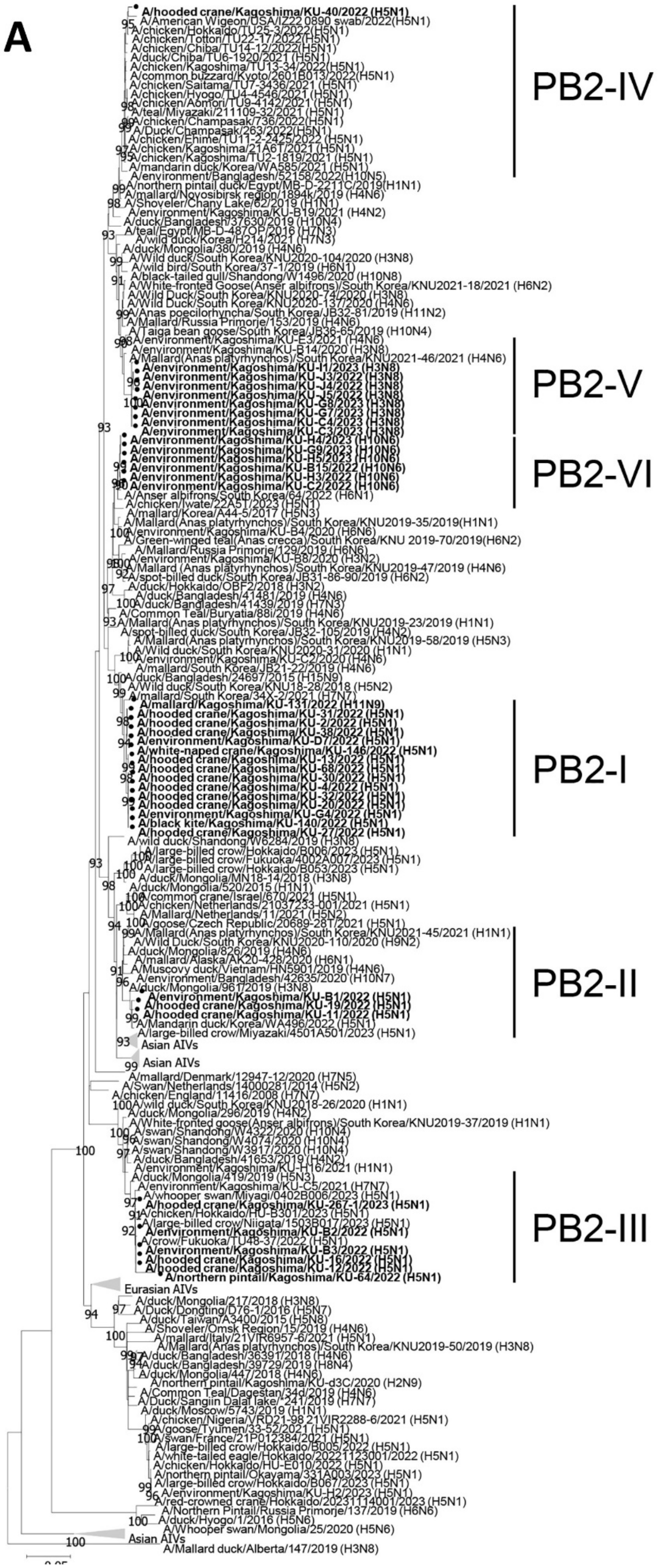
A
PB2-IV
PB2-V
PB2-VI
PB2-I
PB2-II
PB2-III
Asian AIVs
Eurasian AIVs

B

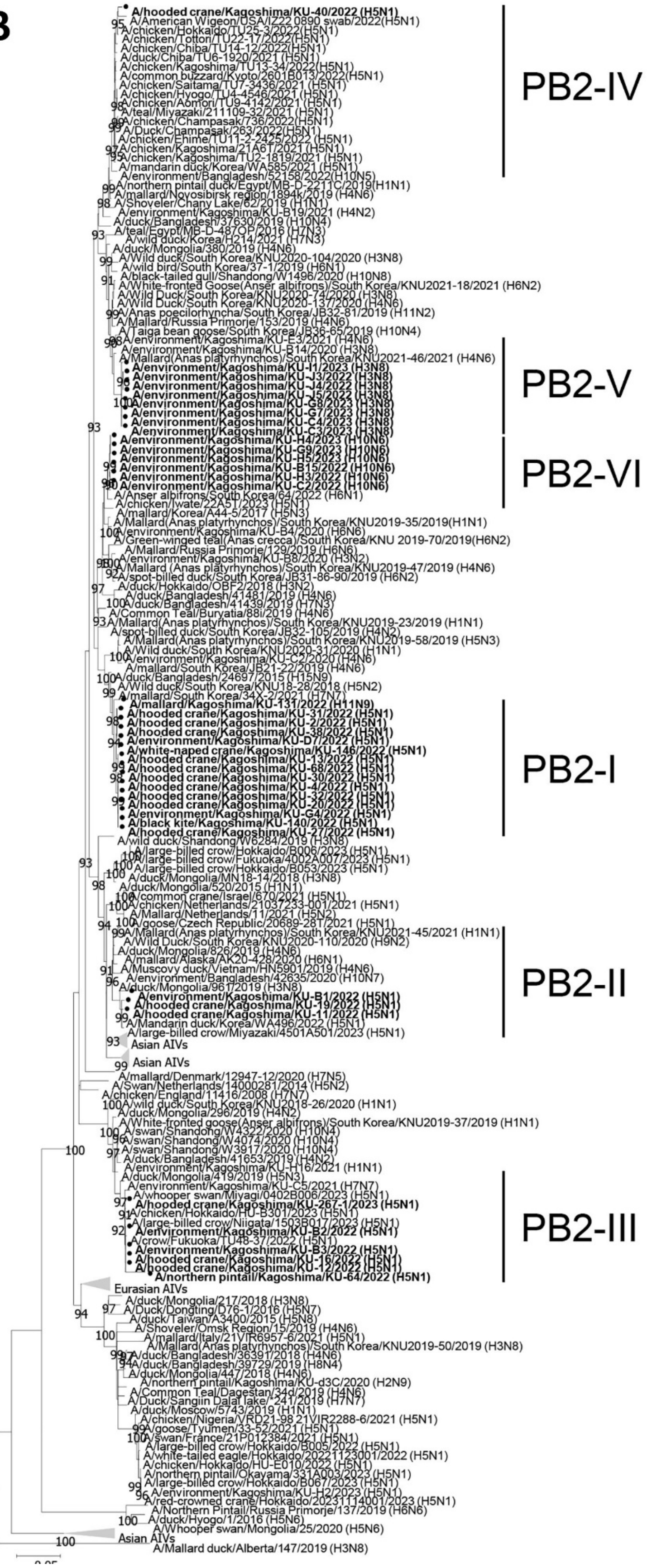
PB2-IV
PB2-V
PB2-VI
PB2-I
PB2-II
Asian AIVs
Asian AIVs
PB2-III
Eurasian AIVs
Asian AIVs

C

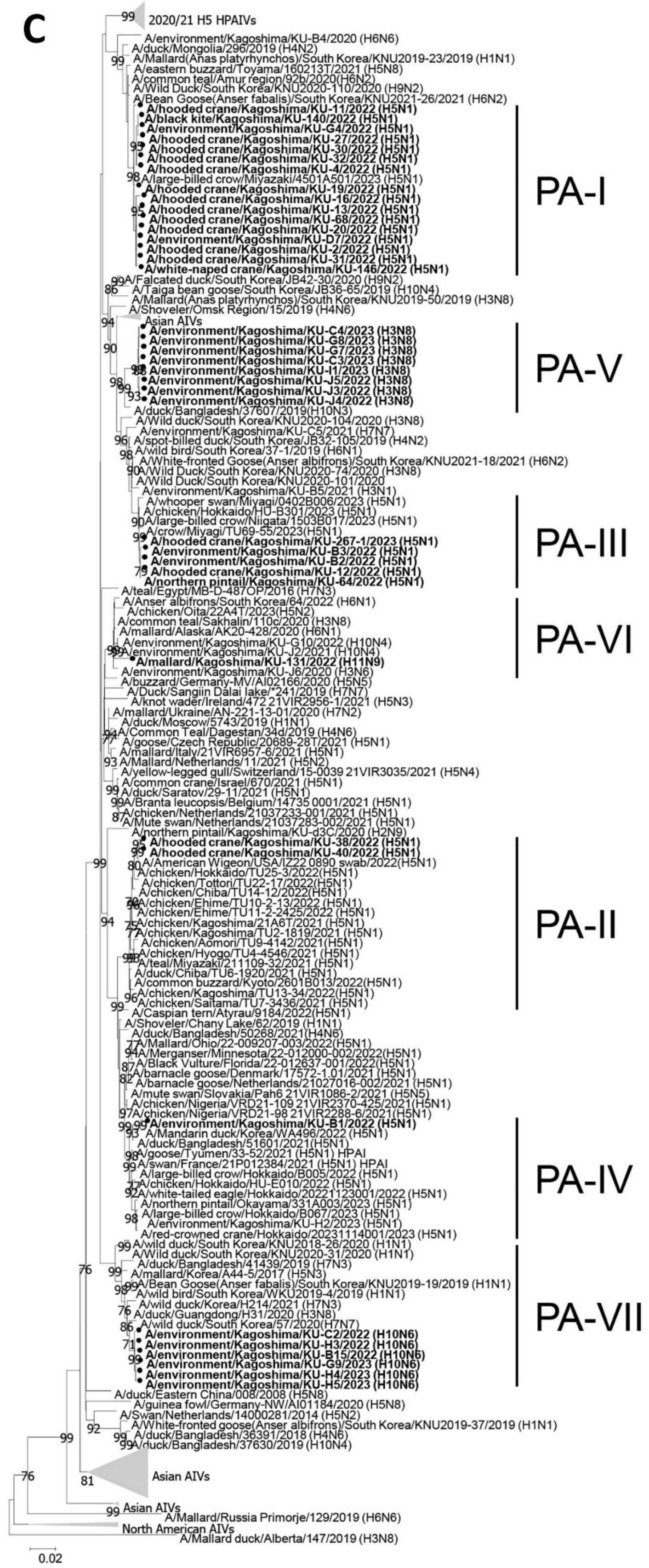
2020/21 H5 HPAIVs
A/environment/Kagoshima/KU-B4/2020 (H6N6)
A/duck/Mongolia/296/2019 (H4N2)
A/Mallard(Anas platyrhynchos)/South Korea/KNU2019-23/2019 (H1N1)
A/eastern buzzard/Toyama/160213T/2021 (H5N8)
A/common teal/Amur region/92b/2020(H6N2)
A/Wild Duck/South Korea/KNU2020-110/2020 (H9N2)
A/Bean Goose(Anser fabalis)/South Korea/KNU2021-26/2021 (H6N2)
A/hooded crane/Kagoshima/KU-11/2022 (H5N1)
A/black kite/Kagoshima/KU-140/2022 (H5N1)
A/environment/Kagoshima/KU-G4/2022 (H5N1)
A/hooded crane/Kagoshima/KU-27/2022 (H5N1)
A/hooded crane/Kagoshima/KU-30/2022 (H5N1)
A/hooded crane/Kagoshima/KU-32/2022 (H5N1)
A/hooded crane/Kagoshima/KU-4/2022 (H5N1)
A/large-billed crow/Miyazaki/4501A501/2023 (H5N1)
A/hooded crane/Kagoshima/KU-19/2022 (H5N1)
A/hooded crane/Kagoshima/KU-16/2022 (H5N1)
A/hooded crane/Kagoshima/KU-13/2022 (H5N1)
A/hooded crane/Kagoshima/KU-68/2022 (H5N1)
A/hooded crane/Kagoshima/KU-20/2022 (H5N1)
A/environment/Kagoshima/KU-D7/2022 (H5N1)
A/hooded crane/Kagoshima/KU-2/2022 (H5N1)
A/hooded crane/Kagoshima/KU-31/2022 (H5N1)
A/white-naped crane/Kagoshima/KU-146/2022 (H5N1)
PA-I
A/Falcated duck/South Korea/JB42-30/2020 (H9N2)
A/Taiga bean goose/South Korea/JB36-65/2019 (H10N4)
A/Mallard(Anas platyrhynchos)/South Korea/KNU2019-50/2019 (H3N8)
A/Shoveler/Omsk Region/15/2019 (H4N6)
Asian AIVs
A/environment/Kagoshima/KU-C4/2023 (H3N8)
A/environment/Kagoshima/KU-G8/2023 (H3N8)
A/environment/Kagoshima/KU-G7/2023 (H3N8)
A/environment/Kagoshima/KU-C3/2023 (H3N8)
A/environment/Kagoshima/KU-I1/2023 (H3N8)
A/environment/Kagoshima/KU-J5/2022 (H3N8)
A/environment/Kagoshima/KU-J3/2022 (H3N8)
A/environment/Kagoshima/KU-J4/2022 (H3N8)
PA-V
A/duck/Bangladesh/37607/2019(H10N3)
A/Wild duck/South Korea/KNU2020-104/2020 (H3N8)
A/environment/Kagoshima/KU-C5/2021 (H7N7)
A/spot-billed duck/South Korea/JB32-105/2019 (H4N2)
A/wild bird/South Korea/37-1/2019 (H6N1)
A/White-fronted Goose(Anser albifrons)/South Korea/KNU2021-18/2021 (H6N2)
A/Wild Duck/South Korea/KNU2020-74/2020 (H3N8)
A/Wild Duck/South Korea/KNU2020-101/2020
A/environment/Kagoshima/KU-B5/2021 (H3N1)
A/whooper swan/Miyagi/0402B006/2023 (H5N1)
A/chicken/Hokkaido/HU-B301/2023 (H5N1)
A/large-billed crow/Niigata/1503B017/2023 (H5N1)
A/crow/Miyagi/TU69-55/2023(H5N1)
A/hooded crane/Kagoshima/KU-267-1/2023 (H5N1)
A/environment/Kagoshima/KU-B3/2022 (H5N1)
A/environment/Kagoshima/KU-B2/2022 (H5N1)
A/hooded crane/Kagoshima/KU-12/2022 (H5N1)
A/northern pintail/Kagoshima/KU-64/2022 (H5N1)
PA-III
A/teal/Egypt/MB-D-487OP/2016 (H7N3)
A/Anser albifrons/South Korea/64/2022 (H6N1)
A/chicken/Oita/22A4T/2023(H5N2)
A/common teal/Sakhalin/110c/2020 (H3N8)
A/mallard/Alaska/AK20-428/2020 (H6N1)
A/environment/Kagoshima/KU-G10/2022 (H10N4)
A/environment/Kagoshima/KU-J2/2021 (H10N4)
A/mallard/Kagoshima/KU-131/2022 (H11N9)
A/environment/Kagoshima/KU-J6/2020 (H3N6)
PA-VI
A/buzzard/Germany-MV/AI02166/2020 (H5N5)
A/Duck/Sangiin Dalai lake/*241/2019 (H7N7)
A/knot wader/Ireland/472 21VIR2956-1/2021 (H5N3)
A/mallard/Ukraine/AN-221-13-01/2020 (H7N2)
A/duck/Moscow/5743/2019 (H1N1)
A/Common Teal/Dagestan/34d/2019 (H4N6)
A/goose/Czech Republic/20689-28T/2021 (H5N1)
A/mallard/Italy/21VIR6957-6/2021 (H5N1)
A/Mallard/Netherlands/11/2021 (H5N2)
A/yellow-legged gull/Switzerland/15-0039 21VIR3035/2021 (H5N4)
A/common crane/Israel/670/2021 (H5N1)
A/duck/Saratov/29-11/2021 (H5N1)
A/Branta leucopsis/Belgium/14735 0001/2021 (H5N1)
A/chicken/Netherlands/21037233-001/2021 (H5N1)
A/Mute swan/Netherlands/21037283-002/2021 (H5N1)
A/northern pintail/Kagoshima/KU-d3C/2020 (H2N9)
A/hooded crane/Kagoshima/KU-38/2022 (H5N1)
A/hooded crane/Kagoshima/KU-40/2022 (H5N1)
A/American Wigeon/USA/IZ22 0890 swab/2022(H5N1)
A/chicken/Hokkaido/TU25-3/2022(H5N1)
A/chicken/Tottori/TU22-17/2022(H5N1)
A/chicken/Chiba/TU14-12/2022(H5N1)
A/chicken/Ehime/TU10-2-13/2022 (H5N1)
A/chicken/Ehime/TU11-2-2425/2022 (H5N1)
A/chicken/Kagoshima/21A6T/2021 (H5N1)
A/chicken/Kagoshima/TU2-1819/2021 (H5N1)
A/chicken/Aomori/TU9-4142/2021(H5N1)
A/chicken/Hyogo/TU4-4546/2021 (H5N1)
A/teal/Miyazaki/211109-32/2021 (H5N1)
A/duck/Chiba/TU6-1920/2021 (H5N1)
A/common buzzard/Kyoto/2601B013/2022(H5N1)
A/chicken/Kagoshima/TU13-34/2022(H5N1)
A/chicken/Saitama/TU7-3436/2021 (H5N1)
PA-II
A/Caspian tern/Atyrau/9184/2022(H5N1)
A/Shoveler/Chany Lake/62/2019 (H1N1)
A/duck/Bangladesh/50268/2021(H4N6)
A/Mallard/Ohio/22-009207-003/2022(H5N1)
A/Merganser/Minnesota/22-012000-002/2022(H5N1)
A/Black Vulture/Florida/22-012637-001/2022(H5N1)
A/barnacle goose/Denmark/17572-1.01/2021 (H5N1)
A/barnacle goose/Netherlands/21027016-002/2021 (H5N1)
A/mute swan/Slovakia/Pah6 21VIR1086-2/2021 (H5N5)
A/chicken/Nigeria/VRD21-109 21VIR2370-425/2021(H5N1)
A/chicken/Nigeria/VRD21-98 21VIR2288-6/2021(H5N1)
A/environment/Kagoshima/KU-B1/2022 (H5N1)
A/Mandarin duck/Korea/WA496/2022 (H5N1)
A/duck/Bangladesh/51601/2021(H5N1)
A/goose/Tyumen/33-52/2021 (H5N1) HPAI
A/swan/France/21P012384/2021 (H5N1) HPAI
A/large-billed crow/Hokkaido/B005/2022 (H5N1)
A/chicken/Hokkaido/HU-E010/2022 (H5N1)
A/white-tailed eagle/Hokkaido/20221123001/2022 (H5N1)
A/northern pintail/Okayama/331A003/2023 (H5N1)
A/large-billed crow/Hokkaido/B067/2023 (H5N1)
A/environment/Kagoshima/KU-H2/2023 (H5N1)
A/red-crowned crane/Hokkaido/20231114001/2023 (H5N1)
PA-IV
A/wild duck/South Korea/KNU2018-26/2020 (H1N1)
A/Wild duck/South Korea/KNU2020-31/2020 (H1N1)
A/duck/Bangladesh/41439/2019 (H7N3)
A/mallard/Korea/A44-5/2017 (H5N3)
A/Bean Goose(Anser fabalis)/South Korea/KNU2019-19/2019 (H1N1)
A/wild bird/South Korea/WKU2019-4/2019 (H1N1)
A/wild duck/Korea/H214/2021 (H7N3)
A/duck/Guangdong/H31/2020 (H3N8)
A/wild duck/South Korea/57/2020(H7N7)
A/environment/Kagoshima/KU-C2/2022 (H10N6)
A/environment/Kagoshima/KU-H3/2022 (H10N6)
A/environment/Kagoshima/KU-B15/2022 (H10N6)
A/environment/Kagoshima/KU-G9/2023 (H10N6)
A/environment/Kagoshima/KU-H4/2023 (H10N6)
A/environment/Kagoshima/KU-H5/2023 (H10N6)
PA-VII
A/duck/Eastern China/008/2008 (H5N8)
A/guinea fowl/Germany-NW/AI01184/2020 (H5N8)
A/Swan/Netherlands/14000281/2014 (H5N2)
A/White-fronted goose(Anser albifrons)/South Korea/KNU2019-37/2019 (H1N1)
A/duck/Bangladesh/36391/2018 (H4N6)
A/duck/Bangladesh/37630/2019 (H10N4)
Asian AIVs
Asian AIVs
A/Mallard/Russia Primorje/129/2019 (H6N6)
North American AIVs
A/Mallard duck/Alberta/147/2019 (H3N8)
0.02

D

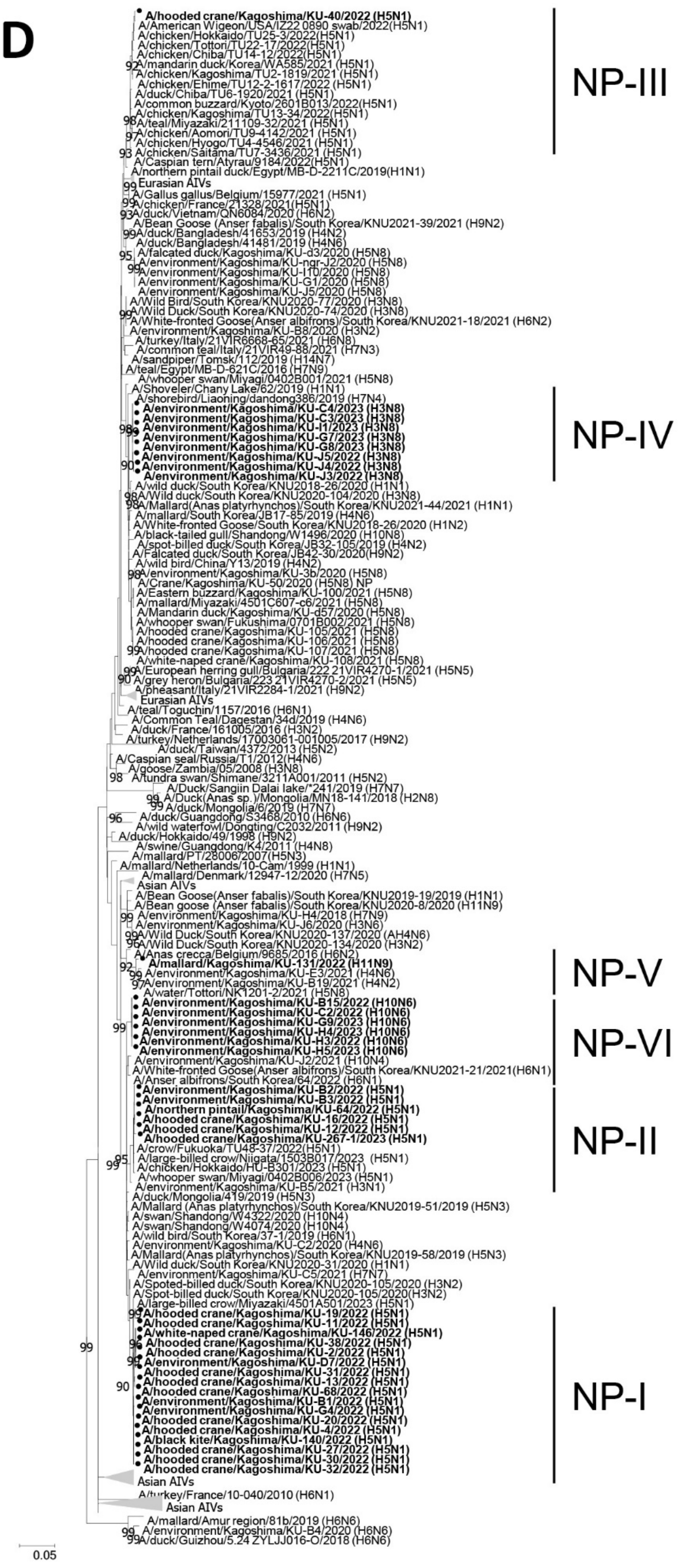
A/hooded crane/Kagoshima/KU-40/2022 (H5N1)
A/American Wigeon/USA/IZ22 0890 swab/2022(H5N1)
A/chicken/Hokkaido/TU25-3/2022(H5N1)
A/chicken/Tottori/TU22-17/2022(H5N1)
A/chicken/Chiba/TU14-12/2022(H5N1)
A/mandarin duck/Korea/WA585/2021 (H5N1)
A/chicken/Kagoshima/TU2-1819/2021 (H5N1)
A/chicken/Ehime/TU12-2-1617/2022 (H5N1)
A/duck/Chiba/TU6-1920/2021 (H5N1)
A/common buzzard/Kyoto/2601B013/2022(H5N1)
A/chicken/Kagoshima/TU13-34/2022(H5N1)
A/teal/Miyazaki/211109-32/2021 (H5N1)
A/chicken/Aomori/TU9-4142/2021 (H5N1)
A/chicken/Hyogo/TU4-4546/2021 (H5N1)
A/chicken/Saitama/TU7-3436/2021 (H5N1)
A/Caspian tern/Atyrau/9184/2022(H5N1)
A/northern pintail duck/Egypt/MB-D-2211C/2019(H1N1)
Eurasian AIVs
A/Gallus gallus/Belgium/15977/2021 (H5N1)
A/chicken/France/21328/2021(H5N1)
A/duck/Vietnam/QN6084/2020 (H6N2)
A/Bean Goose (Anser fabalis)/South Korea/KNU2021-39/2021 (H9N2)
A/duck/Bangladesh/41653/2019 (H4N2)
A/duck/Bangladesh/41481/2019 (H4N6)
A/falcated duck/Kagoshima/KU-d3/2020 (H5N8)
A/environment/Kagoshima/KU-ngr-J2/2020 (H5N8)
A/environment/Kagoshima/KU-I10/2020 (H5N8)
A/environment/Kagoshima/KU-G1/2020 (H5N8)
A/environment/Kagoshima/KU-J5/2020 (H5N8)
A/Wild Bird/South Korea/KNU2020-77/2020 (H3N8)
A/Wild Duck/South Korea/KNU2020-74/2020 (H3N8)
A/White-fronted Goose(Anser albifrons)/South Korea/KNU2021-18/2021 (H6N2)
A/environment/Kagoshima/KU-B8/2020 (H3N2)
A/turkey/Italy/21VIR6668-65/2021 (H6N8)
A/common teal/Italy/21VIR49-88/2021 (H7N3)
A/sandpiper/Tomsk/112/2019 (H14N7)
A/teal/Egypt/MB-D-621C/2016 (H7N9)
A/whooper swan/Miyagi/0402B001/2021 (H5N8)
A/Shoveler/Chany Lake/62/2019 (H1N1)
A/shorebird/Liaoning/dandong386/2019 (H7N4)
A/environment/Kagoshima/KU-C4/2023 (H3N8)
A/environment/Kagoshima/KU-C3/2023 (H3N8)
A/environment/Kagoshima/KU-I1/2023 (H3N8)
A/environment/Kagoshima/KU-G7/2023 (H3N8)
A/environment/Kagoshima/KU-G8/2023 (H3N8)
A/environment/Kagoshima/KU-J5/2022 (H3N8)
A/environment/Kagoshima/KU-J4/2022 (H3N8)
A/environment/Kagoshima/KU-J3/2022 (H3N8)
A/wild duck/South Korea/KNU2018-26/2020 (H1N1)
A/Wild duck/South Korea/KNU2020-104/2020 (H3N8)
A/Mallard(Anas platyrhynchos)/South Korea/KNU2021-44/2021 (H1N1)
A/mallard/South Korea/JB17-85/2019 (H4N6)
A/White-fronted Goose/South Korea/KNU2018-26/2020 (H1N2)
A/black-tailed gull/Shandong/W1496/2020 (H10N8)
A/spot-billed duck/South Korea/JB32-105/2019 (H4N2)
A/Falcated duck/South Korea/JB42-30/2020(H9N2)
A/wild bird/China/Y13/2019 (H4N2)
A/environment/Kagoshima/KU-3b/2020 (H5N8)
A/Crane/Kagoshima/KU-50/2020 (H5N8) NP
A/Eastern buzzard/Kagoshima/KU-100/2021 (H5N8)
A/mallard/Miyazaki/4501C607-c6/2021 (H5N8)
A/Mandarin duck/Kagoshima/KU-d57/2020 (H5N8)
A/whooper swan/Fukushima/0701B002/2021 (H5N8)
A/hooded crane/Kagoshima/KU-105/2021 (H5N8)
A/hooded crane/Kagoshima/KU-106/2021 (H5N8)
A/hooded crane/Kagoshima/KU-107/2021 (H5N8)
A/white-naped crane/Kagoshima/KU-108/2021 (H5N8)
A/European herring gull/Bulgaria/222 21VIR4270-1/2021 (H5N5)
A/grey heron/Bulgaria/223 21VIR4270-2/2021 (H5N5)
A/pheasant/Italy/21VIR2284-1/2021 (H9N2)
Eurasian AIVs
A/teal/Toguchin/1157/2016 (H6N1)
A/Common Teal/Dagestan/34d/2019 (H4N6)
A/duck/France/161005/2016 (H3N2)
A/turkey/Netherlands/17003061-001005/2017 (H9N2)
A/duck/Taiwan/4372/2013 (H5N2)
A/Caspian seal/Russia/T1/2012(H4N6)
A/goose/Zambia/05/2008 (H3N8)
A/tundra swan/Shimane/3211A001/2011 (H5N2)
A/Duck/Sangiin Dalai lake/*241/2019 (H7N7)
A/Duck(Anas sp.)/Mongolia/MN18-141/2018 (H2N8)
A/duck/Mongolia/6/2019 (H7N7)
A/duck/Guangdong/S3468/2010 (H6N6)
A/wild waterfowl/Dongting/C2032/2011 (H9N2)
A/duck/Hokkaido/49/1998 (H9N2)
A/swine/Guangdong/K4/2011 (H4N8)
A/mallard/PT/28006/2007(H5N3)
A/mallard/Netherlands/10-Cam/1999 (H1N1)
A/mallard/Denmark/12947-12/2020 (H7N5)
Asian AIVs
A/Bean Goose(Anser fabalis)/South Korea/KNU2019-19/2019 (H1N1)
A/Bean goose (Anser fabalis)/South Korea/KNU2020-8/2020 (H11N9)
A/environment/Kagoshima/KU-H4/2018 (H7N9)
A/environment/Kagoshima/KU-J6/2020 (H3N6)
A/Wild Duck/South Korea/KNU2020-137/2020 (AH4N6)
A/Wild Duck/South Korea/KNU2020-134/2020 (H3N2)
A/Anas crecca/Belgium/9685/2016 (H6N2)
A/mallard/Kagoshima/KU-131/2022 (H11N9)
A/environment/Kagoshima/KU-E3/2021 (H4N6)
A/environment/Kagoshima/KU-B19/2021 (H4N2)
A/water/Tottori/NK1201-2/2021 (H5N8)
A/environment/Kagoshima/KU-B15/2022 (H10N6)
A/environment/Kagoshima/KU-C2/2022 (H10N6)
A/environment/Kagoshima/KU-G9/2023 (H10N6)
A/environment/Kagoshima/KU-H4/2023 (H10N6)
A/environment/Kagoshima/KU-H3/2022 (H10N6)
A/environment/Kagoshima/KU-H5/2023 (H10N6)
A/environment/Kagoshima/KU-J2/2021 (H10N4)
A/White-fronted Goose(Anser albifrons)/South Korea/KNU2021-21/2021(H6N1)
A/Anser albifrons/South Korea/64/2022 (H6N1)
A/environment/Kagoshima/KU-B2/2022 (H5N1)
A/environment/Kagoshima/KU-B3/2022 (H5N1)
A/northern pintail/Kagoshima/KU-64/2022 (H5N1)
A/hooded crane/Kagoshima/KU-16/2022 (H5N1)
A/hooded crane/Kagoshima/KU-12/2022 (H5N1)
A/hooded crane/Kagoshima/KU-267-1/2023 (H5N1)
A/crow/Fukuoka/TU48-37/2022(H5N1)
A/large-billed crow/Niigata/1503B017/2023 (H5N1)
A/chicken/Hokkaido/HU-B301/2023 (H5N1)
A/whooper swan/Miyagi/0402B006/2023 (H5N1)
A/environment/Kagoshima/KU-B5/2021 (H3N1)
A/duck/Mongolia/419/2019 (H5N3)
A/Mallard (Anas platyrhynchos)/South Korea/KNU2019-51/2019 (H5N3)
A/swan/Shandong/W4322/2020 (H10N4)
A/swan/Shandong/W4074/2020 (H10N4)
A/wild bird/South Korea/37-1/2019 (H6N1)
A/environment/Kagoshima/KU-C2/2020 (H4N6)
A/Mallard(Anas platyrhynchos)/South Korea/KNU2019-58/2019 (H5N3)
A/Wild duck/South Korea/KNU2020-31/2020 (H1N1)
A/environment/Kagoshima/KU-C5/2021 (H7N7)
A/Spoted-billed duck/South Korea/KNU2020-105/2020 (H3N2)
A/Spot-billed duck/South Korea/KNU2020-105/2020(H3N2)
A/large-billed crow/Miyazaki/4501A501/2023 (H5N1)
A/hooded crane/Kagoshima/KU-19/2022 (H5N1)
A/hooded crane/Kagoshima/KU-11/2022 (H5N1)
A/white-naped crane/Kagoshima/KU-146/2022 (H5N1)
A/hooded crane/Kagoshima/KU-38/2022 (H5N1)
A/hooded crane/Kagoshima/KU-2/2022 (H5N1)
A/environment/Kagoshima/KU-D7/2022 (H5N1)
A/hooded crane/Kagoshima/KU-31/2022 (H5N1)
A/hooded crane/Kagoshima/KU-13/2022 (H5N1)
A/hooded crane/Kagoshima/KU-68/2022 (H5N1)
A/environment/Kagoshima/KU-B1/2022 (H5N1)
A/environment/Kagoshima/KU-G4/2022 (H5N1)
A/hooded crane/Kagoshima/KU-20/2022 (H5N1)
A/hooded crane/Kagoshima/KU-4/2022 (H5N1)
A/black kite/Kagoshima/KU-140/2022 (H5N1)
A/hooded crane/Kagoshima/KU-27/2022 (H5N1)
A/hooded crane/Kagoshima/KU-30/2022 (H5N1)
A/hooded crane/Kagoshima/KU-32/2022 (H5N1)
Asian AIVs
A/turkey/France/10-040/2010 (H6N1)
Asian AIVs
A/mallard/Amur region/81b/2019 (H6N6)
A/environment/Kagoshima/KU-B4/2020 (H6N6)
A/duck/Guizhou/5.24 ZYLJJ016-O/2018 (H6N6)
NP-III
NP-IV
NP-V
NP-VI
NP-II
NP-I
0.05

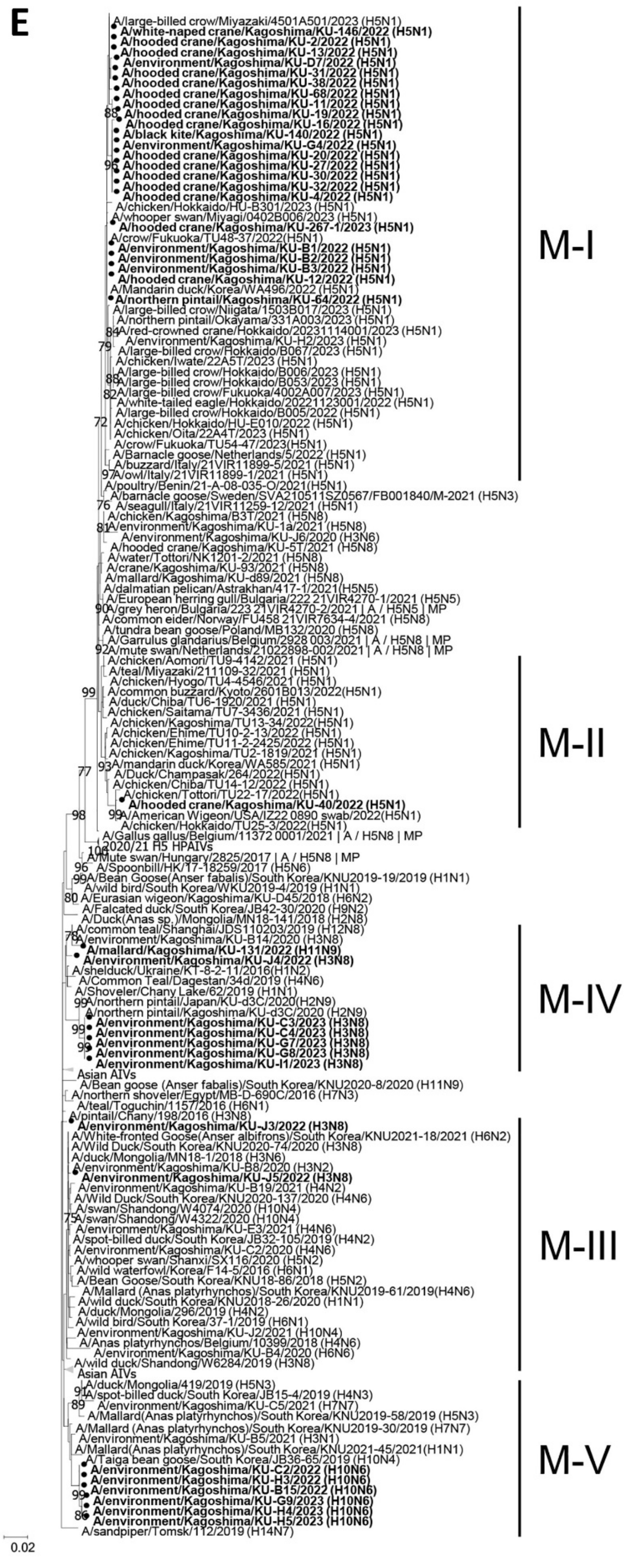
E
M-I
M-II
M-IV
M-III
M-V
0.02

F

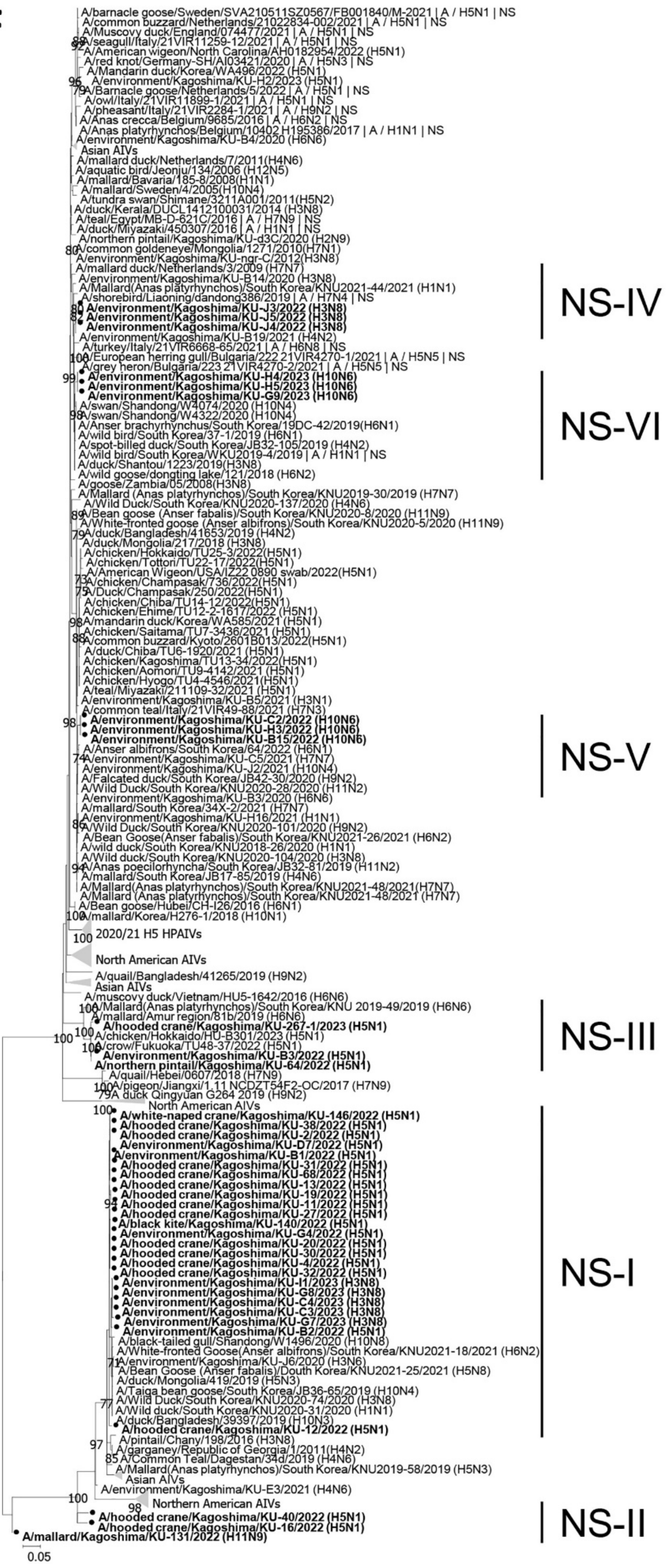

Appendix Figure 3. Phylogenetic trees of PB2, PB1, PA, NP, M, and NS genes. Phylogenetic trees of PB2 (A), PB1 (B), PA (C), NP (D), M (E), and NS (F) gene segments were constructed. AIVs isolated in this study are marked with black circles. The maximum likelihood method with a bootstrapping set of 1,000 replicates was employed, and nodes with bootstrap values exceeding 90% are displayed. The scale bar represents the number of nucleotide substitutions per site. M, matrix protein; NP, nucleoprotein; NS, nonstructural protein; PA, polymerase; PB, polymerase basic.

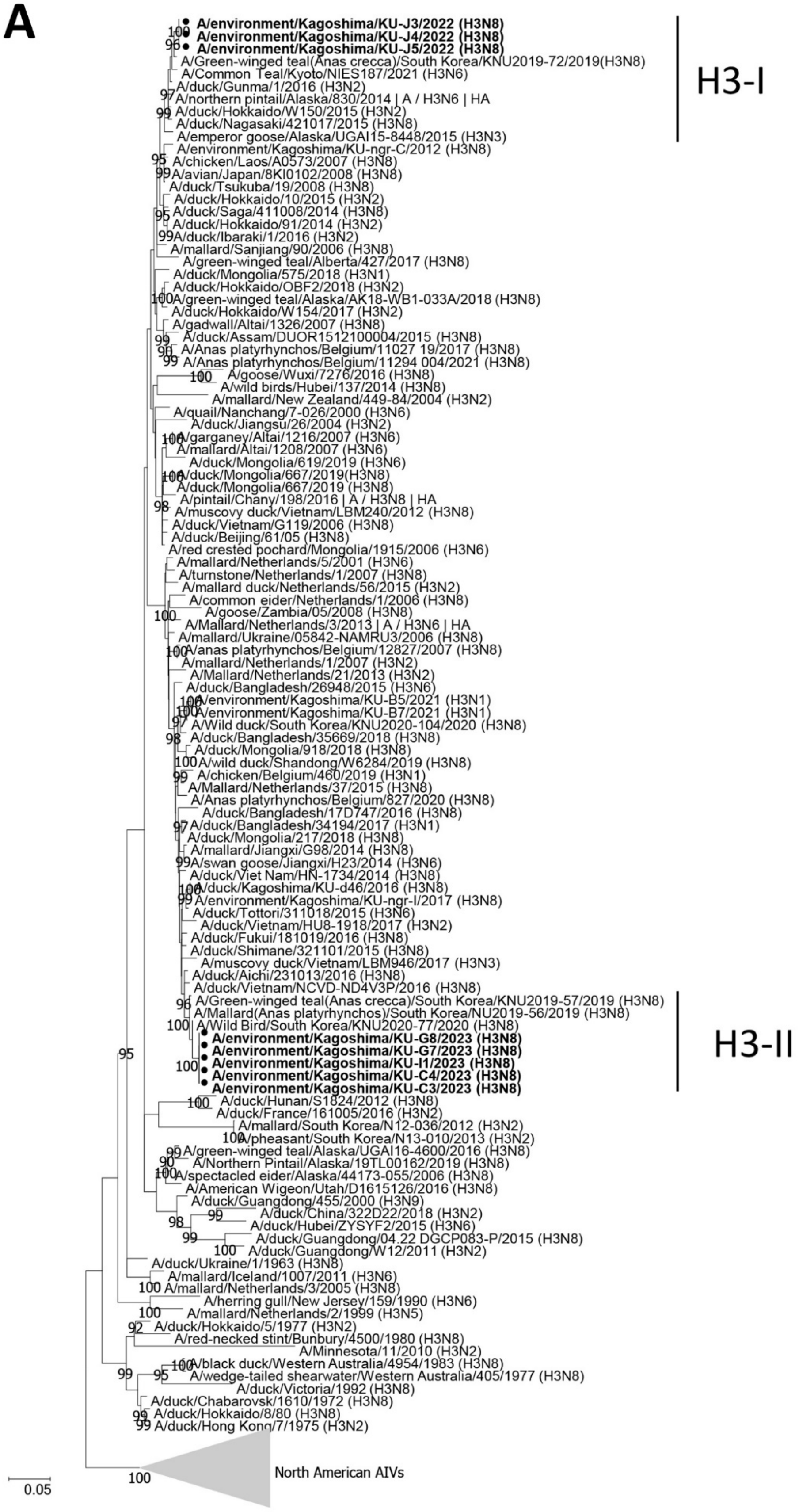
A
A/environment/Kagoshima/KU-J3/2022 (H3N8)
A/environment/Kagoshima/KU-J4/2022 (H3N8)
A/environment/Kagoshima/KU-J5/2022 (H3N8)
A/Green-winged teal(Anas crecca)/South Korea/KNU2019-72/2019(H3N8)
A/Common Teal/Kyoto/NIES187/2021 (H3N6)
A/duck/Gunma/1/2016 (H3N2)
A/northern pintail/Alaska/830/2014 | A / H3N6 | HA
A/duck/Hokkaido/W150/2015 (H3N2)
A/duck/Nagasaki/421017/2015 (H3N8)
A/emperor goose/Alaska/UGAI15-8448/2015 (H3N3)
A/environment/Kagoshima/KU-ngr-C/2012 (H3N8)
A/chicken/Laos/A0573/2007 (H3N8)
A/avian/Japan/8KI0102/2008 (H3N8)
A/duck/Tsukuba/19/2008 (H3N8)
A/duck/Hokkaido/10/2015 (H3N2)
A/duck/Saga/411008/2014 (H3N8)
A/duck/Hokkaido/91/2014 (H3N2)
A/duck/Ibaraki/1/2016 (H3N2)
A/mallard/Sanjiang/90/2006 (H3N8)
A/green-winged teal/Alberta/427/2017 (H3N8)
A/duck/Mongolia/575/2018 (H3N1)
A/duck/Hokkaido/OBF2/2018 (H3N2)
A/green-winged teal/Alaska/AK18-WB1-033A/2018 (H3N8)
A/duck/Hokkaido/W154/2017 (H3N2)
A/gadwall/Altai/1326/2007 (H3N8)
A/duck/Assam/DUOR1512100004/2015 (H3N8)
A/Anas platyrhynchos/Belgium/11027 19/2017 (H3N8)
A/Anas platyrhynchos/Belgium/11294 004/2021 (H3N8)
A/goose/Wuxi/7276/2016 (H3N8)
A/wild birds/Hubei/137/2014 (H3N8)
A/mallard/New Zealand/449-84/2004 (H3N2)
A/quail/Nanchang/7-026/2000 (H3N6)
A/duck/Jiangsu/26/2004 (H3N2)
A/garganey/Altai/1216/2007 (H3N6)
A/mallard/Altai/1208/2007 (H3N6)
A/duck/Mongolia/619/2019 (H3N6)
A/duck/Mongolia/667/2019(H3N8)
A/duck/Mongolia/667/2019 (H3N8)
A/pintail/Chany/198/2016 | A / H3N8 | HA
A/muscovy duck/Vietnam/LBM240/2012 (H3N8)
A/duck/Vietnam/G119/2006 (H3N8)
A/duck/Beijing/61/05 (H3N8)
A/red crested pochard/Mongolia/1915/2006 (H3N6)
A/mallard/Netherlands/5/2001 (H3N6)
A/turnstone/Netherlands/1/2007 (H3N8)
A/mallard duck/Netherlands/56/2015 (H3N2)
A/common eider/Netherlands/1/2006 (H3N8)
A/goose/Zambia/05/2008 (H3N8)
A/Mallard/Netherlands/3/2013 | A / H3N6 | HA
A/mallard/Ukraine/05842-NAMRU3/2006 (H3N8)
A/anas platyrhynchos/Belgium/12827/2007 (H3N8)
A/mallard/Netherlands/1/2007 (H3N2)
A/Mallard/Netherlands/21/2013 (H3N2)
A/duck/Bangladesh/26948/2015 (H3N6)
A/environment/Kagoshima/KU-B5/2021 (H3N1)
A/environment/Kagoshima/KU-B7/2021 (H3N1)
A/Wild duck/South Korea/KNU2020-104/2020 (H3N8)
A/duck/Bangladesh/35669/2018 (H3N8)
A/duck/Mongolia/918/2018 (H3N8)
A/wild duck/Shandong/W6284/2019 (H3N8)
A/chicken/Belgium/460/2019 (H3N1)
A/Mallard/Netherlands/37/2015 (H3N8)
A/Anas platyrhynchos/Belgium/827/2020 (H3N8)
A/duck/Bangladesh/17D747/2016 (H3N8)
A/duck/Bangladesh/34194/2017 (H3N1)
A/duck/Mongolia/217/2018 (H3N8)
A/mallard/Jiangxi/G98/2014 (H3N8)
A/swan goose/Jiangxi/H23/2014 (H3N6)
A/duck/Viet Nam/HN-1734/2014 (H3N8)
A/duck/Kagoshima/KU-d46/2016 (H3N8)
A/environment/Kagoshima/KU-ngr-I/2017 (H3N8)
A/duck/Tottori/311018/2015 (H3N6)
A/duck/Vietnam/HU8-1918/2017 (H3N2)
A/duck/Fukui/181019/2016 (H3N8)
A/duck/Shimane/321101/2015 (H3N8)
A/muscovy duck/Vietnam/LBM946/2017 (H3N3)
A/duck/Aichi/231013/2016 (H3N8)
A/duck/Vietnam/NCVD-ND4V3P/2016 (H3N8)
A/Green-winged teal(Anas crecca)/South Korea/KNU2019-57/2019 (H3N8)
A/Mallard(Anas platyrhynchos)/South Korea/NU2019-56/2019 (H3N8)
A/Wild Bird/South Korea/KNU2020-77/2020 (H3N8)
A/environment/Kagoshima/KU-G8/2023 (H3N8)
A/environment/Kagoshima/KU-G7/2023 (H3N8)
A/environment/Kagoshima/KU-I1/2023 (H3N8)
A/environment/Kagoshima/KU-C4/2023 (H3N8)
A/environment/Kagoshima/KU-C3/2023 (H3N8)
A/duck/Hunan/S1824/2012 (H3N8)
A/duck/France/161005/2016 (H3N2)
A/mallard/South Korea/N12-036/2012 (H3N2)
A/pheasant/South Korea/N13-010/2013 (H3N2)
A/green-winged teal/Alaska/UGAI16-4600/2016 (H3N8)
A/Northern Pintail/Alaska/19TL00162/2019 (H3N8)
A/spectacled eider/Alaska/44173-055/2006 (H3N8)
A/American Wigeon/Utah/D1615126/2016 (H3N8)
A/duck/Guangdong/455/2000 (H3N9)
A/duck/China/322D22/2018 (H3N2)
A/duck/Hubei/ZYSYF2/2015 (H3N6)
A/duck/Guangdong/04.22 DGCP083-P/2015 (H3N8)
A/duck/Guangdong/W12/2011 (H3N2)
A/duck/Ukraine/1/1963 (H3N8)
A/mallard/Iceland/1007/2011 (H3N6)
A/mallard/Netherlands/3/2005 (H3N8)
A/herring gull/New Jersey/159/1990 (H3N6)
A/mallard/Netherlands/2/1999 (H3N5)
A/duck/Hokkaido/5/1977 (H3N2)
A/red-necked stint/Bunbury/4500/1980 (H3N8)
A/Minnesota/11/2010 (H3N2)
A/black duck/Western Australia/4954/1983 (H3N8)
A/wedge-tailed shearwater/Western Australia/405/1977 (H3N8)
A/duck/Victoria/1992 (H3N8)
A/duck/Chabarovsk/1610/1972 (H3N8)
A/duck/Hokkaido/8/80 (H3N8)
A/duck/Hong Kong/7/1975 (H3N2)
North American AIVs
H3-I
H3-II
0.05

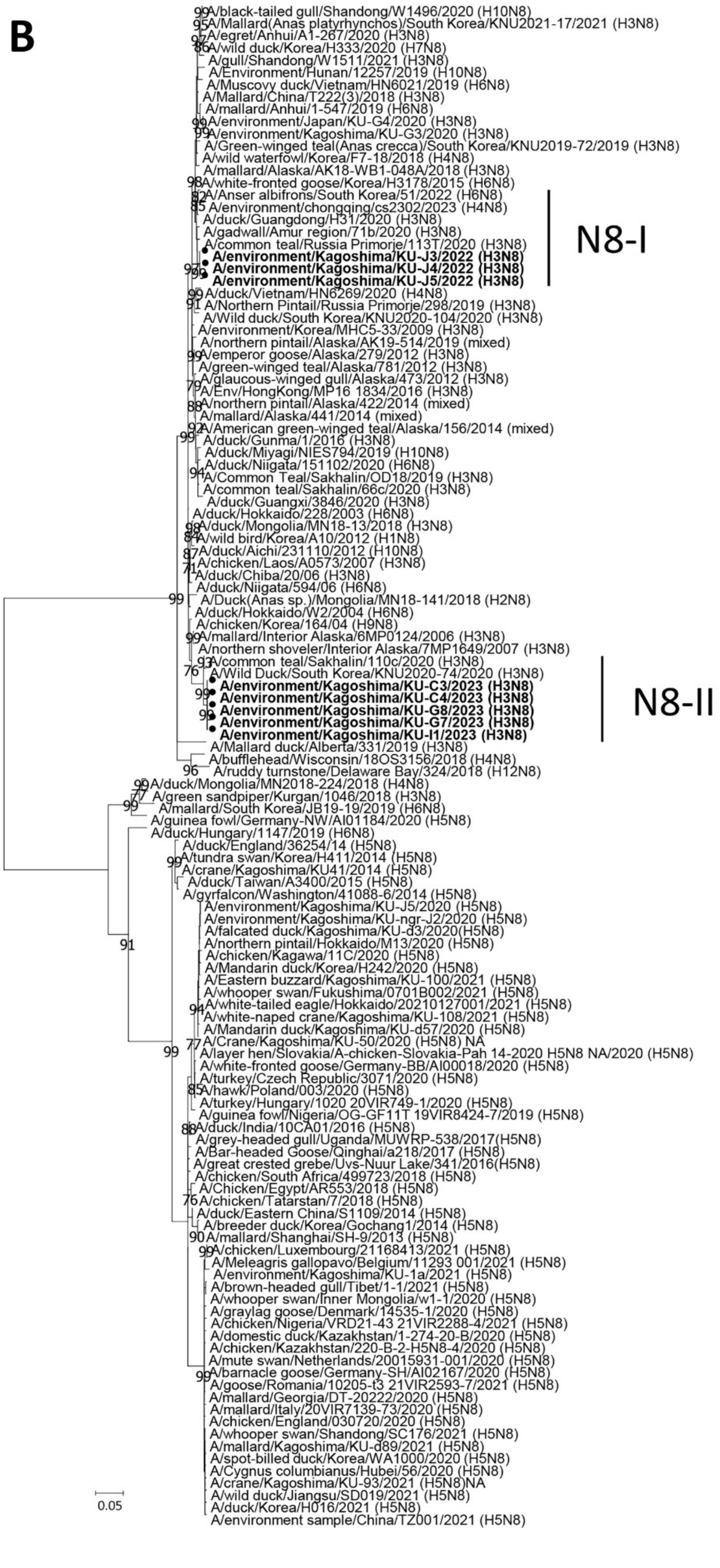
B
A/black-tailed gull/Shandong/W1496/2020 (H10N8)
A/Mallard(Anas platyrhynchos)/South Korea/KNU2021-17/2021 (H3N8)
A/egret/Anhui/A1-267/2020 (H3N8)
A/wild duck/Korea/H333/2020 (H7N8)
A/gull/Shandong/W1511/2021 (H3N8)
A/Environment/Hunan/12257/2019 (H10N8)
A/Muscovy duck/Vietnam/HN6021/2019 (H6N8)
A/Mallard/China/T222(3)/2018 (H3N8)
A/mallard/Anhui/1-547/2019 (H6N8)
A/environment/Japan/KU-G4/2020 (H3N8)
A/environment/Kagoshima/KU-G3/2020 (H3N8)
A/Green-winged teal(Anas crecca)/South Korea/KNU2019-72/2019 (H3N8)
A/wild waterfowl/Korea/F7-18/2018 (H4N8)
A/mallard/Alaska/AK18-WB1-048A/2018 (H3N8)
A/white-fronted goose/Korea/H3178/2015 (H6N8)
A/Anser albifrons/South Korea/51/2022 (H6N8)
A/environment/chongqing/cs2302/2023 (H4N8)
A/duck/Guangdong/H31/2020 (H3N8)
A/gadwall/Amur region/71b/2020 (H3N8)
A/common teal/Russia Primorje/113T/2020 (H3N8)
A/environment/Kagoshima/KU-J3/2022 (H3N8)
A/environment/Kagoshima/KU-J4/2022 (H3N8)
A/environment/Kagoshima/KU-J5/2022 (H3N8)
N8-I
A/duck/Vietnam/HN6269/2020 (H4N8)
A/Northern Pintail/Russia Primorje/298/2019 (H3N8)
A/Wild duck/South Korea/KNU2020-104/2020 (H3N8)
A/environment/Korea/MHC5-33/2009 (H3N8)
A/northern pintail/Alaska/AK19-514/2019 (mixed)
A/emperor goose/Alaska/279/2012 (H3N8)
A/green-winged teal/Alaska/781/2012 (H3N8)
A/glaucous-winged gull/Alaska/473/2012 (H3N8)
A/Env/HongKong/MP16 1834/2016 (H3N8)
A/northern pintail/Alaska/422/2014 (mixed)
A/mallard/Alaska/441/2014 (mixed)
A/American green-winged teal/Alaska/156/2014 (mixed)
A/duck/Gunma/1/2016 (H3N8)
A/duck/Miyagi/NIES794/2019 (H10N8)
A/duck/Niigata/151102/2020 (H6N8)
A/Common Teal/Sakhalin/OD18/2019 (H3N8)
A/common teal/Sakhalin/66c/2020 (H3N8)
A/duck/Guangxi/3846/2020 (H3N8)
A/duck/Hokkaido/228/2003 (H6N8)
A/duck/Mongolia/MN18-13/2018 (H3N8)
A/wild bird/Korea/A10/2012 (H1N8)
A/duck/Aichi/231110/2012 (H10N8)
A/chicken/Laos/A0573/2007 (H3N8)
A/duck/Chiba/20/06 (H3N8)
A/duck/Niigata/594/06 (H6N8)
A/Duck(Anas sp.)/Mongolia/MN18-141/2018 (H2N8)
A/duck/Hokkaido/W2/2004 (H6N8)
A/chicken/Korea/164/04 (H9N8)
A/mallard/Interior Alaska/6MP0124/2006 (H3N8)
A/northern shoveler/Interior Alaska/7MP1649/2007 (H3N8)
A/common teal/Sakhalin/110c/2020 (H3N8)
A/Wild Duck/South Korea/KNU2020-74/2020 (H3N8)
A/environment/Kagoshima/KU-C3/2023 (H3N8)
A/environment/Kagoshima/KU-C4/2023 (H3N8)
A/environment/Kagoshima/KU-G8/2023 (H3N8)
A/environment/Kagoshima/KU-G7/2023 (H3N8)
A/environment/Kagoshima/KU-I1/2023 (H3N8)
N8-II
A/Mallard duck/Alberta/331/2019 (H3N8)
A/bufflehead/Wisconsin/18OS3156/2018 (H4N8)
A/ruddy turnstone/Delaware Bay/324/2018 (H12N8)
A/duck/Mongolia/MN2018-224/2018 (H4N8)
A/green sandpiper/Kurgan/1046/2018 (H3N8)
A/mallard/South Korea/JB19-19/2019 (H6N8)
A/guinea fowl/Germany-NW/AI01184/2020 (H5N8)
A/duck/Hungary/1147/2019 (H6N8)
A/duck/England/36254/14 (H5N8)
A/tundra swan/Korea/H411/2014 (H5N8)
A/crane/Kagoshima/KU41/2014 (H5N8)
A/duck/Taiwan/A3400/2015 (H5N8)
A/gyrfalcon/Washington/41088-6/2014 (H5N8)
A/environment/Kagoshima/KU-J5/2020 (H5N8)
A/environment/Kagoshima/KU-ngr-J2/2020 (H5N8)
A/falcated duck/Kagoshima/KU-d3/2020(H5N8)
A/northern pintail/Hokkaido/M13/2020 (H5N8)
A/chicken/Kagawa/11C/2020 (H5N8)
A/Mandarin duck/Korea/H242/2020 (H5N8)
A/Eastern buzzard/Kagoshima/KU-100/2021 (H5N8)
A/whooper swan/Fukushima/0701B002/2021 (H5N8)
A/white-tailed eagle/Hokkaido/20210127001/2021 (H5N8)
A/white-naped crane/Kagoshima/KU-108/2021 (H5N8)
A/Mandarin duck/Kagoshima/KU-d57/2020 (H5N8)
A/Crane/Kagoshima/KU-50/2020 (H5N8) NA
A/layer hen/Slovakia/A-chicken-Slovakia-Pah 14-2020 H5N8 NA/2020 (H5N8)
A/white-fronted goose/Germany-BB/AI00018/2020 (H5N8)
A/turkey/Czech Republic/3071/2020 (H5N8)
A/hawk/Poland/003/2020 (H5N8)
A/turkey/Hungary/1020 20VIR749-1/2020 (H5N8)
A/guinea fowl/Nigeria/OG-GF11T 19VIR8424-7/2019 (H5N8)
A/duck/India/10CA01/2016 (H5N8)
A/grey-headed gull/Uganda/MUWRP-538/2017(H5N8)
A/Bar-headed Goose/Qinghai/a218/2017 (H5N8)
A/great crested grebe/Uvs-Nuur Lake/341/2016(H5N8)
A/chicken/South Africa/499723/2018 (H5N8)
A/Chicken/Egypt/AR553/2018 (H5N8)
A/chicken/Tatarstan/7/2018 (H5N8)
A/duck/Eastern China/S1109/2014 (H5N8)
A/breeder duck/Korea/Gochang1/2014 (H5N8)
A/mallard/Shanghai/SH-9/2013 (H5N8)
A/chicken/Luxembourg/21168413/2021 (H5N8)
A/Meleagris gallopavo/Belgium/11293 001/2021 (H5N8)
A/environment/Kagoshima/KU-1a/2021 (H5N8)
A/brown-headed gull/Tibet/1-1/2021 (H5N8)
A/whooper swan/Inner Mongolia/w1-1/2020 (H5N8)
A/graylag goose/Denmark/14535-1/2020 (H5N8)
A/chicken/Nigeria/VRD21-43 21VIR2288-4/2021 (H5N8)
A/domestic duck/Kazakhstan/1-274-20-B/2020 (H5N8)
A/chicken/Kazakhstan/220-B-2-H5N8-4/2020 (H5N8)
A/mute swan/Netherlands/20015931-001/2020 (H5N8)
A/barnacle goose/Germany-SH/AI02167/2020 (H5N8)
A/goose/Romania/10205-t3 21VIR2593-7/2021 (H5N8)
A/mallard/Georgia/DT-20222/2020 (H5N8)
A/mallard/Italy/20VIR7139-73/2020 (H5N8)
A/chicken/England/030720/2020 (H5N8)
A/whooper swan/Shandong/SC176/2021 (H5N8)
A/mallard/Kagoshima/KU-d89/2021 (H5N8)
A/spot-billed duck/Korea/WA1000/2020 (H5N8)
A/Cygnus columbianus/Hubei/56/2020 (H5N8)
A/crane/Kagoshima/KU-93/2021 (H5N8)NA
A/wild duck/Jiangsu/SD019/2021 (H5N8)
A/duck/Korea/H016/2021 (H5N8)
A/environment sample/China/TZ001/2021 (H5N8)
0.05

C

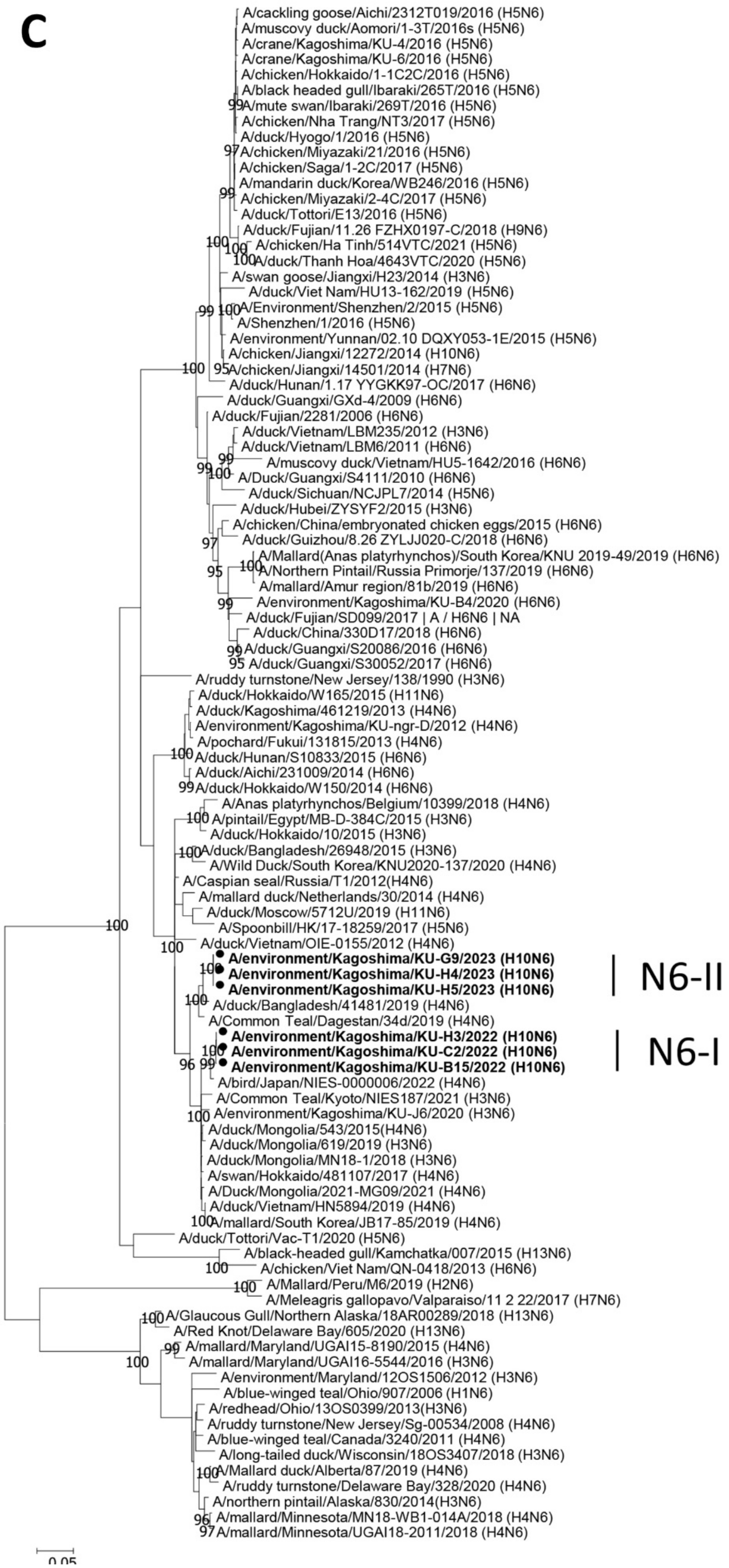

A/cackling goose/Aichi/2312T019/2016 (H5N6)
A/muscovy duck/Aomori/1-3T/2016s (H5N6)
A/crane/Kagoshima/KU-4/2016 (H5N6)
A/crane/Kagoshima/KU-6/2016 (H5N6)
A/chicken/Hokkaido/1-1C2C/2016 (H5N6)
A/black headed gull/Ibaraki/265T/2016 (H5N6)
A/mute swan/Ibaraki/269T/2016 (H5N6)
A/chicken/Nha Trang/NT3/2017 (H5N6)
A/duck/Hyogo/1/2016 (H5N6)
A/chicken/Miyazaki/21/2016 (H5N6)
A/chicken/Saga/1-2C/2017 (H5N6)
A/mandarin duck/Korea/WB246/2016 (H5N6)
A/chicken/Miyazaki/2-4C/2017 (H5N6)
A/duck/Tottori/E13/2016 (H5N6)
A/duck/Fujian/11.26 FZHX0197-C/2018 (H9N6)
A/chicken/Ha Tinh/514VTC/2021 (H5N6)
A/duck/Thanh Hoa/4643VTC/2020 (H5N6)
A/swan goose/Jiangxi/H23/2014 (H3N6)
A/duck/Viet Nam/HU13-162/2019 (H5N6)
A/Environment/Shenzhen/2/2015 (H5N6)
A/Shenzhen/1/2016 (H5N6)
A/environment/Yunnan/02.10 DQXY053-1E/2015 (H5N6)
A/chicken/Jiangxi/12272/2014 (H10N6)
A/chicken/Jiangxi/14501/2014 (H7N6)
A/duck/Hunan/1.17 YYGKK97-OC/2017 (H6N6)
A/duck/Guangxi/GXd-4/2009 (H6N6)
A/duck/Fujian/2281/2006 (H6N6)
A/duck/Vietnam/LBM235/2012 (H3N6)
A/duck/Vietnam/LBM6/2011 (H6N6)
A/muscovy duck/Vietnam/HU5-1642/2016 (H6N6)
A/Duck/Guangxi/S4111/2010 (H6N6)
A/duck/Sichuan/NCJPL7/2014 (H5N6)
A/duck/Hubei/ZYSYF2/2015 (H3N6)
A/chicken/China/embryonated chicken eggs/2015 (H6N6)
A/duck/Guizhou/8.26 ZYLJJ020-C/2018 (H6N6)
A/Mallard(Anas platyrhynchos)/South Korea/KNU 2019-49/2019 (H6N6)
A/Northern Pintail/Russia Primorje/137/2019 (H6N6)
A/mallard/Amur region/81b/2019 (H6N6)
A/environment/Kagoshima/KU-B4/2020 (H6N6)
A/duck/Fujian/SD099/2017 | A / H6N6 | NA
A/duck/China/330D17/2018 (H6N6)
A/duck/Guangxi/S20086/2016 (H6N6)
A/duck/Guangxi/S30052/2017 (H6N6)
A/ruddy turnstone/New Jersey/138/1990 (H3N6)
A/duck/Hokkaido/W165/2015 (H11N6)
A/duck/Kagoshima/461219/2013 (H4N6)
A/environment/Kagoshima/KU-ngr-D/2012 (H4N6)
A/pochard/Fukui/131815/2013 (H4N6)
A/duck/Hunan/S10833/2015 (H6N6)
A/duck/Aichi/231009/2014 (H6N6)
A/duck/Hokkaido/W150/2014 (H6N6)
A/Anas platyrhynchos/Belgium/10399/2018 (H4N6)
A/pintail/Egypt/MB-D-384C/2015 (H3N6)
A/duck/Hokkaido/10/2015 (H3N6)
A/duck/Bangladesh/26948/2015 (H3N6)
A/Wild Duck/South Korea/KNU2020-137/2020 (H4N6)
A/Caspian seal/Russia/T1/2012(H4N6)
A/mallard duck/Netherlands/30/2014 (H4N6)
A/duck/Moscow/5712U/2019 (H11N6)
A/Spoonbill/HK/17-18259/2017 (H5N6)
A/duck/Vietnam/OIE-0155/2012 (H4N6)
A/environment/Kagoshima/KU-G9/2023 (H10N6)
A/environment/Kagoshima/KU-H4/2023 (H10N6)
A/environment/Kagoshima/KU-H5/2023 (H10N6)
N6-II
A/duck/Bangladesh/41481/2019 (H4N6)
A/Common Teal/Dagestan/34d/2019 (H4N6)
A/environment/Kagoshima/KU-H3/2022 (H10N6)
A/environment/Kagoshima/KU-C2/2022 (H10N6)
A/environment/Kagoshima/KU-B15/2022 (H10N6)
N6-I
A/bird/Japan/NIES-0000006/2022 (H4N6)
A/Common Teal/Kyoto/NIES187/2021 (H3N6)
A/environment/Kagoshima/KU-J6/2020 (H3N6)
A/duck/Mongolia/543/2015(H4N6)
A/duck/Mongolia/619/2019 (H3N6)
A/duck/Mongolia/MN18-1/2018 (H3N6)
A/swan/Hokkaido/481107/2017 (H4N6)
A/Duck/Mongolia/2021-MG09/2021 (H4N6)
A/duck/Vietnam/HN5894/2019 (H4N6)
A/mallard/South Korea/JB17-85/2019 (H4N6)
A/duck/Tottori/Vac-T1/2020 (H5N6)
A/black-headed gull/Kamchatka/007/2015 (H13N6)
A/chicken/Viet Nam/QN-0418/2013 (H6N6)
A/Mallard/Peru/M6/2019 (H2N6)
A/Meleagris gallopavo/Valparaiso/11 2 22/2017 (H7N6)
A/Glaucous Gull/Northern Alaska/18AR00289/2018 (H13N6)
A/Red Knot/Delaware Bay/605/2020 (H13N6)
A/mallard/Maryland/UGAI15-8190/2015 (H4N6)
A/mallard/Maryland/UGAI16-5544/2016 (H3N6)
A/environment/Maryland/12OS1506/2012 (H3N6)
A/blue-winged teal/Ohio/907/2006 (H1N6)
A/redhead/Ohio/13OS0399/2013(H3N6)
A/ruddy turnstone/New Jersey/Sg-00534/2008 (H4N6)
A/blue-winged teal/Canada/3240/2011 (H4N6)
A/long-tailed duck/Wisconsin/18OS3407/2018 (H3N6)
A/Mallard duck/Alberta/87/2019 (H4N6)
A/ruddy turnstone/Delaware Bay/328/2020 (H4N6)
A/northern pintail/Alaska/830/2014(H3N6)
A/mallard/Minnesota/MN18-WB1-014A/2018 (H4N6)
A/mallard/Minnesota/UGAI18-2011/2018 (H4N6)
0.05

D

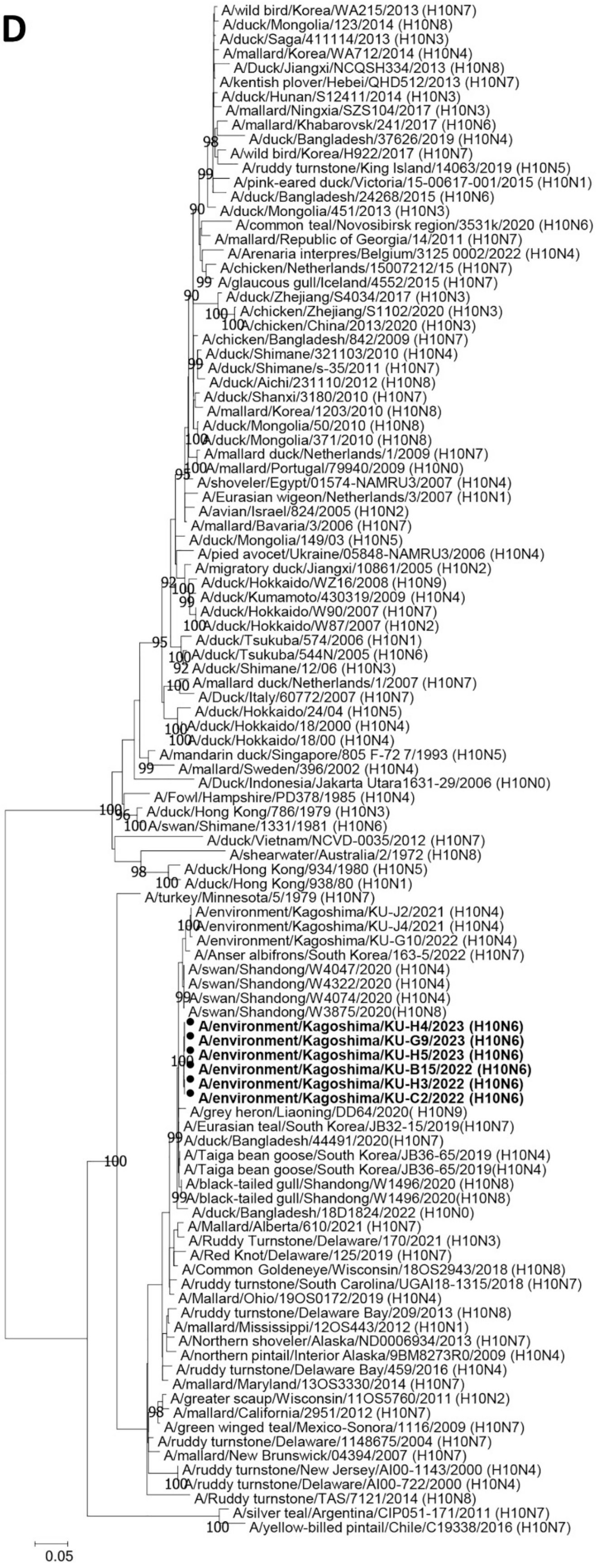
A/wild bird/Korea/WA215/2013 (H10N7)
A/duck/Mongolia/123/2014 (H10N8)
A/duck/Saga/411114/2013 (H10N3)
A/mallard/Korea/WA712/2014 (H10N4)
A/Duck/Jiangxi/NCQSH334/2013 (H10N8)
A/kentish plover/Hebei/QHD512/2013 (H10N7)
A/duck/Hunan/S12411/2014 (H10N3)
A/mallard/Ningxia/SZS104/2017 (H10N3)
A/mallard/Khabarovsk/241/2017 (H10N6)
A/duck/Bangladesh/37626/2019 (H10N4)
A/wild bird/Korea/H922/2017 (H10N7)
A/ruddy turnstone/King Island/14063/2019 (H10N5)
A/pink-eared duck/Victoria/15-00617-001/2015 (H10N1)
A/duck/Bangladesh/24268/2015 (H10N6)
A/duck/Mongolia/451/2013 (H10N3)
A/common teal/Novosibirsk region/3531k/2020 (H10N6)
A/mallard/Republic of Georgia/14/2011 (H10N7)
A/Arenaria interpres/Belgium/3125 0002/2022 (H10N4)
A/chicken/Netherlands/15007212/15 (H10N7)
A/glaucous gull/Iceland/4552/2015 (H10N7)
A/duck/Zhejiang/S4034/2017 (H10N3)
A/chicken/Zhejiang/S1102/2020 (H10N3)
A/chicken/China/2013/2020 (H10N3)
A/chicken/Bangladesh/842/2009 (H10N7)
A/duck/Shimane/321103/2010 (H10N4)
A/duck/Shimane/s-35/2011 (H10N7)
A/duck/Aichi/231110/2012 (H10N8)
A/duck/Shanxi/3180/2010 (H10N7)
A/mallard/Korea/1203/2010 (H10N8)
A/duck/Mongolia/50/2010 (H10N8)
A/duck/Mongolia/371/2010 (H10N8)
A/mallard duck/Netherlands/1/2009 (H10N7)
A/mallard/Portugal/79940/2009 (H10N0)
A/shoveler/Egypt/01574-NAMRU3/2007 (H10N4)
A/Eurasian wigeon/Netherlands/3/2007 (H10N1)
A/avian/Israel/824/2005 (H10N2)
A/mallard/Bavaria/3/2006 (H10N7)
A/duck/Mongolia/149/03 (H10N5)
A/pied avocet/Ukraine/05848-NAMRU3/2006 (H10N4)
A/migratory duck/Jiangxi/10861/2005 (H10N2)
A/duck/Hokkaido/WZ16/2008 (H10N9)
A/duck/Kumamoto/430319/2009 (H10N4)
A/duck/Hokkaido/W90/2007 (H10N7)
A/duck/Hokkaido/W87/2007 (H10N2)
A/duck/Tsukuba/574/2006 (H10N1)
A/duck/Tsukuba/544N/2005 (H10N6)
A/duck/Shimane/12/06 (H10N3)
A/mallard duck/Netherlands/1/2007 (H10N7)
A/Duck/Italy/60772/2007 (H10N7)
A/duck/Hokkaido/24/04 (H10N5)
A/duck/Hokkaido/18/2000 (H10N4)
A/duck/Hokkaido/18/00 (H10N4)
A/mandarin duck/Singapore/805 F-72 7/1993 (H10N5)
A/mallard/Sweden/396/2002 (H10N4)
A/Duck/Indonesia/Jakarta Utara1631-29/2006 (H10N0)
A/Fowl/Hampshire/PD378/1985 (H10N4)
A/duck/Hong Kong/786/1979 (H10N3)
A/swan/Shimane/1331/1981 (H10N6)
A/duck/Vietnam/NCVD-0035/2012 (H10N7)
A/shearwater/Australia/2/1972 (H10N8)
A/duck/Hong Kong/934/1980 (H10N5)
A/duck/Hong Kong/938/80 (H10N1)
A/turkey/Minnesota/5/1979 (H10N7)
A/environment/Kagoshima/KU-J2/2021 (H10N4)
A/environment/Kagoshima/KU-J4/2021 (H10N4)
A/environment/Kagoshima/KU-G10/2022 (H10N4)
A/Anser albifrons/South Korea/163-5/2022 (H10N7)
A/swan/Shandong/W4047/2020 (H10N4)
A/swan/Shandong/W4322/2020 (H10N4)
A/swan/Shandong/W4074/2020 (H10N4)
A/swan/Shandong/W3875/2020(H10N8)
A/environment/Kagoshima/KU-H4/2023 (H10N6)
A/environment/Kagoshima/KU-G9/2023 (H10N6)
A/environment/Kagoshima/KU-H5/2023 (H10N6)
A/environment/Kagoshima/KU-B15/2022 (H10N6)
A/environment/Kagoshima/KU-H3/2022 (H10N6)
A/environment/Kagoshima/KU-C2/2022 (H10N6)
A/grey heron/Liaoning/DD64/2020(H10N9)
A/Eurasian teal/South Korea/JB32-15/2019(H10N7)
A/duck/Bangladesh/44491/2020(H10N7)
A/Taiga bean goose/South Korea/JB36-65/2019 (H10N4)
A/Taiga bean goose/South Korea/JB36-65/2019(H10N4)
A/black-tailed gull/Shandong/W1496/2020 (H10N8)
A/black-tailed gull/Shandong/W1496/2020(H10N8)
A/duck/Bangladesh/18D1824/2022 (H10N0)
A/Mallard/Alberta/610/2021 (H10N7)
A/Ruddy Turnstone/Delaware/170/2021 (H10N3)
A/Red Knot/Delaware/125/2019 (H10N7)
A/Common Goldeneye/Wisconsin/18OS2943/2018 (H10N8)
A/ruddy turnstone/South Carolina/UGAI18-1315/2018 (H10N7)
A/Mallard/Ohio/19OS0172/2019 (H10N4)
A/ruddy turnstone/Delaware Bay/209/2013 (H10N8)
A/mallard/Mississippi/12OS443/2012 (H10N1)
A/Northern shoveler/Alaska/ND0006934/2013 (H10N7)
A/northern pintail/Interior Alaska/9BM8273R0/2009 (H10N4)
A/ruddy turnstone/Delaware Bay/459/2016 (H10N4)
A/mallard/Maryland/13OS3330/2014 (H10N7)
A/greater scaup/Wisconsin/11OS5760/2011 (H10N2)
A/mallard/California/2951/2012 (H10N7)
A/green winged teal/Mexico-Sonora/1116/2009 (H10N7)
A/ruddy turnstone/Delaware/1148675/2004 (H10N7)
A/mallard/New Brunswick/04394/2007 (H10N7)
A/ruddy turnstone/New Jersey/AI00-1143/2000 (H10N4)
A/ruddy turnstone/Delaware/AI00-722/2000 (H10N4)
A/Ruddy turnstone/TAS/7121/2014 (H10N8)
A/silver teal/Argentina/CIP051-171/2011 (H10N7)
A/yellow-billed pintail/Chile/C19338/2016 (H10N7)
0.05

E

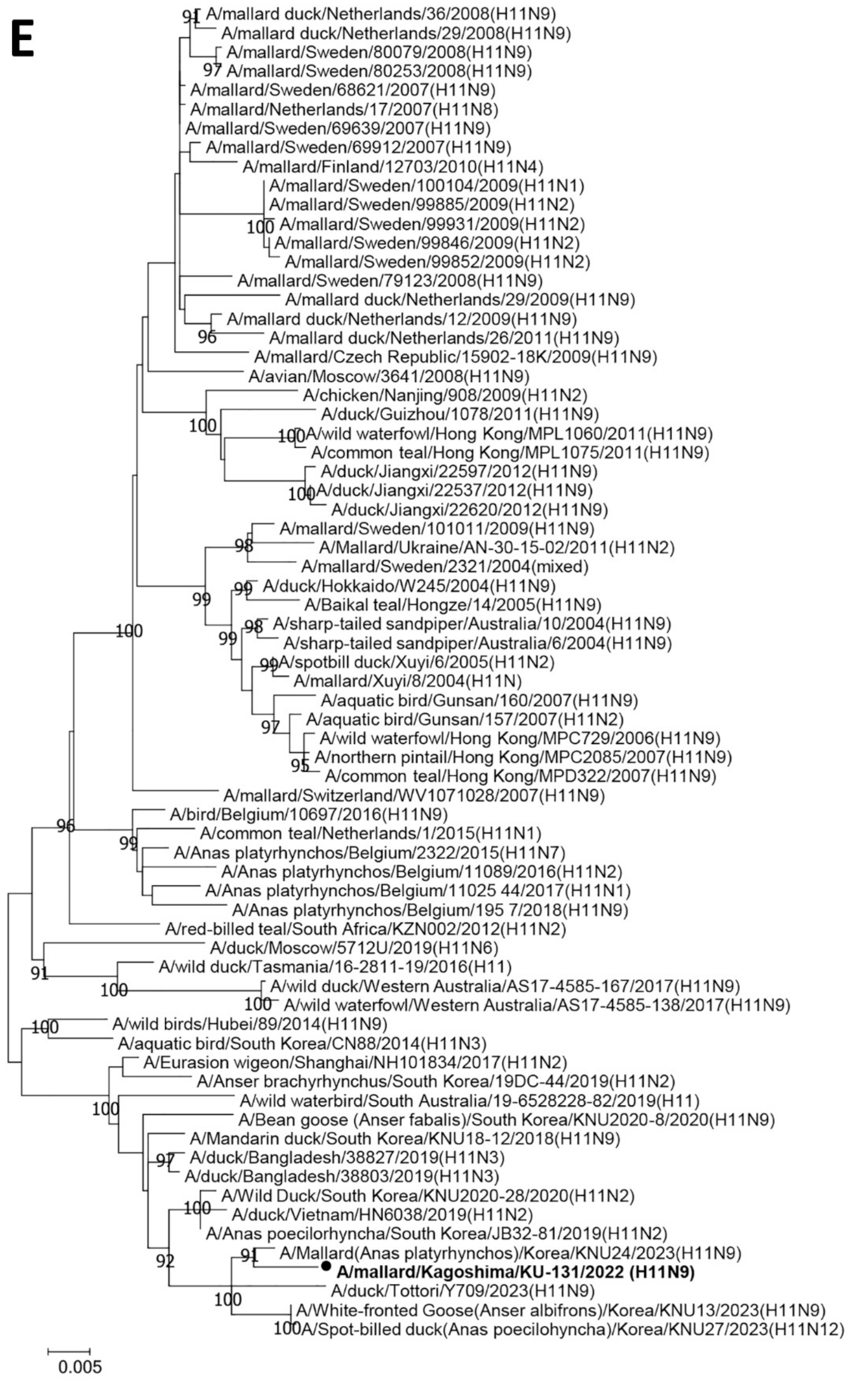
A/mallard duck/Netherlands/36/2008(H11N9)
A/mallard duck/Netherlands/29/2008(H11N9)
A/mallard/Sweden/80079/2008(H11N9)
A/mallard/Sweden/80253/2008(H11N9)
A/mallard/Sweden/68621/2007(H11N9)
A/mallard/Netherlands/17/2007(H11N8)
A/mallard/Sweden/69639/2007(H11N9)
A/mallard/Sweden/69912/2007(H11N9)
A/mallard/Finland/12703/2010(H11N4)
A/mallard/Sweden/100104/2009(H11N1)
A/mallard/Sweden/99885/2009(H11N2)
A/mallard/Sweden/99931/2009(H11N2)
A/mallard/Sweden/99846/2009(H11N2)
A/mallard/Sweden/99852/2009(H11N2)
A/mallard/Sweden/79123/2008(H11N9)
A/mallard duck/Netherlands/29/2009(H11N9)
A/mallard duck/Netherlands/12/2009(H11N9)
A/mallard duck/Netherlands/26/2011(H11N9)
A/mallard/Czech Republic/15902-18K/2009(H11N9)
A/avian/Moscow/3641/2008(H11N9)
A/chicken/Nanjing/908/2009(H11N2)
A/duck/Guizhou/1078/2011(H11N9)
A/wild waterfowl/Hong Kong/MPL1060/2011(H11N9)
A/common teal/Hong Kong/MPL1075/2011(H11N9)
A/duck/Jiangxi/22597/2012(H11N9)
A/duck/Jiangxi/22537/2012(H11N9)
A/duck/Jiangxi/22620/2012(H11N9)
A/mallard/Sweden/101011/2009(H11N9)
A/Mallard/Ukraine/AN-30-15-02/2011(H11N2)
A/mallard/Sweden/2321/2004(mixed)
A/duck/Hokkaido/W245/2004(H11N9)
A/Baikal teal/Hongze/14/2005(H11N9)
A/sharp-tailed sandpiper/Australia/10/2004(H11N9)
A/sharp-tailed sandpiper/Australia/6/2004(H11N9)
A/spotbill duck/Xuyi/6/2005(H11N2)
A/mallard/Xuyi/8/2004(H11N)
A/aquatic bird/Gunsan/160/2007(H11N9)
A/aquatic bird/Gunsan/157/2007(H11N2)
A/wild waterfowl/Hong Kong/MPC729/2006(H11N9)
A/northern pintail/Hong Kong/MPC2085/2007(H11N9)
A/common teal/Hong Kong/MPD322/2007(H11N9)
A/mallard/Switzerland/WV1071028/2007(H11N9)
A/bird/Belgium/10697/2016(H11N9)
A/common teal/Netherlands/1/2015(H11N1)
A/Anas platyrhynchos/Belgium/2322/2015(H11N7)
A/Anas platyrhynchos/Belgium/11089/2016(H11N2)
A/Anas platyrhynchos/Belgium/11025 44/2017(H11N1)
A/Anas platyrhynchos/Belgium/195 7/2018(H11N9)
A/red-billed teal/South Africa/KZN002/2012(H11N2)
A/duck/Moscow/5712U/2019(H11N6)
A/wild duck/Tasmania/16-2811-19/2016(H11)
A/wild duck/Western Australia/AS17-4585-167/2017(H11N9)
A/wild waterfowl/Western Australia/AS17-4585-138/2017(H11N9)
A/wild birds/Hubei/89/2014(H11N9)
A/aquatic bird/South Korea/CN88/2014(H11N3)
A/Eurasion wigeon/Shanghai/NH101834/2017(H11N2)
A/Anser brachyrhynchus/South Korea/19DC-44/2019(H11N2)
A/wild waterbird/South Australia/19-6528228-82/2019(H11)
A/Bean goose (Anser fabalis)/South Korea/KNU2020-8/2020(H11N9)
A/Mandarin duck/South Korea/KNU18-12/2018(H11N9)
A/duck/Bangladesh/38827/2019(H11N3)
A/duck/Bangladesh/38803/2019(H11N3)
A/Wild Duck/South Korea/KNU2020-28/2020(H11N2)
A/duck/Vietnam/HN6038/2019(H11N2)
A/Anas poecilorhyncha/South Korea/JB32-81/2019(H11N2)
A/Mallard(Anas platyrhynchos)/Korea/KNU24/2023(H11N9)
A/mallard/Kagoshima/KU-131/2022 (H11N9)
A/duck/Tottori/Y709/2023(H11N9)
A/White-fronted Goose(Anser albifrons)/Korea/KNU13/2023(H11N9)
A/Spot-billed duck(Anas poecilohyncha)/Korea/KNU27/2023(H11N12)
91
97
100
96
100
100
100
98
99
99
99
98
99
97
95
100
96
99
91
100
100
100
100
97
100
92
91
100
100
0.005

F

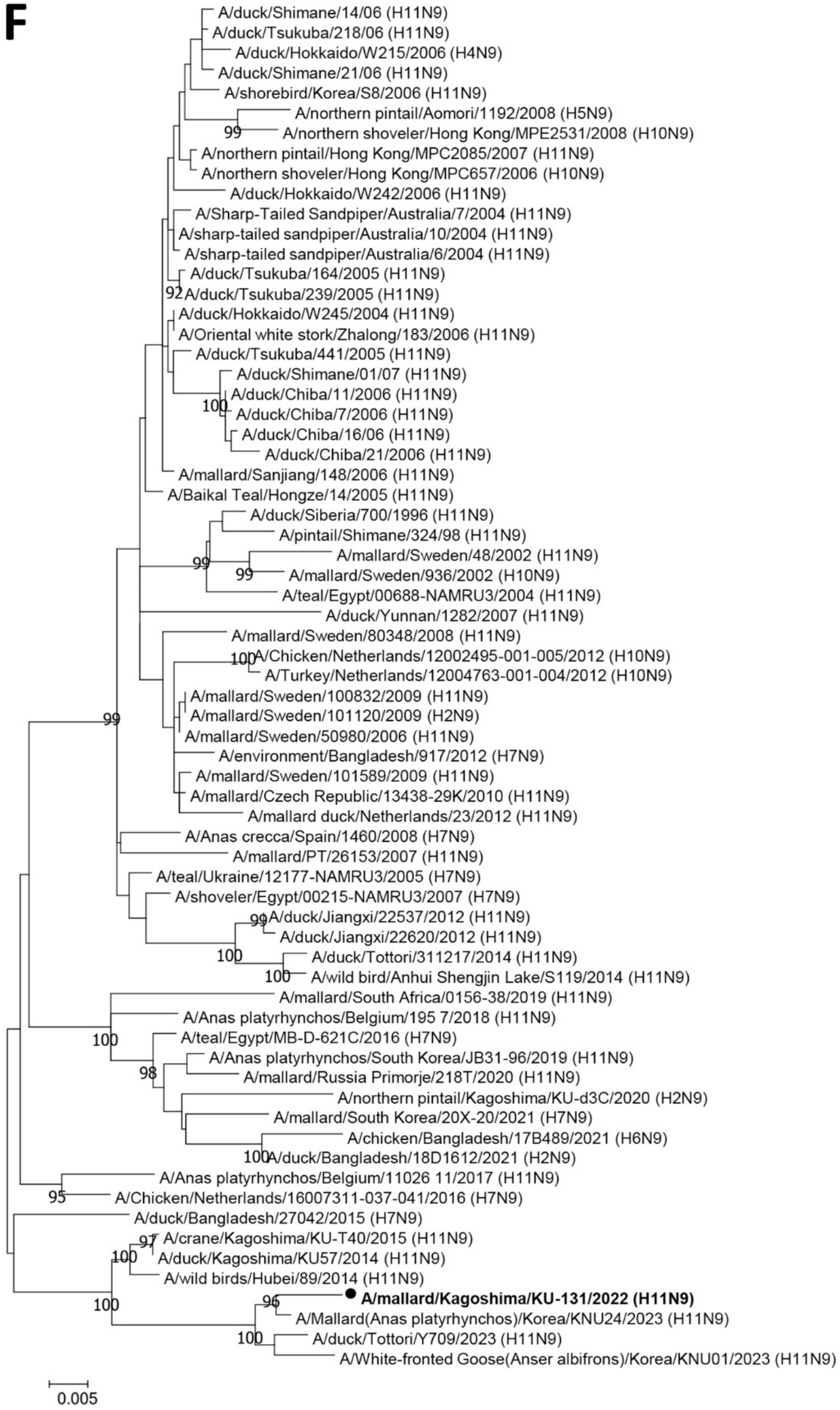

A/duck/Shimane/14/06 (H11N9)
A/duck/Tsukuba/218/06 (H11N9)
A/duck/Hokkaido/W215/2006 (H4N9)
A/duck/Shimane/21/06 (H11N9)
A/shorebird/Korea/S8/2006 (H11N9)
A/northern pintail/Aomori/1192/2008 (H5N9)
A/northern shoveler/Hong Kong/MPE2531/2008 (H10N9)
A/northern pintail/Hong Kong/MPC2085/2007 (H11N9)
A/northern shoveler/Hong Kong/MPC657/2006 (H10N9)
A/duck/Hokkaido/W242/2006 (H11N9)
A/Sharp-Tailed Sandpiper/Australia/7/2004 (H11N9)
A/sharp-tailed sandpiper/Australia/10/2004 (H11N9)
A/sharp-tailed sandpiper/Australia/6/2004 (H11N9)
A/duck/Tsukuba/164/2005 (H11N9)
A/duck/Tsukuba/239/2005 (H11N9)
A/duck/Hokkaido/W245/2004 (H11N9)
A/Oriental white stork/Zhalong/183/2006 (H11N9)
A/duck/Tsukuba/441/2005 (H11N9)
A/duck/Shimane/01/07 (H11N9)
A/duck/Chiba/11/2006 (H11N9)
A/duck/Chiba/7/2006 (H11N9)
A/duck/Chiba/16/06 (H11N9)
A/duck/Chiba/21/2006 (H11N9)
A/mallard/Sanjiang/148/2006 (H11N9)
A/Baikal Teal/Hongze/14/2005 (H11N9)
A/duck/Siberia/700/1996 (H11N9)
A/pintail/Shimane/324/98 (H11N9)
A/mallard/Sweden/48/2002 (H11N9)
A/mallard/Sweden/936/2002 (H10N9)
A/teal/Egypt/00688-NAMRU3/2004 (H11N9)
A/duck/Yunnan/1282/2007 (H11N9)
A/mallard/Sweden/80348/2008 (H11N9)
A/Chicken/Netherlands/12002495-001-005/2012 (H10N9)
A/Turkey/Netherlands/12004763-001-004/2012 (H10N9)
A/mallard/Sweden/100832/2009 (H11N9)
A/mallard/Sweden/101120/2009 (H2N9)
A/mallard/Sweden/50980/2006 (H11N9)
A/environment/Bangladesh/917/2012 (H7N9)
A/mallard/Sweden/101589/2009 (H11N9)
A/mallard/Czech Republic/13438-29K/2010 (H11N9)
A/mallard duck/Netherlands/23/2012 (H11N9)
A/Anas crecca/Spain/1460/2008 (H7N9)
A/mallard/PT/26153/2007 (H11N9)
A/teal/Ukraine/12177-NAMRU3/2005 (H7N9)
A/shoveler/Egypt/00215-NAMRU3/2007 (H7N9)
A/duck/Jiangxi/22537/2012 (H11N9)
A/duck/Jiangxi/22620/2012 (H11N9)
A/duck/Tottori/311217/2014 (H11N9)
A/wild bird/Anhui Shengjin Lake/S119/2014 (H11N9)
A/mallard/South Africa/0156-38/2019 (H11N9)
A/Anas platyrhynchos/Belgium/195 7/2018 (H11N9)
A/teal/Egypt/MB-D-621C/2016 (H7N9)
A/Anas platyrhynchos/South Korea/JB31-96/2019 (H11N9)
A/mallard/Russia Primorje/218T/2020 (H11N9)
A/northern pintail/Kagoshima/KU-d3C/2020 (H2N9)
A/mallard/South Korea/20X-20/2021 (H7N9)
A/chicken/Bangladesh/17B489/2021 (H6N9)
A/duck/Bangladesh/18D1612/2021 (H2N9)
A/Anas platyrhynchos/Belgium/11026 11/2017 (H11N9)
A/Chicken/Netherlands/16007311-037-041/2016 (H7N9)
A/duck/Bangladesh/27042/2015 (H7N9)
A/crane/Kagoshima/KU-T40/2015 (H11N9)
A/duck/Kagoshima/KU57/2014 (H11N9)
A/wild birds/Hubei/89/2014 (H11N9)
A/mallard/Kagoshima/KU-131/2022 (H11N9)
A/Mallard(Anas platyrhynchos)/Korea/KNU24/2023 (H11N9)
A/duck/Tottori/Y709/2023 (H11N9)
A/White-fronted Goose(Anser albifrons)/Korea/KNU01/2023 (H11N9)
0.005

Appendix Figure 4. Phylogenetic trees of H3 HA, N8 NA, N6 NA, H10 HA, H11 HA, and N9 NA genes. Phylogenetic trees of H3 HA (A), N8 NA (B), N6 NA (C), H10 HA (D), H11 HA (E), and N9 NA (F) gene segments were constructed. AIVs isolated in this study are marked with black circles. The maximum - ikelihood method with a bootstrapping set of 1,000 replicates was employed, and nodes with bootstrap values exceeding 90% are displayed. The scale bar represents the number of nucleotide substitutions per site. HA, hemagglutinin; NA, neuraminidase.
